# Advances in the Synthetic Approaches to β‑Secretase (BACE-1) Inhibitors in Countering Alzheimer’s: A Comprehensive Review

**DOI:** 10.1021/acsomega.5c04467

**Published:** 2025-08-06

**Authors:** Yogeesh M, Nitinkumar S. Shetty

**Affiliations:** Department of Chemistry, Manipal Institute of Technology, 76793 Manipal Academy of Higher Education, Manipal, Karnataka 576104, India

## Abstract

Alzheimer’s disease is a progressive, irreversible, neurodegenerative disease, i.e., characterized by the presence of amyloid plaques, hyperphosphorylated tau protein (hyper p-tau), neural damage, etc. β-amyloid precursor protein cleavage enzyme 1 (BACE-1) inhibition is a promising avenue for slowing AD progression. In a rate-limiting step, BACE-1 cleaves the amyloid precursor protein (APP) into soluble amyloid precursor protein β (sAPPβ) and a membrane-bound C-terminal fragment called C99. γ-secretase processes C99, resulting in neurotoxic amyloid β (Aβ). Selective and potent BACE-1 inhibitors offer promising therapeutic avenues for Alzheimer’s disease. While BACE-1 inhibitors have shown significant assurance as potential treatments for Alzheimer’s disease, many early compounds struggled to advance clinically due to poor brain penetration, limited selectivity, and unwanted side effects. Over the last two decades, substantial progress has been made in the development of BACE-1 inhibitors, leading to the emergence of diverse structural frameworks such as aminohydontoins, dihydropyridines, pyrimidines, and iminohydantoins, and fused heterocycles. This review provides an in-depth analysis of the synthetic strategies employed. It emphasizes the structure–activity relationship (SAR) trends that have guided their optimization and the crystal structure of the enzyme used in the inhibition study.

## Graphical abstract

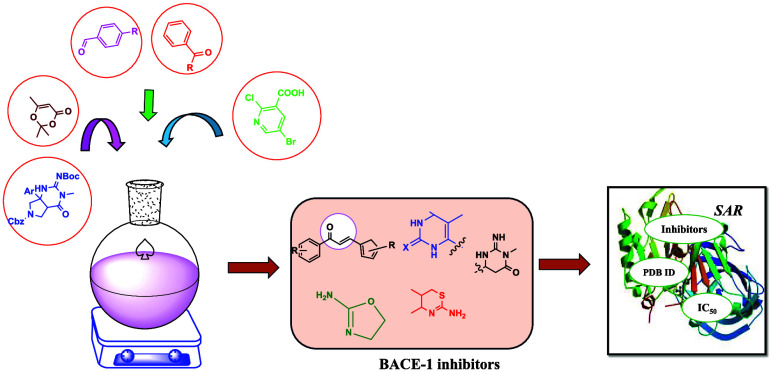

## Introduction

Alzheimer’s disease (AD) is recognized as the predominant type of dementia. It is a neurodegenerative disease that progresses over time and causes changes in behavioral disorders such as apathy, aggression, depression, and mood disturbances. BACE-1 inhibition is a promising avenue for slowing AD progression, which affects a person’s ability to perform daily tasks.^1–3^ AD has been classified as a multifactorial disease, with risk factors including increasing age, genetics, head injuries, vascular diseases, infections, and environmental factors such as heavy and trace metals.^4^ Other symptoms include difficulties speaking, solving problems, and other cognitive skills.^5^ Now, AD is a common form of dementia, and around the world, approximately 55 million people have this disease. According to an estimation, 74.7 million people will have dementia by 2030.^6^ The only medications currently approved for the treatment of AD are *N*-methyl-d-aspartate (NMDA) receptor modulators and cholinesterase inhibitors.^7^ They provide only moderate symptomatic benefits despite helping improve cognitive, behavioral, and functional impairments.^8,9^ Since no therapy effectively stops or even cures the disease, treating the molecular mechanisms underlying the pathogenic processes is necessary for a cure.^10^ It is a well-known fact that the number of people living with dementia increases as the population ages. The primary cause of this type of dementia is thought to be the buildup of insoluble polypeptides in the brain, which results in neurodegenerative plaques.^5^

β-secretase or β-site amyloid precursor protein-cleaving enzyme 1­(BACE-1) is an essential key enzyme involved in the pathology of Alzheimer’s disease (AD).^11^ A type I transmembrane aspartyl protease, BACE-1, can be present in the brain, especially in neurons, oligodendrocytes, and astrocytes.^12–14^ BACE-1 has been found in healthy synaptic terminals and is present at the plasma endothelial membrane and endosomal compartments. APP is an enormous type 1 membrane protein subjected to three enzymes, α-, β-, and γ-secretases, which generate different end products. When α-(place holder 1) secretase and β-secretase compete for the APP substrate, APPsα, a soluble ectodomain, is produced. This leaves behind a C-terminus fragment (C83) bound to the membrane and would be processed into a protein fragment known as P3 by the action of γ-secretase. Because of this, the α-secretase activity does not cause any insoluble Aβ protein to form, and this pathway is called the nonamyloidogenic pathway. On the other hand, the β-secretase (BACE-1) cleaves APP at its N-terminus to initiate the amyloidogenic pathway. This forms two fragments: the membrane-bound C-terminus (C99) and the soluble ectodomain APPsβ. After that, γ-secretase breaks down C99 to produce two different kinds of Aβ proteins: Aβ40 and Aβ42 (Figure 1). Aβ42 is a key player in AD pathogenesis^14^ because Aβ42 is more hydrophobic and stickier than Aβ40 and is overproduced in AD because of genetic mutations in the APP gene or other genes. Aβ42 fibrils readily clump together to form amyloid plaques.^15^ BACE-1 acts as the β-secretase enzyme by cleaving the transmembrane APP to release the β-stubs. This rate-limiting catalytic step produced insoluble Aβ, which aggregates and causes plaque deposition and neurodegeneration, making BACE1 an appealing target for AD treatment. As a result, the development of potent BACE1 inhibitors was mandatory. Many of them reached the late stages of clinical trials. However, the high failure rate of lead drug candidates that targeted BACE-1 highlighted the necessity of identifying alternative targets to solve the puzzle of AD.^14,16^ Targeting the initial stage of amyloid β-production, the BACE-1 inhibitor provides an effective way of disease modification and addresses one of the primary causes of AD. However, there is a development of β-secretase inhibitors associated with the challenges, including selective inhibition of β-secretase over other proteases by enhancing the effectiveness of penetration of the blood-brain barrier (BBB) and minimizing toxicity^17,18^ and off-target effects.

**Figure 1:**
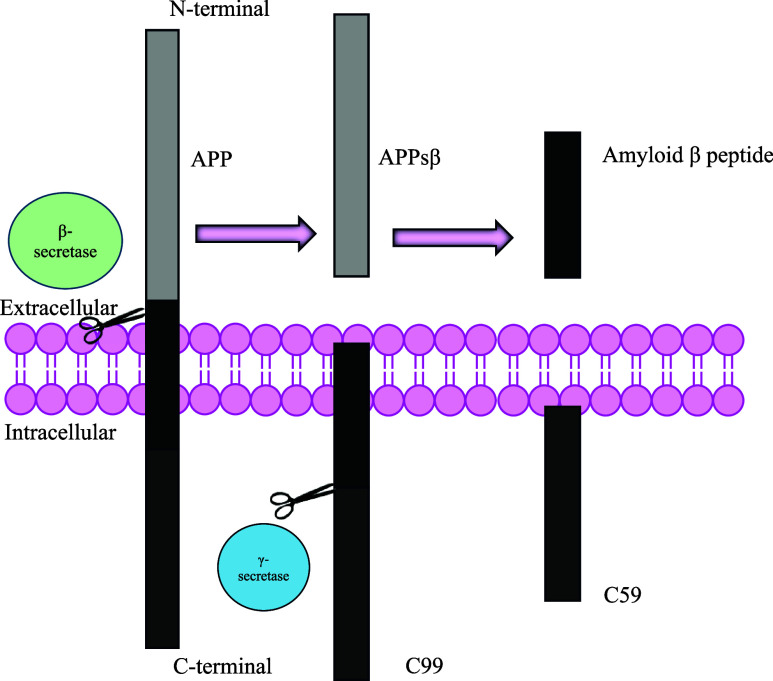
Schematic representation of amyloid precursor protein cleavage by β and γ secretases, leading to the formation of amyloid β peptides.

Over the last two decades, considerable research has been directed toward discovering and developing effective BACE-1 inhibitors. Several promising molecules, including LY2886721, verubecestat (MK-8931), elenbecestat (E2609), and atabecestat (JNJ-54861911), progressed into clinical trials.^19–21^ Despite their ability to significantly lower amyloid-β (Aβ) levels, these candidates were ultimately withdrawn due to issues such as insufficient clinical efficacy, adverse side effects, or undesirable off-target interactions, notably with enzymes like cathepsin D.^17,22^ These setbacks have highlighted the pressing need for more refined drug design approaches that retain potency, enhance target selectivity, improve central nervous system (CNS) penetration, and reduce toxicity.

In the past 14 years, researchers have employed diverse synthetic approaches to create BACE-1 inhibitors with improved CNS bioavailability and therapeutic potential. Efforts have focused on various scaffolds, including aminohydantoins, oxazines, dihydropyridines, iminopyrimidinones, fused heterocycles, and acylguanidines. These scaffolds have been optimized by carefully modulating physicochemical properties to enhance BBB permeability, metabolic stability, and target binding affinity. Advances in structural biology, molecular modeling, and CNS drug design have played a vital role in guiding the rational development of these compounds.

While numerous reviews have addressed the biological roles of BACE-1 and the outcome of clinical trials, fewer have focused on the synthetic evolution and SAR-driven optimization of chemical scaffolds used in BACE-1 inhibitor design. Therefore, the primary objective of this review is to provide a comprehensive overview of the synthetic methodologies, scaffold evolution, and structure–activity relationships (SAR) of BACE-1 inhibitors reported between 2010 and 2024. The review highlights critical synthetic advances, standard pharmacophore features, and medicinal chemistry strategies employed to overcome the limitations that have hindered clinical success.

## Amino/Imino Hydantoin Derivatives as BACE-1 Inhibitors

Amino/Imino hydantoins are organic compounds that have been studied for their diverse, potential therapeutic applications in drug discovery. Based on the presence of –NH_2_ and =NH groups, hydantoin rings are classified as aminohydontoins and iminohydantoins, respectively.^23^ Both hydantoins have been shown to reduce amyloid−β level production in Alzheimer’s disease. Notably, spirocyclic and disubstituted pyridinyl aminohydontoins had shown promising β-secretase inhibition with specificity and brain penetration.^24–26^

Malamas et al. synthesized a novel disubstituted pyridinyl amino hydantoins as a BACE-1 inhibitor by considering amino hydantoins as a lead molecule.^24^ The synthesis of acetylene intermediate **5** was achieved via two routes. In route (a), Sonogashira coupling 4-ethylpyridine **1** with 1-bromo-3-iodobenzene yields 75% of acetylene **5**. In route (b), Palladium-catalyzed cross-coupling of **2** with trimethyl silane, followed by hydrolysis, yields phenyl acetylene **4**. Subsequent Sonogashira coupling of **4** with bromopyridine yields 45% of acetylene **5**. In the presence of sodium bicarbonate and magnesium sulfate, the oxidation of **5**, with potassium permanganate, afforded diketone **6** with a yield of 75%. Treatment of diketone **6** with 1-methyl guanidine and potassium carbonate led to the formation of aminohydontoins **7**. Pd (0) or Pd (II) catalyzes a cross-coupling reaction of **7** with heteroaryl boronic acid **8** to produce desired disubstituted amino hydantoins **9** (45–75% yield) (Scheme 1). The SAR analysis revealed that structural modifications around the pyridine ring within the S2′ binding pocket showed that 2,6-disubstitution (e.g., ethyl groups) markedly enhanced the BACE-1 selectivity by exploiting a key difference between BACE-1 (Pro70) and BACE-2 (Lys86). Incorporating heteroaryl groups at the S3 region, such as pyrimidines and 2-fluoropyridines on an aminohydantoin scaffold, improved the binding through key interactions with the amino acid residue Ser 229 and Trp 76. Inhibitor **9** (if X = N, Y = C, R = F, AND R_1_, R_2_ = Et) (Table 1) exhibited potent BACE-1 inhibition with an IC_50_ value of 10 nM with 500-fold selectivity over BACE-2 and cathepsin D.

**Scheme 1:**
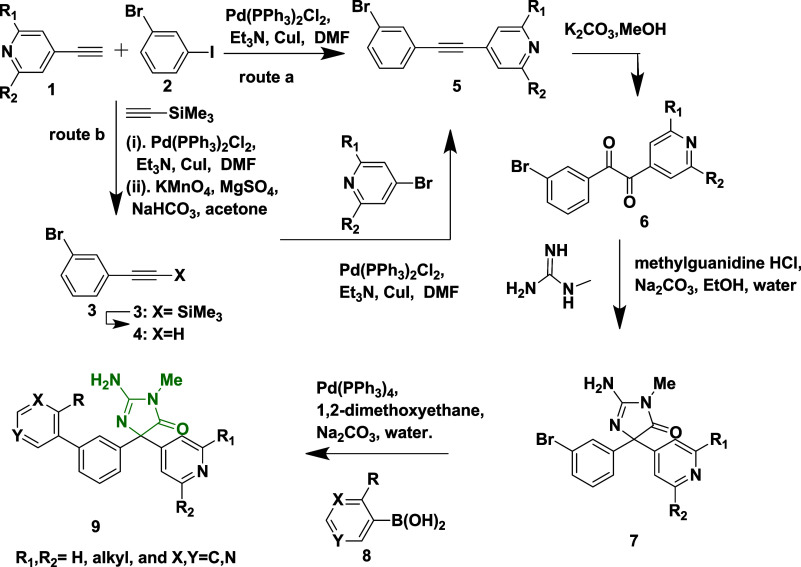
Synthesis of Pyridine-Amino Hydantoins **9**

**Table 1:**
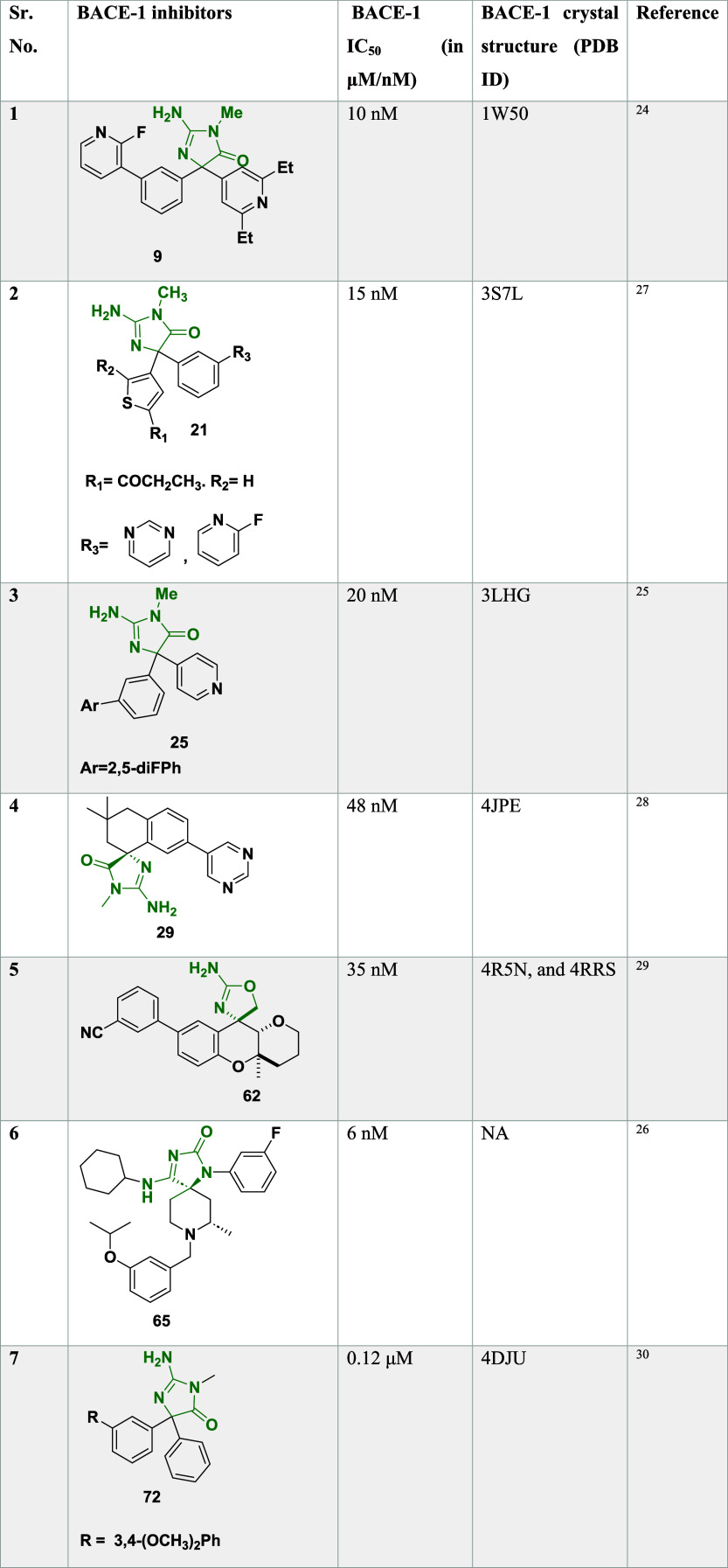
BACE-1 Inhibitors, the Co-Crystal Structure (PDB ID) of BACE-1 Enzyme, and Their IC_50_ Values

In another report, Malamas et al. synthesized new thienyl and pyrazolyl aminohydontoin as a β-secretase inhibitor.^27^ Synthesis involved the iodination of pyrazole **10** with iodine and ammonium hexanitratocerium­(IV), producing 4-iodopyrazole **11**. In the presence of sodium hydride, **11** was subsequently alkylated with different alkyl halides to yield an intermediate alkylated 4-iodopyrazole **12**. As an alternative, the pyrazole nitrogen was protected by sodium hydride and 2-(trimethylsilyl)­ethoxy methyl chloride (SEM-chloride), which allowed N-substituents to be added. Acetylenes **13** were produced via Sonogashira coupling reaction of pyrazoles with 3-bromophenyl acetylene in the presence of triethylamine, bis­(triphenylphosphine)­palladium­(II) chloride, and copper iodine. Diketones **14** were synthesized by oxidation of acetylenes **13** with potassium permanganate, while magnesium sulfate and sodium bicarbonate were present. These 1,2-disubstituted diketones are then converted into aminohydontoins **15** via a condensation reaction with 1-methyl guanidine in potassium carbonate. The cross-coupling reaction of amino hydantoins **15** with different heteroaryl boronic acids (Suzuki coupling) was catalyzed by palladium, producing the desired pyrazolyl aminohydontoins **16** (Scheme 2). Similarly, thionyl aminohydontoins **21** were synthesized by substituting 4-iodothiophene **17** instead of 4-iodopyrazole **12** (Scheme 3). The thienyl and pyrazolyl-containing derivatives, which include a keto functional group, enhanced the potential of the inhibitor. The SAR studies revealed that modifying the S2′ region of BACE-1 inhibitors using thienyl and pyrazolyl substituents on an aminohydantoin scaffold enhances activity and selectivity. The pyrazole ring, which contains small *N*-alkyl groups, improved potency and selectivity, while bulkier hydrophobic groups enhanced BACE-1 affinity but reduced BACE-2 selectivity. A 5-keto group on the thiophene ring significantly enhanced the binding potency through key interactions with Trp76. Although pyridine analogs were more potent than pyrimidines, they showed lower selectivity. The pyrimidine thiophene derivative **21** (if R_1_ = COCH_2_CH_3_, R_2_ = H, and R_3_ = pyrimidine) and the pyridinyl thiophene derivative **21** (if R_1_ = COEt, R_2_ = H, and R_3_ = 2-fluoropyridine) are the two potent inhibitors (Table 1) among these series with IC_50_ values in the range of 15 nM. However, in vivo studies revealed that the limited brain permeability of these inhibitors may limit the reduction of β-amyloid levels.

**Scheme 2:**
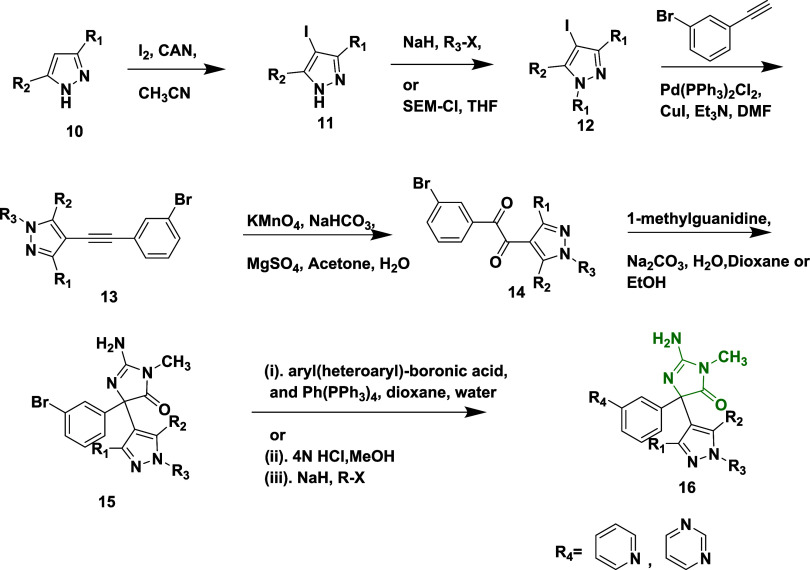
Synthesis of Pyrazolyl Aminohydontoins **16**

**Scheme 3:**
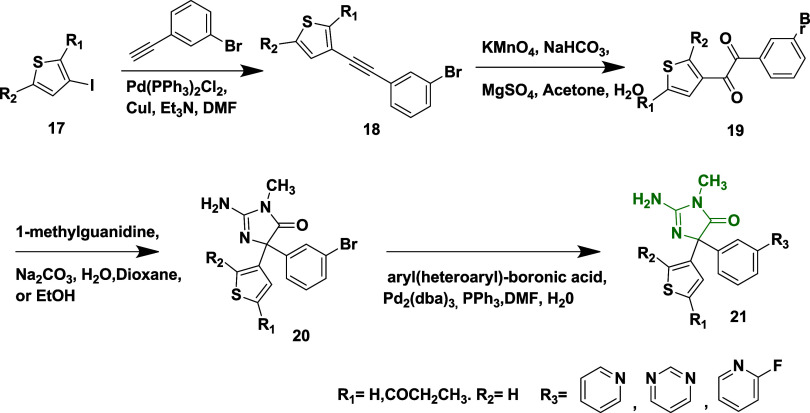
Synthesis of Thionyl Aminohydontoins **21**

Considering aminohydontoins as lead molecules, Zhou et al. reported the synthesis of a novel, small molecule of pyridinyl amino hydantoins as a β-secretase inhibitor,^25^ which involved the oxidation of 4-(3-bromophenylethynyl) pyridine **22** with potassium permanganate in the presence of sodium bicarbonate and magnesium sulfate to afford diketone **23**. The compound 3-bromophenylpyridinyl hydantoins **24** is formed via condensation of **23** with *N*-methyl guanidine in the presence of sodium carbonate, followed by Suzuki coupling reaction with various boronic acids, yielding pyridinyl amino hydantoins **25** with yields in the range of 51–95% (Scheme 4). From SAR analysis, it was found that incorporation of a phenyl ring with electron-withdrawing groups such as cyano and difluorophenyl at the 3-position greatly enhanced the activity, and incorporation of heteroaryl groups (e.g., pyrazinyl, pyridinyl) further improved the potency and selectivity. The most potent compound, **25** (if Ar = 2,5-diFPh) (Table 1), was the potent inhibitor with an IC_50_ value of 20 nM by the interaction between the tryptophan Trp76 with the nitrogen of pyridine in the S2′ region of the enzyme, showing good selectivity over BACE-2 and cathepsin D.

**Scheme 4:**
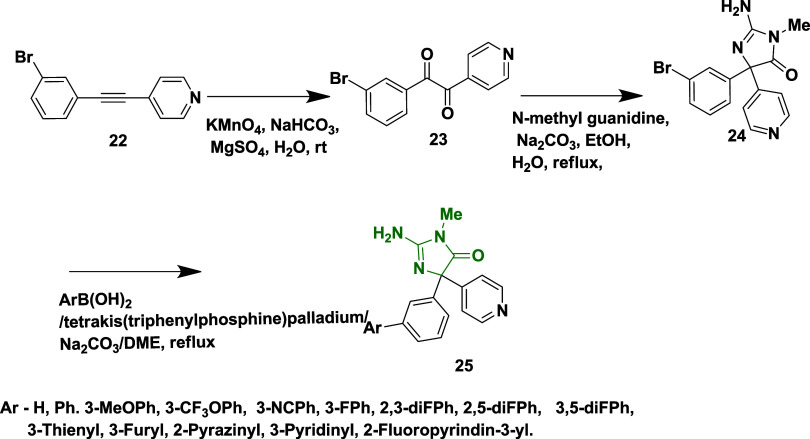
Synthesis of Pyridinyl Amino Hydantoins **25**

Hunt et al. reported the synthesis of a novel, potent, and brain-penetrating inhibitor of the β-secretase enzyme.^28^ i.e., Aminohydontoins and their derivatives were synthesized through the Bucherer-Bergs reaction of 6-Bromo-2,2-dimethylchroman-4-one **26**, followed by the selective alkylation of imide N, afforded intermediate **27**. It was further treated with Lawesson’s reagent and followed by oxidative amination, resulting in desired aminohydontoins **29**, yields ranging from 20 to 70% (Scheme 5).

**Scheme 5:**
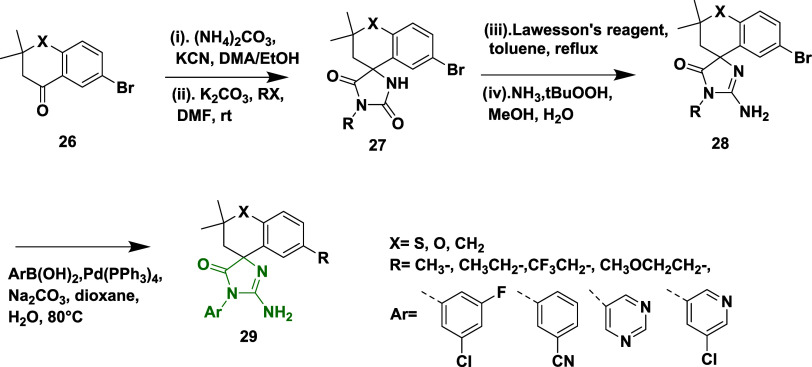
Synthesis of Aminohydontoins **29**

The compound spiroimidazolone derivative **31** was synthesized from the benzylic oxidation of 7′-Bromospiroimidazolone **30** using chromium trioxide and *tert*-butyl hydroperoxide (BuOOH). Then, **31** on Suzuki coupling reaction of **31** with 5-pyrimidinylboronic acid **32** in the presence of palladium dichloride and sodium carbonate, yields tetrahydronaphthalone analogs **33**. Similarly, **31** reacts with bis­(2-methoxyethyl) amino sulfur trifluoride to produce **34**, which was followed by a Suzuki coupling reaction, yielding difluoronaphthalene analogs **35** with a percentage yield in the range of 15–98% (Scheme 6).^28^ This spirocyclic inhibitor is unique for exploring limited BBB and BACE-1 selectivity. SAR analysis revealed that introducing meta-substituted aryl groups like 3-chlorophenyl and 3-cyanophenyl groups on the 6-position of the chroman ring, as seen in compound **29**, which showed enhanced fit in the S3 pocket, significantly improved the BACE-1 potency and favorable water-bridged interactions. Pyrimidine analogues such as 3-pyridyl and 5-chloropyridin-3-yl showed selectivity over cathepsin D but reduced potency, and efforts to minimize efflux often compromised activity. The (*R*)-enantiomers consistently exhibited greater activity than their (*S*)-counterparts, and compound **29** notably reduced CSF Aβ_1–40_ concentrations in vivo, demonstrating its efficacy within the central nervous system. The inhibitor has efflux liability, which limits the permeability of the compound. The most potent compound in this series is **29** (X = CH_2_, Ar = pyrimidine) (Table 1) with an IC_50_ value of 48 nM.

**Scheme 6:**
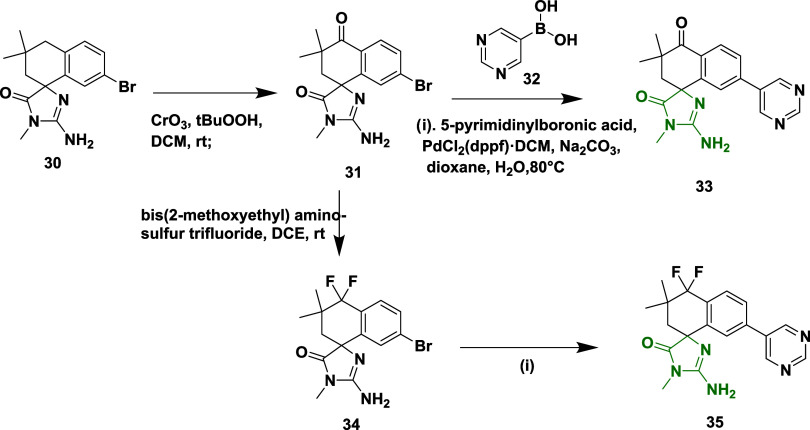
Synthesis of Tetrahydronaphthalone **33** and Difluoronaphthalene **35**

Thomas et al. reported the synthesis of a new class of chromene analogs as a potential β-secretase inhibitor.^29^ Synthesis begins with the condensation of morpholine with pyranone **36**, leading to the formation of enamine **37**. Subsequent addition of **37** to aromatic aldehyde **38**, followed by intramolecular aminal formation, yielded an intermediate 8-bromo-4a-morpholino-2,3,4,4a,10,10a-hexahydropyrano­[3,2-*b*]­chromen-10-ol **39**. An intermediate **39** converted to intermediate **40** via oxidation in the presence of Dess-Martin periodinane, followed by L-selective reduction of 40, yielded chromene **41**. Chromene **41** then reacts with ammonium carbonate and potassium cyanide in the presence of sodium bisulfite, followed by methylation, resulting in **43**. The reaction of **43** with Lawson’s reagent produced a pair of diastereomers 44a and 44b. Further, it was subjected to oxidation/aminolysis in the presence of *tert*-butyl hydroperoxide, ammonium hydroxide, and sodium bisulfite, producing **45a** and **45b**. Finally, **45a** and **45b** underwent tetrakis­(triphenylphosphine)palladium (0) catalyzed Suzuki coupling reactions, to yield chromene derivatives **46a** and **46b** in 15% yield (Scheme 7).

**Scheme 7:**
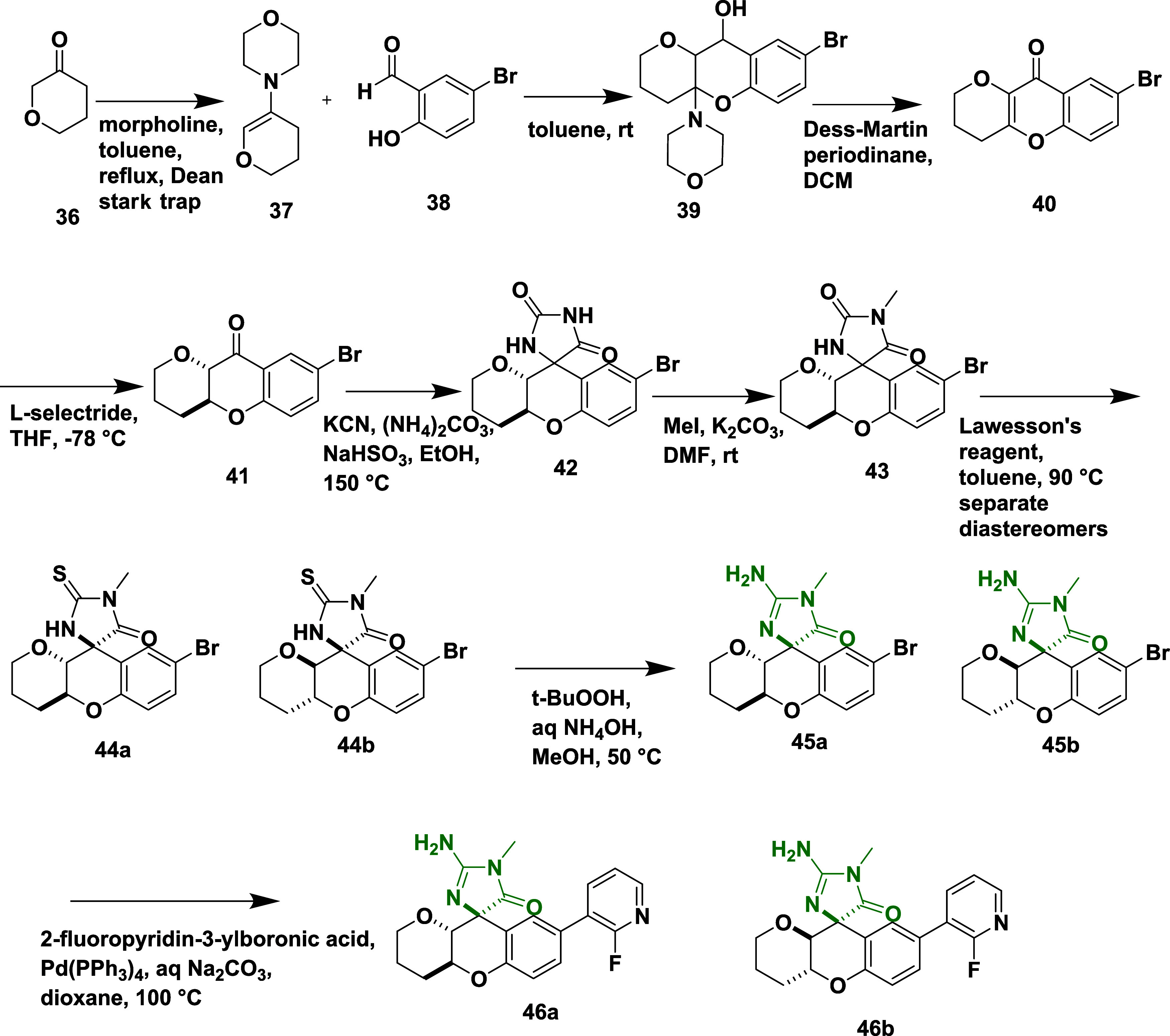
Synthesis of Isomers of Hexahydropyrano­[3,2-b] Chromene Aminohydontoins **46**

*tert*-Butyldimethylsilyl ether **48** was reacted with aromatic ketone **49** in the presence of pyrrolidine, undergoing intramolecular cyclization, to afford chroman **50**. The chroman **50** undergoes deprotonation in the presence of sodium ethoxide and reacts with ethyl formate, yielding 2-(3-((*tert*-butyldimethylsilyl)­oxy)­propyl)-3-(hydroxymethylene)-6-methoxy-2-methylchroman-4-one **51**. The reaction of **51** with naphthalene-2-sulfonyl azide and diethyl amine introduced an azide functionality, followed by the removal of tris-buffered saline (TBS) by treating it with tetra-*n*-butylammonium fluoride (TBAF) and rhodium­(II) catalyzed cyclization, yielded intermediate **52**. An intermediate **52** was then subjected to sequential treatment with ammonium carbonate, sodium bisulfite, methyl iodide, and Lawesson’s reagent to yield (4a*R*)-8-methoxy-4a-methyl-3,4,4a,10a-tetrahydropyrano­[3,2-*b*]­chromen-10­(2*H*)-one **53**. Compound **53**, was then subjected to a multistep transformation involving boron tribromide, dimethylformamide dimethyl acetal, *N*-phenyl-bis (trifluoromethane sulfonyl) aniline, and triethylamine, yielded (4*S*, 4a′*S*, 10a′*R*)-2-(((dimethylamino)­methylene)­amino)-1,4a′-dimethyl-5-oxo-1,3′,4′,4a′, 5,10a′-hexahydro-2′H-spiro [imidazole-4,10′-pyrano­[3,2-*b*]­chromen]-8′-yl trifluoromethanesulfonate **57**, followed by its Suzuki coupling reaction with fluoropyridin-3-ylboronic acid in the presence of tetrakis­(triphenylphosphine)palladium (0), yielded desired hexahydropyrano­[3,2-*b*] chromene analog **58** in 14% yield (Scheme 8).

**Scheme 8:**
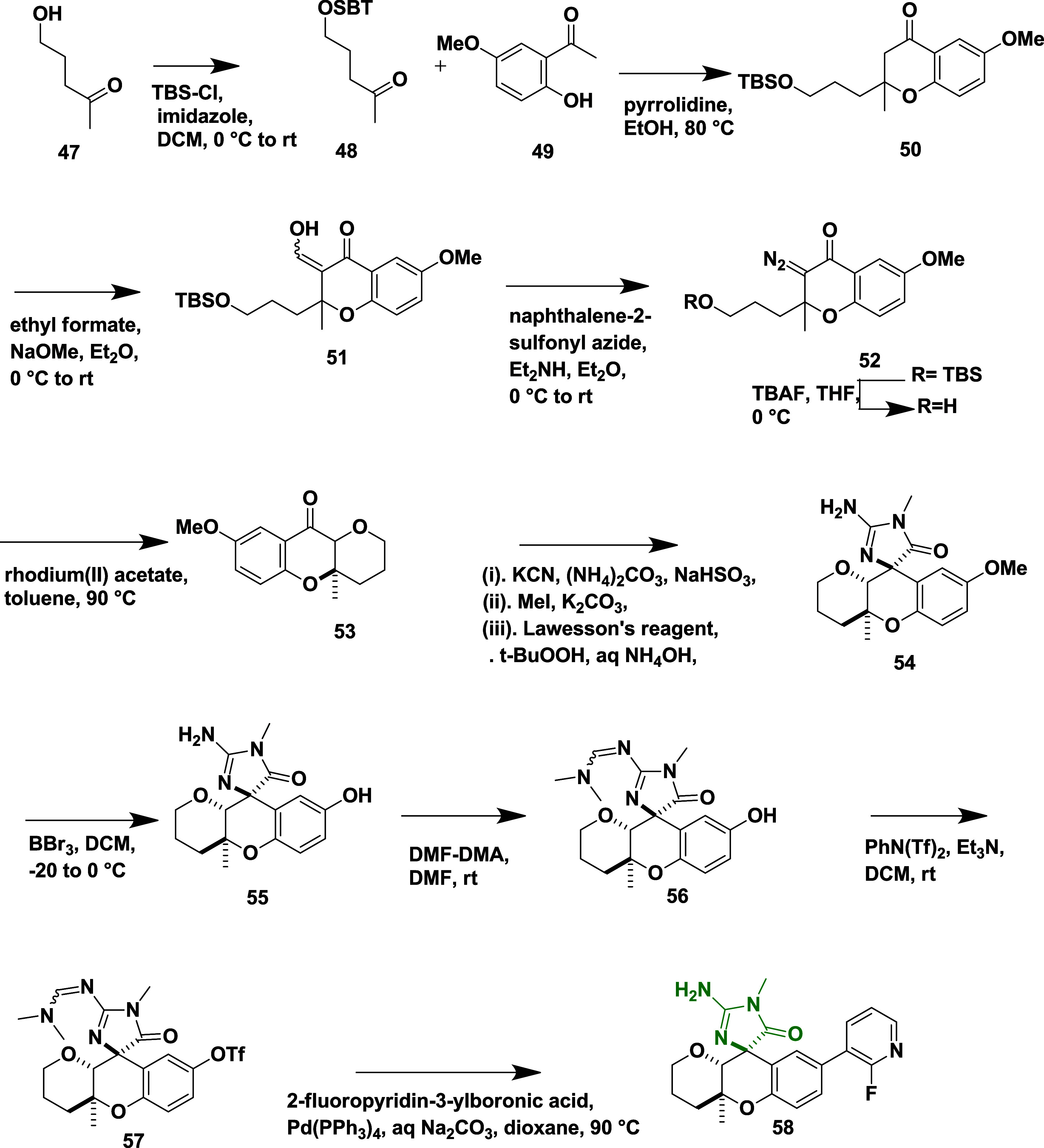
Synthesis of Hexahydropyrano­[3,2-b] Chromene Analog **58**

Tebbe′s reagent is used to introduce a methylene group into (4a*R*,10a*R*)-8-methoxy-4a-methyl-3,4,4a,10a-tetrahydropyrano­[3,2-*b*]­chromen-10­(2H)-one **59** and affords intermediate (4a*R*,10a*S*)-8-methoxy-4a-methyl-10-methylene-2,3,4,4a,10,10a-hexahydropyrano­[3,2-*b*]­chromene **60**. Subsequent reaction of **60** with silver pseudohalide complex (AgXCN, where X = O, S) and iodine led to spirocyclic intermediate **61**. The phenol group of **61** was converted to triflates using phenyl trifluoromethanesulfonate in the presence of an organic base (TEA), followed by Suzuki coupling reaction with various boronic acids, which led to the formation of target compounds **62** with a yield in the range of 11–34% (Scheme 9). Compound **62** (X = O, R = 3-cyanophenyl) (Table 1) exhibited potent BACE-1 inhibition, with an IC_50_ value of 45 nM and high selectivity over cathepsin D. Key SAR findings revealed that 8-tetrahydropyran (8-THP) chroman analogs incorporating three different Asp-binding warheads such as acyl guanidines, aminooxazolines, and aminothiazolines, were systematically evaluated to optimize potency, selectivity, and brain permeability. Introduction of a β-methyl group on the 8-THP chroman scaffold significantly enhanced BACE-1 potency and improved selectivity over cathepsin D (>2000-fold), likely due to strengthened interactions with Tyr132 in the BACE-1 active site. Among the warheads, aminooxazolines and aminothiazolines demonstrated superior CNS properties and lower P-glycoprotein-mediated efflux than acyl guanidines. Further optimization at the P3 position led to the development of compound 62, which achieved robust brain exposure and resulted in a 69% reduction in CSF Aβ_1–40_ levels in rat models. These findings underscore the potential of 8-THP chromans as a promising and tunable scaffold for the design of CNS-penetrant BACE-1 inhibitors.

**Scheme 9:**
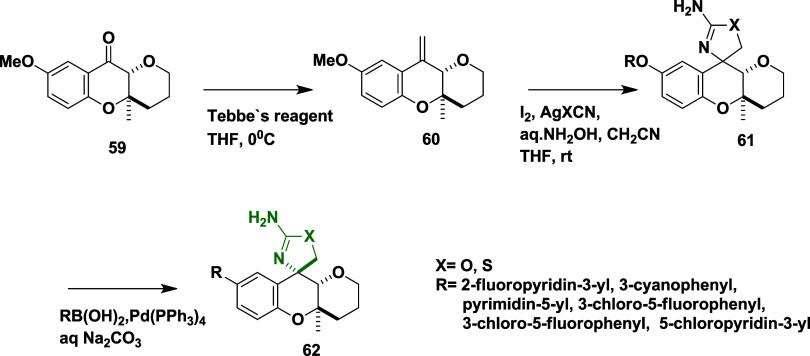
Synthesis of β-Methyl-8-THP Chromans **62**

Egbertson et al. reported the synthesis of a new class of iminohydantoins by introducing a methyl group to an iminohydantoin spiropiperidine.^26^ Which involved the Ugi multicomponent reaction of 2-Methyl-1-[[3-(1-methyl ethoxy)­phenyl]­methyl]-4-piperidine 1,3,8-Triazaspiro[4.5]­dec-3-ene-2-thione **63**, 3-fluoroaniline hydrochloric acid and (5*R*,7*S*)-4-(cyclohexylamino)-1-(3-fluorophenyl)-8-(3-isopropoxybenzyl)-7-methyl-1,3,8-triazaspiro­[4.5]­dec-3-ene-2-thione **64**. The thio-ugi adduct **64** then reacts with thiocarbonyl chloride by converting the thiocarbonyl into the imino thiochloride, which subsequently transforms into the imino ether. Followed by hydrolysis with acetic acid and methanol yielded (5*R*,7*S*)-4-(cyclohexylamino)-1-(3-fluorophenyl)-8-(3-isopropoxybenzyl)-7-methyl-1,3,8-triazaspiro­[4.5]­dec-3-en-2-one **65** with very low yield in the range of 25–45% (Scheme 10). The inhibitor **65** (Table 1) showed good inhibition with an IC_50_ value of 6 nM due to the magic methyl effect, i.e., adding a methyl substituent improved the inhibitor potency by up to 200-fold, primarily by stabilizing the bioactive chair conformation and improving lipophilic interactions with the S1 pocket. Further refinement targeting the S3 pockets, such as introducing an isopropoxy group, enhanced cellular potency and overall drug-like characteristics, without compromising brain penetration or selectivity over cathepsin D. Among the compounds developed, compound **65** emerged as the most effective, exhibiting nanomolar BACE-1 inhibitory activity and producing a significant reduction in CSF Aβ levels in rhesus monkeys. Despite the enhanced potency, these compounds faced challenges related to limited brain penetration.

**Scheme 10:**
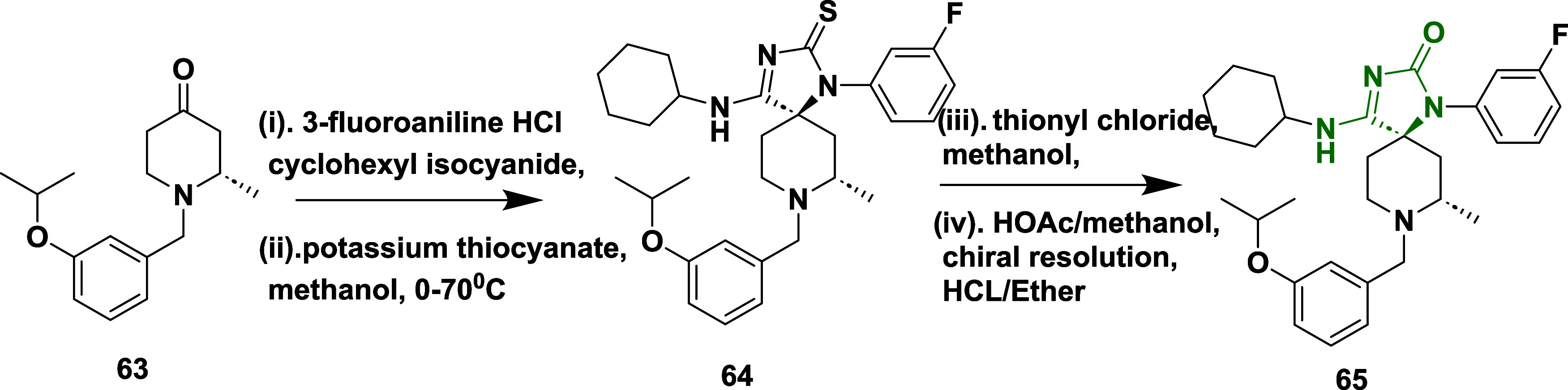
Synthesis of Spiro Piperidine Substituted Imino Hydantoins **65**

Fan et al. reported the synthesis of aminohydontoins as a BACE-1 inhibitor through reactions like Sonogashira coupling, oxidation, and Suzuki coupling.^30^ First, the synthesis of 1-bromo-3-(phenylethynyl) benzene **68** via Sonogashira coupling of 1-bromo-3-iodobenzene **66** with ethynylbenzene **67** in the presence of tetrakis­(triphenylphosphine)­palladium (0), copper­(I)­Iodide, and triphenylphosphine in TEA. The resulting compound was oxidized using a bis­(triphenylphosphine)­palladium­(II) chloride, yielding an intermediate **69**. The Suzuki coupling of **69** with various aryl boronic acids afforded corresponding disubstituted diketones **70**. Finally, the treatment of diketones **69** and **70** with *N*-methyl guanidine hydrochloride in the presence of base TEA at 80 °C produced desired aminohydontoins **71** and **72** with 64–74% yield (Scheme 11). These series achieve good lipophilicity and high inhibitory activity. To effectively engage the S3 pocket on the aminohydantoin scaffold, SAR studies showed that unsubstituted phenyl rings had limited activity unless functionalized with groups like fluorine or methoxy, especially at the 2- or 4-positions. With an IC_50_ of 0.12 μM, compound **69** (Table 1), which contains a 3,4-dimethoxyphenyl group, demonstrated the highest potency among the derivatives and significantly outperformed the parent molecule. Phenyl rings containing polar substituents generally improved target binding by facilitating hydrogen bonding and hydrophobic interactions within the S3 pocket, preserving physicochemical properties like logP and tPSA, essential for CNS penetration.

**Scheme 11:**
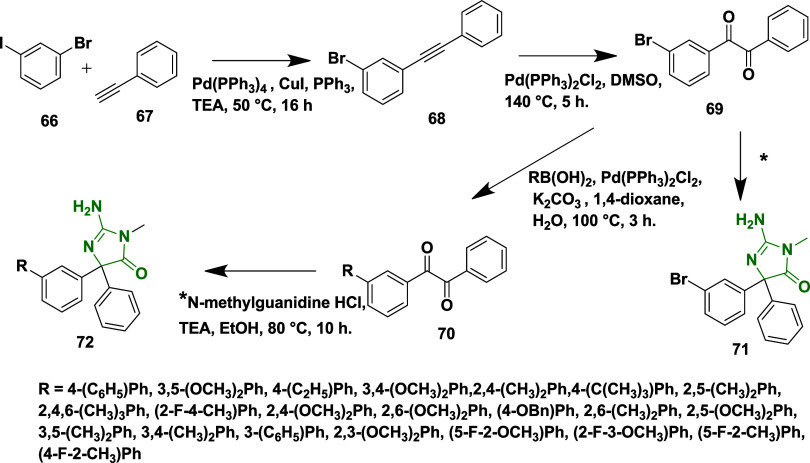
Synthesis of Aminohydontoins **71** and **72**

## Aminooxazoline and Aminooxazines as BACE-1 Inhibitors

Aminooxazoline and aminooxazines are nitrogen and oxygen-containing heterocycles with five and six-membered rings, respectively. These scaffolds have been explored as inhibitors of β-secretase for their potent activity, favorable pharmacokinetic profile­(properties), and improved cell permeability.^31–33^

Huang et al. synthesized a novel aminooxazoline xanthenes as a β-secretase inhibitor.^31^ Biaryl aminooxazoline **77**, synthesized from ketone **73**, underwent boron tribromide-mediated demethylation, followed by difluoromethylation, resulting in an intermediate, and subsequently underwent a Wittig reaction to produce an intermediate **74**. The reaction of **74** with silver isocyanate and iodine afforded **75**, which further cyclizes to produce penultimate aminooxazoline bromide **76**, which then undergoes a Suzuki coupling reaction, yielding the desired biaryl aminooxazoline derivative **77** in 57% yield (Scheme 12).

**Scheme 12:**
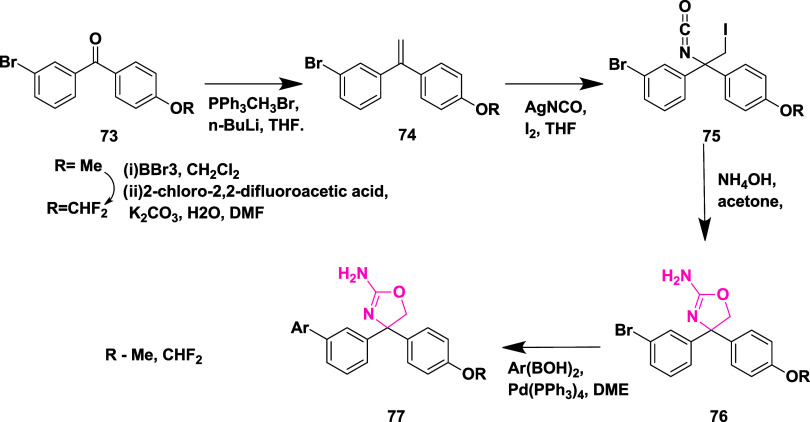
Synthesis of Biaryl Aminooxazoline Derivatives **77**

Aminooxazoline xanthenes **82**, synthesized from aryl bromide **78**. The Cu-catalyzed etherification of **78** yielded diaryl ether **79**, followed by Friedel-Craft acylation to yield Xanthenone **80** (R = O), which was then converted to olefin **80** (R = CH_2_) by a Peterson-type olefination. Subsequently, the reaction of an olefin with ammonium hydroxide after adding in situ-created iodine isocyanate yielded 2-aminooxazoline aryl bromide **81**. The Suzuki coupling reaction of **81** with various aryl boronic acids afforded 2-aminoxazolinexanthene **82** (Scheme 13). Further modification involved demethylation of **83** using boron trifluoride, yielding phenol **84**, followed by a Suzuki coupling reaction to give intermediate **85**. The alkylation of **85** with alkyl iodide afforded alkoxy xanthenes **86**, followed by chiral separation, yielding pure enantiomer **87** (Scheme 14), with yields ranging from 47–99%. The inhibitor **87**(if R = i-Bu) (Table 2) showed good BACE-1 inhibition by significantly reducing CNS Aβ40 with an IC_50_ value of 8 nM. SAR investigations on aminooxazoline xanthene derivatives revealed that introducing meta-substituents and incorporating hydrophobic groups near the S2′ pocket led to notable gains in BACE-1 potency and selectivity over cathepsin D. Meta-substituted aryl moieties, such as 2-fluoro-5-chlorophenyl, along with heteroaromatic rings like pyrimidine, promoted favorable interactions within the S1/S3 pockets and aided in achieving CNS penetration. Optimized candidates, including compound **87**, demonstrated low P-gp efflux, high microsomal stability, and significant in vivo activity by lowering Aβ_40_ concentrations in both the CSF and brain of rats following a single oral dose. Structural analysis through X-ray cocrystallography confirmed critical hydrogen bonds and hydrophobic contacts responsible for the enhanced biological profile. These inhibitors are CNS penetrable and metabolically stable but have limited oral bioavailability for selected inhibitors.

**Scheme 13:**
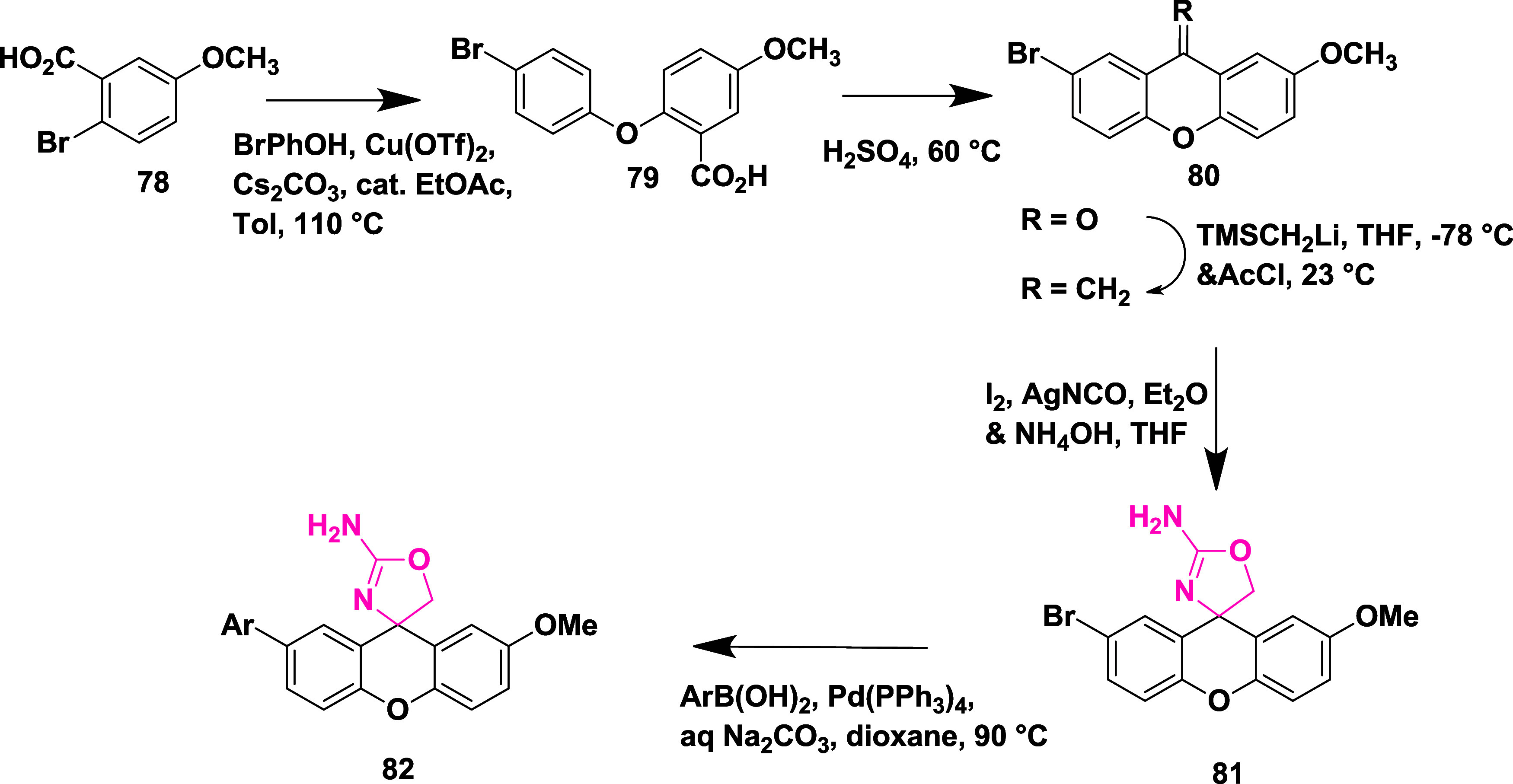
Synthesis of 2-Amino Oxazoline Xanthene **82**

**Scheme 14:**
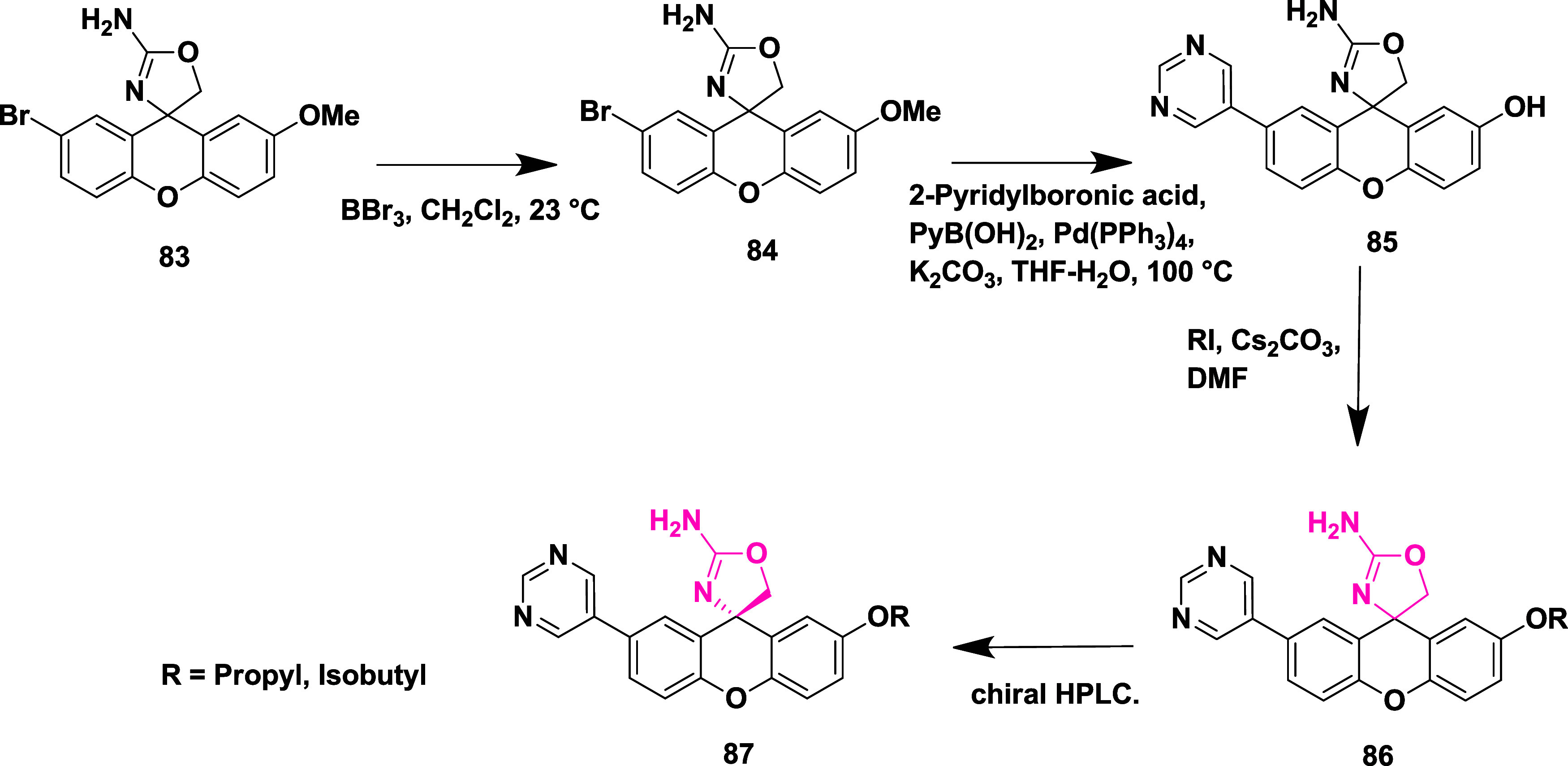
Synthesis of Pyrimidine-substituted 2-Amino-oxazoline Xanthene **87**

**Table 2:**
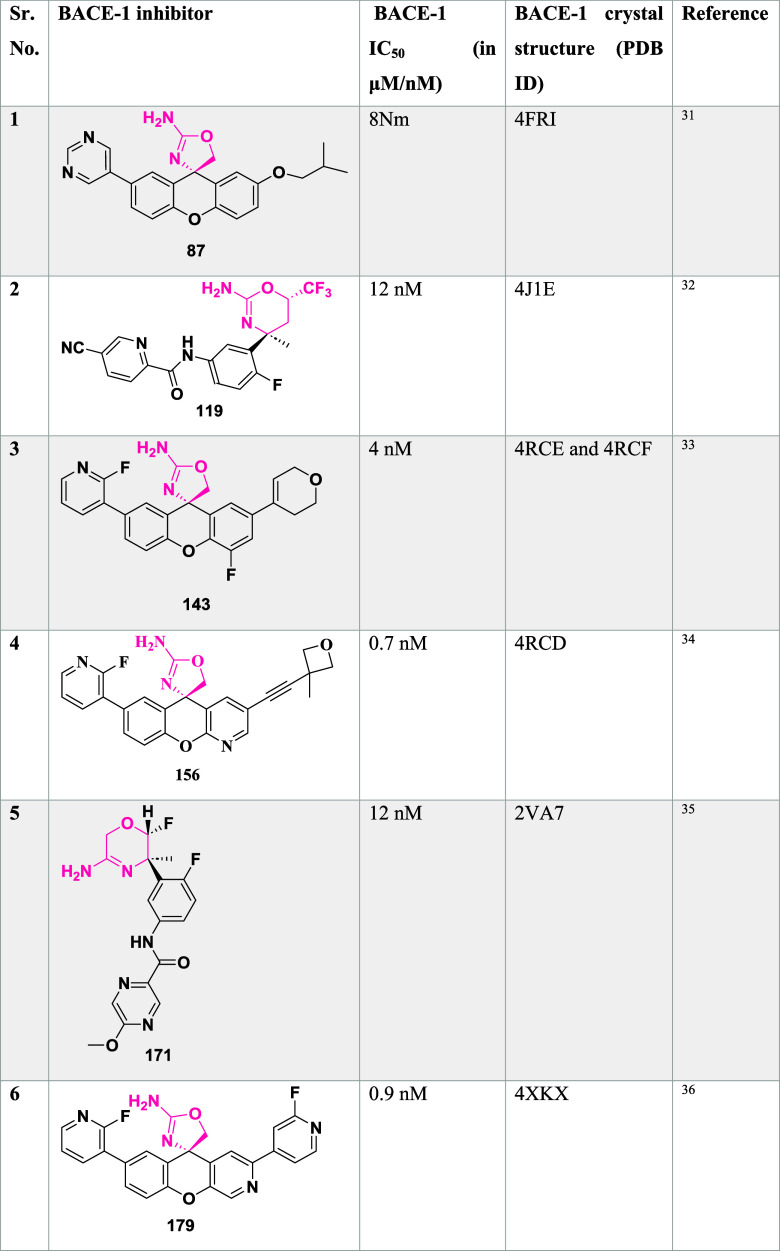
BACE-1 Inhibitors, the Co-Crystal Structure of BACE-1 Enzyme, and Their IC_50_ Values

Hilpert et al. reported the synthesis of β-secretase inhibitors with high in vivo efficacy suitable for clinical evaluation in AD.^32^ The condensation of acetophenone **88** with (*R*)-*tert*-butane sulfonamide **89** yielded sulfonyl imine **90**. Subsequent transmetalation of lithium enolate using a chlorotriisopropoxytitanium­(IV) (TiCl­(OiPr)_3_) to produce titanium enolate, which reacted with **90** to yield ester **91**(R = H). Alternatively, **90** underwent Reformatsky reaction with ethyl bromide fluoroacetate to produce α, α-difluoro esters **91** (R = F). Lithium borohydride catalyzed the reduction of ester **91** to corresponding alcohol **92**, followed by acid cleavage of the chiral auxiliary to produce amino alcohol **93**. Cyclization of **93** with cyanogen bromide yielded oxazines **94**, which were reduced to aniline **95**. Finally, the target compound, **96**, was obtained through amide formation (Scheme 15). In a parallel sequence, sulfonyl amine **99** underwent a Reformatsky reaction, affording esters **100**. As mentioned in Scheme 15, oxazine **101** was prepared and nitrated with fuming nitric acid. Following nitro group reduction and amide formation, the desired fluorinated oxazines **106** were formed (Scheme 16).

**Scheme 15:**
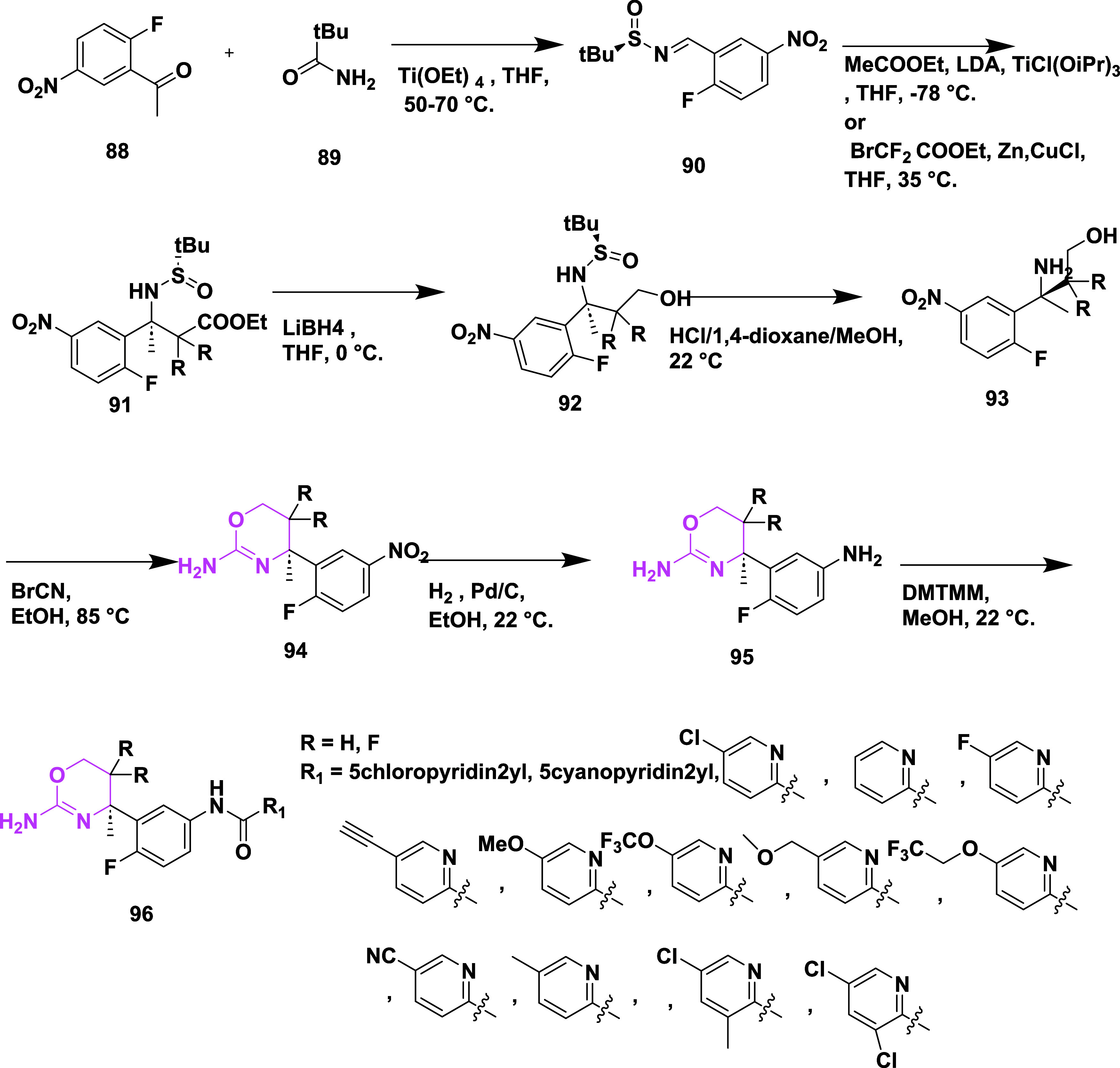
Synthesis of *N*-(3-((4*R*)-2-Amino-5-fluoro-4-methyl-5,6-dihydro-4*H*-1,3-oxazin-4-yl)-4-fluorophenyl) Acetamide Derivatives **96**

**Scheme 16:**
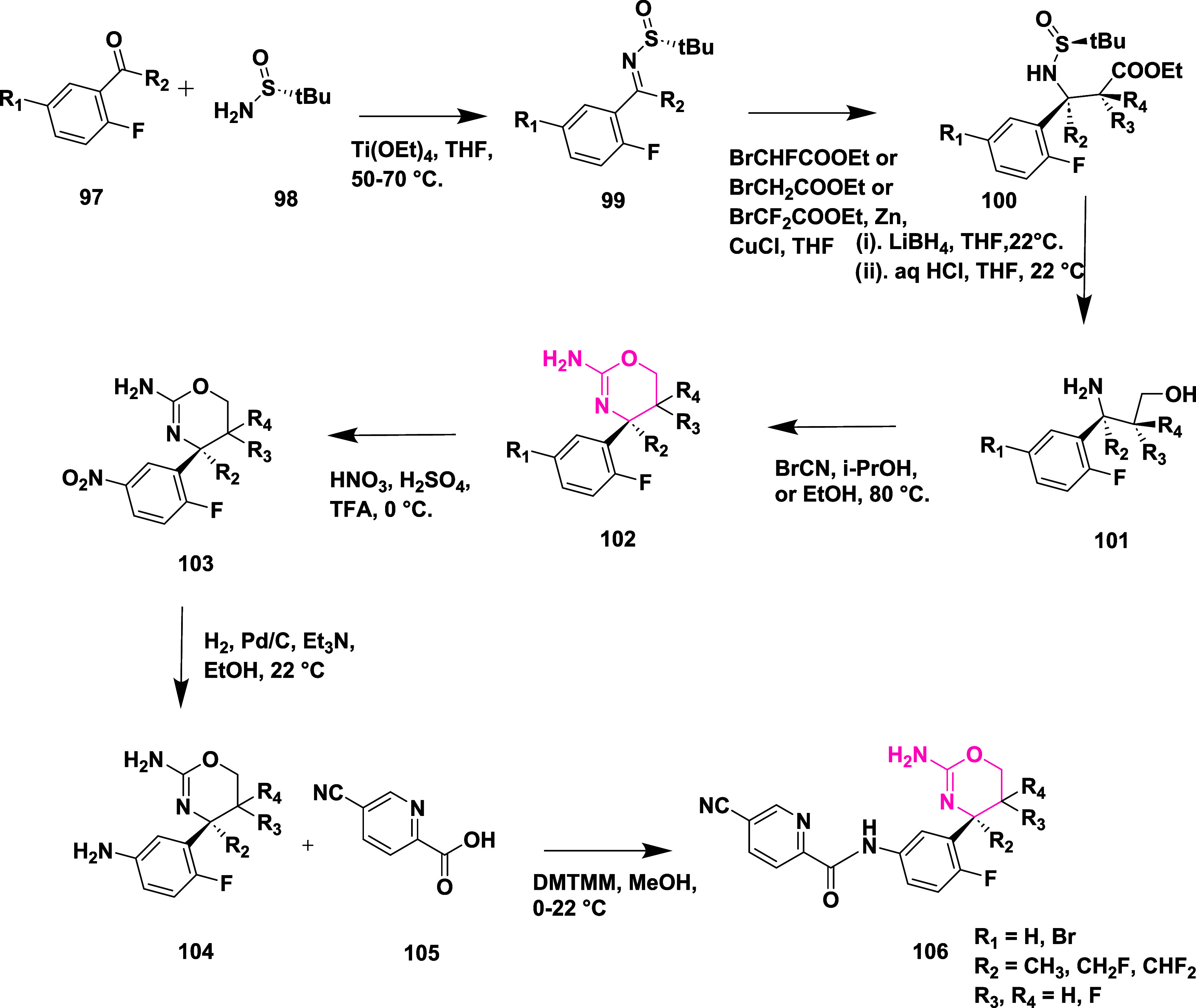
Synthesis of 5-Cyano-*N*-(4-fluorophenyl) Picolinamide Substituted Oxazine Derivatives **106**

A different synthetic strategy was employed to synthesize trifluoromethyl-substituted oxazines **119** (Scheme 17). The synthesis begins with hydroxyamino ester **107**, which reacts with trifluoropropene **108** in the presence of an inorganic base at low temperature in an ethyl acetate medium, producing in situ nitrile oxide, which then undergoes 1,3-dipolar cycloaddition, yielding isooxazoline ester **109**. Subsequently, the reduction of esters to the corresponding alcohol in the presence of sodium borohydride and then fluorinated with deoxo-fluor at −78 to 22 °C to yield fluorinated isooxazoline **110** in dichloromethane. The compound **111** was synthesized by reacting **110** with phenyl isocyanate and nitroethane to produce imine, followed by arylation with 1-bromo-2-fluorobenzene **112** using *n*-butyllithium and BF_3_·Et_2_O, which afforded **113**. Subsequently, a ring opening of **113** in the presence of ammonium formate and palladium–carbon yielded the corresponding amino alcohol **114**. The amino alcohol was converted to aminooxazoline derivative **115** by reacting with cyanobromine, followed by nitration, catalytic reduction to afford anilines **117**. This aniline oxazine **117** reacted with isatin-derived compound **118** in the presence of coupling reagent DMT-MM to yield the final trimethylated oxazine derivative **119** (69%) (Scheme 17). A different synthetic approach was required to synthesize the final trifluoromethyl-substituted oxazines **129** (Scheme 18). Diisobutylaluminum hydride (DIBAL-H) reduced esters **121** at −76 °C and produced aldehydes **122**; after reacting with trimethyl­(trifluoromethyl)­silane, these aldehydes formed alcohols **124**. Alcohols **124** were converted to target compounds **129** using pre-established protocols described in earlier Scheme 17. Compounds were isolated with a yield of 41–69%. The uniqueness of this series was that the fluorination decreased the acid dissociation constant, thereby improving the pharmacological profile of the inhibitors. SAR studies showed that introducing fluorine into 1,3-oxazine derivatives effectively reduced the p*K*_a_ of the headgroup, which enhanced brain penetration, BACE-1 inhibitory activity, and in vivo performance. Incorporating electron-withdrawing groups such as nitrile, fluorine, or trifluoromethyl on the aromatic ring and oxazine scaffold improved binding within the S3 pocket and boosted metabolic stability and selectivity against BACE-2. Among the series, compound **119** (R = H) (Table 2) stood out with strong brain availability and a 78% decrease in Aβ_40_ levels at a 10 mg/kg dose in mice, showing IC_50_ values of 12 nM for hBACE-1 and 2 nM in cellular assays. Structural insights from X-ray crystallography and computational modeling revealed that fluorination promoted conformational rigidity and reduced desolvation energy, contributing to stronger target affinity and improved pharmacokinetics.

**Scheme 17:**
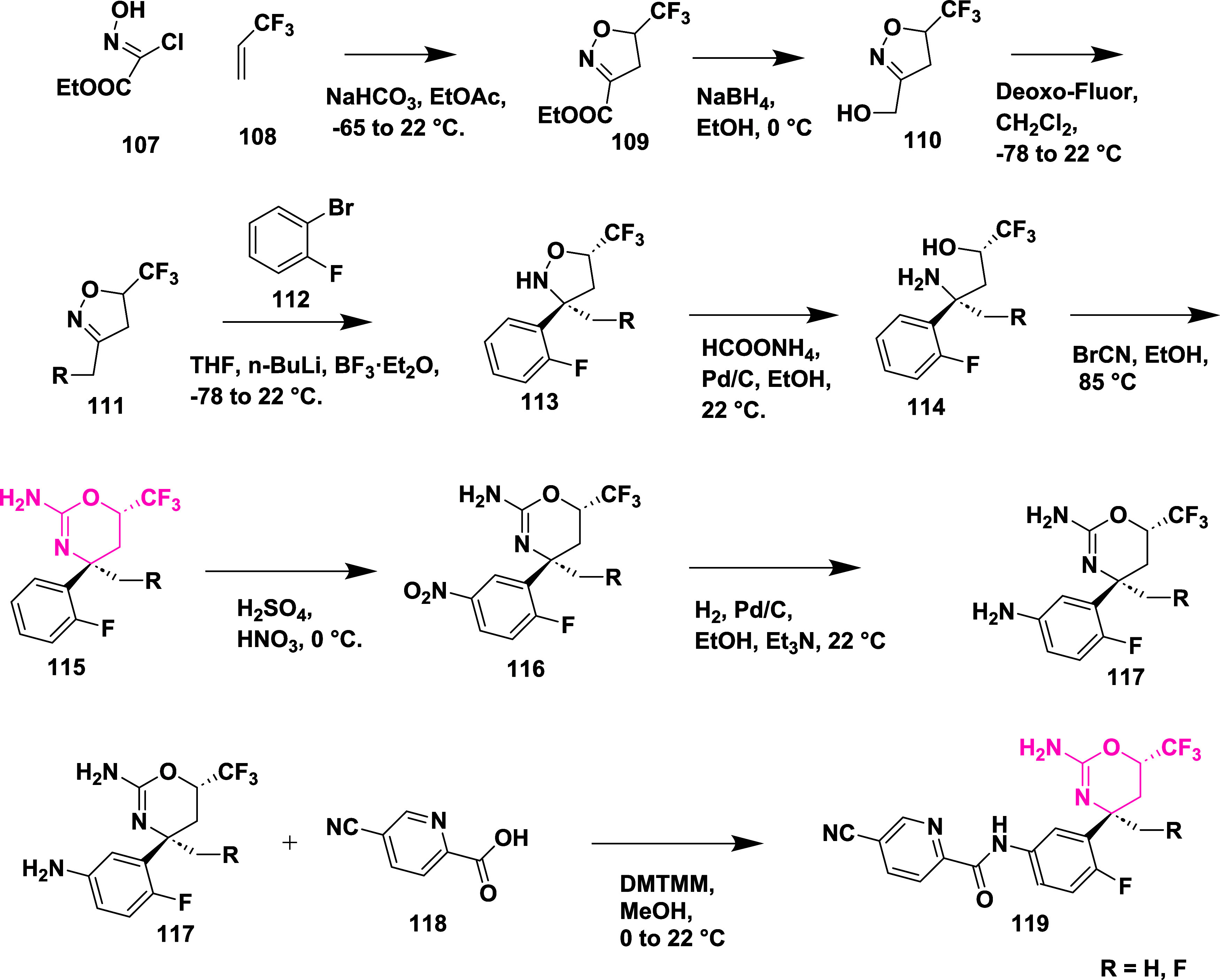
Synthesis of Trifluoromethyl-substituted Oxazine Derivatives **119**

**Scheme 18:**
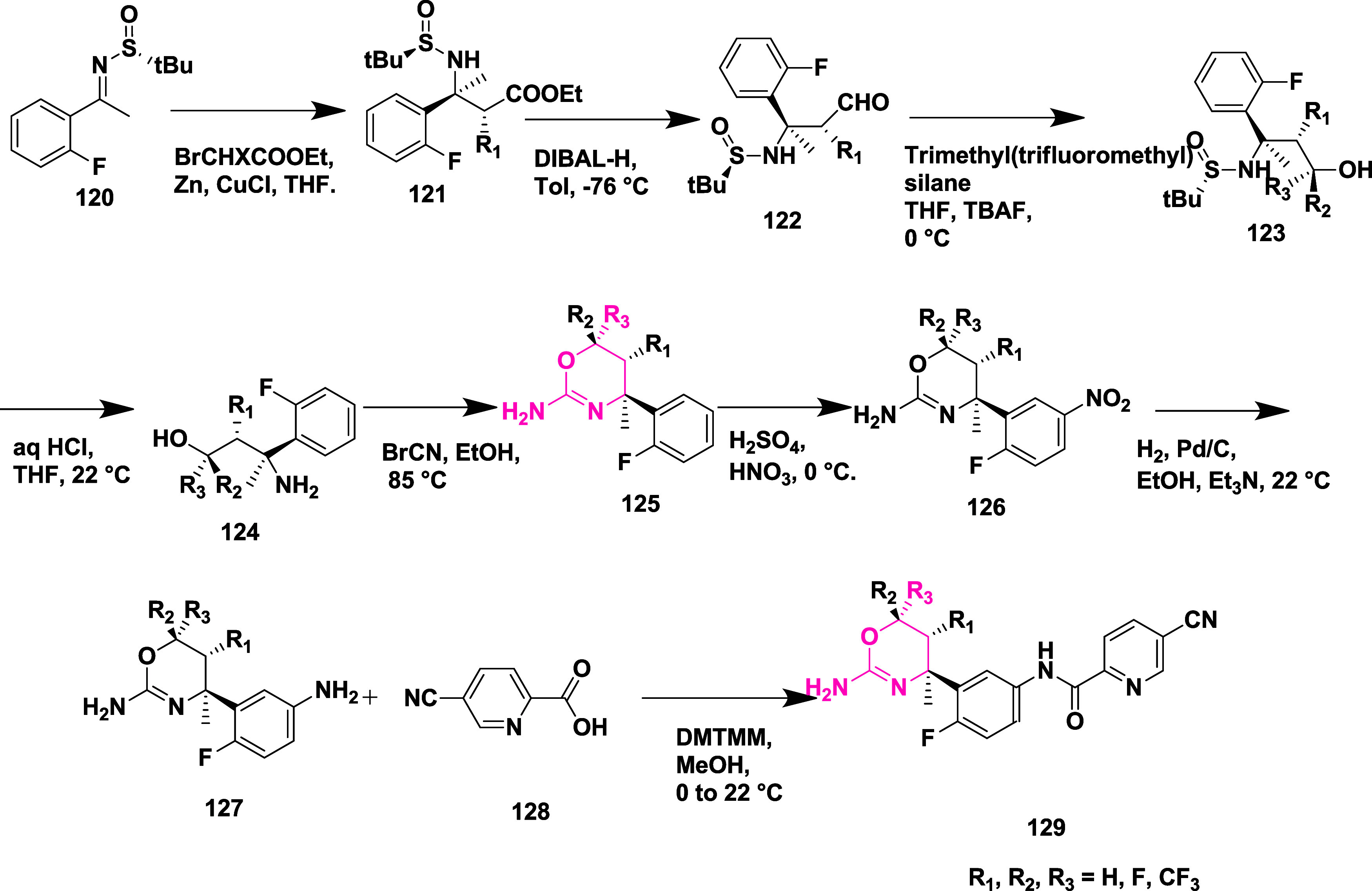
Synthesis of Oxazine Derivatives **129**

Epstein et al. synthesized a series of aminooxazoline xanthene as BACE-1 inhibitors,^33^ through Suzuki coupling reaction of 2′-bromo-7′-chloro-5*H*-spiro­[oxazole-4,9′-xanthen]-2-amine **130** with 5-pyrimidylboronic acid, resulting in an intermediate 2′-chloro-7′-(pyrimidin-5-yl)-5*H*-spiro­[oxazole-4,9′-xanthen]-2-amine **131**. The selective Suzuki–Miyaura coupling of **131** with *t*-butyl acetylene resulted in (*R*)-2′-(3,3-dimethylbut-1-yn-1-yl)-7′-(pyrimidin-5-yl)-5*H*-spiro­[oxazole-4,9′-xanthen]-2-amine **132**. Finally, Pd-catalyzed hydrogenation of **132** afforded the desired target compound **133** (Scheme 19).

**Scheme 19:**
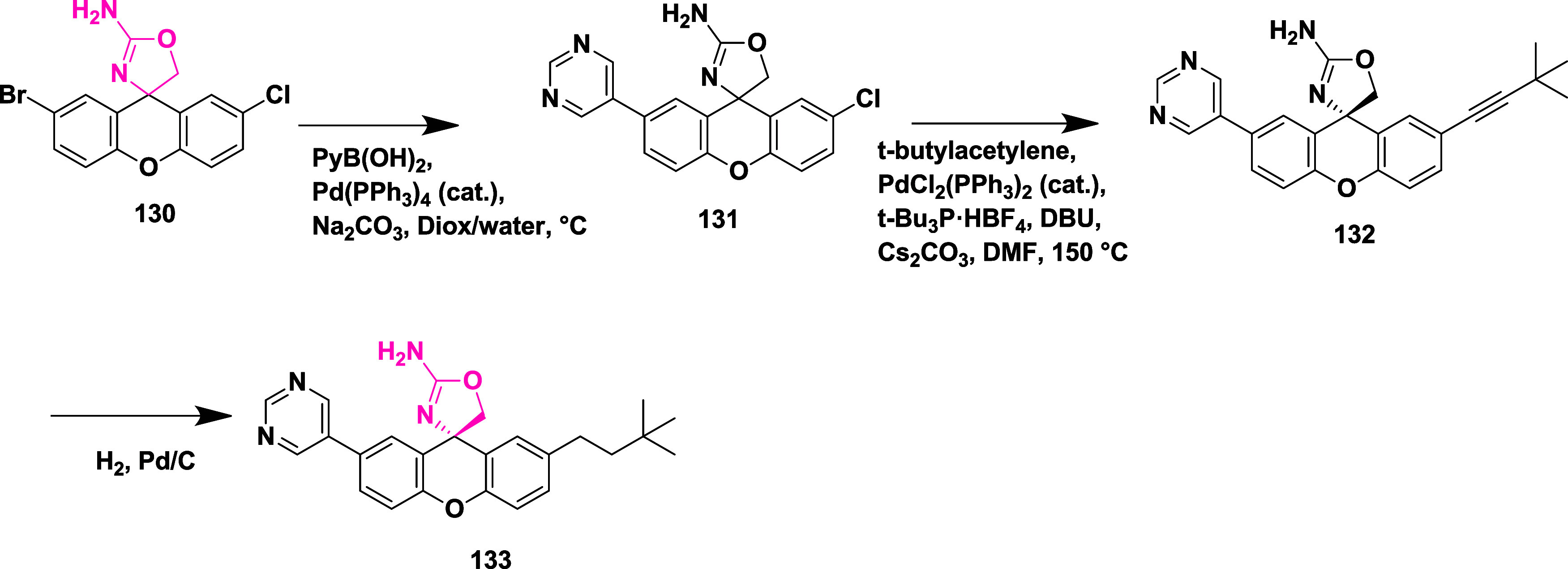
Synthesis of Dimethyl Butyl and Pyrimidinyl Substituted Spirooxazolo Xanthenylamine **133**

The preparation of (*S*)-2′-bromo-7′-(pyrimidin-5-yl)-5*H*-spiro­[oxazole-4,9′-xanthen]-2-amine **135** involved a selective Suzuki coupling reaction of intermediate **134** with 5-pyramidal boronic acid, followed by another Suzuki coupling reaction of **135** with 4-dihydropyran boronate ester, producing the target **136**. The synthesis of the oxygen-linked R substitute from the xanthene core started with 7-bromo-2-methoxy substituted **137**. **137** undergoes demethylation, mediated by boric tribromide, yielding phenol **138**. Therefore, compounds with O-linked R groups **140** were produced by alkylating the phenol and adding a 5-pyrimidyl R group. Heteroaryl groups were added to the aryl bromide **141** by Pd-catalyzed coupling with commercially available boronic acids/boronate from esters or organo tin compounds, yielding **142** (Scheme 20). Structure–activity relationship (SAR) studies of aminooxazoline xanthene compounds aimed to improve BACE-1 inhibitory activity while addressing challenges related to P-glycoprotein (P-gp) efflux and hERG inhibition. Introducing polar substituents onto the xanthene scaffold lowered hERG-related risk but tended to increase P-gp efflux, unless counterbalanced by nearby lipophilic groups. Combining a 2-fluoro-3-pyridyl substituent with a 4-fluoro group on the xanthene ring enhanced BACE-1 binding affinity through favorable interactions with residues such as Trp76 and Ser229. Among the tested derivatives, Compound **143**, synthesized following a procedure similar to that outlined in Scheme 20, emerged as a lead, showing potent BACE-1 inhibition (IC_50_ = 4 nM) (Table 2), robust Aβ reduction in rat brain (67%) and CSF (78%), and no significant cardiovascular toxicity in dog models.

**Scheme 20:**
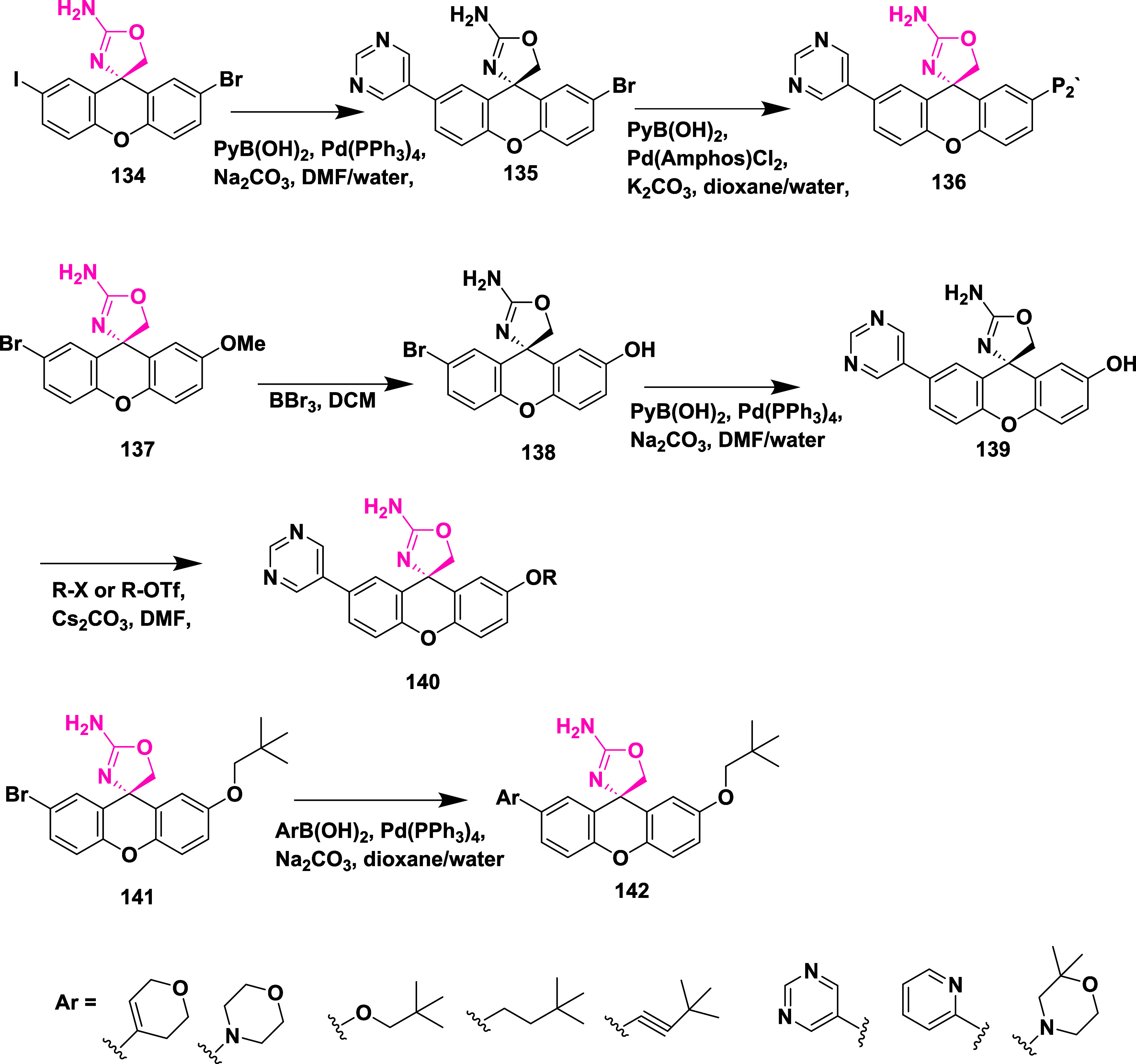
Synthesis of Oxazoline Derivatives **136**, **140**, and **142**

Dineen et al. reported the synthesis of a new BACE-1 inhibitor of AD.^34^ Synthesis of the target molecule begins with coupling 2-fluoropyridine **145** with *p*-bromophenol **144** in DMF under basic conditions, followed by ring closure in neat polyphosphoric acid to yield xanthenone **146**. Ketone **146** underwent hydrochloric acid treatment and a reaction with methyl magnesium chloride to form an unstable exo-olefin. Treating this olefin with an in situ-generated iodine isocyanate and an ammonia solution in 2-propanol afforded the racemic 2-amino oxazoline xanthene **147**. The (*S*)-enantiomer **147** was obtained on a multigram scale by chiral supercritical fluid chromatography separation of the racemic mixture. Neopentyl ether **149** was prepared by demethylating compound **147** via boron tribromide and then alkylating intermediate phenol **148** with neopentyl iodide by promoting cesium carbonate. The palladium-catalyzed Suzuki cross-coupling reactions with the corresponding boronic acids yield the desired target molecule **150** with a 70–90% (Scheme 21).

**Scheme 21:**
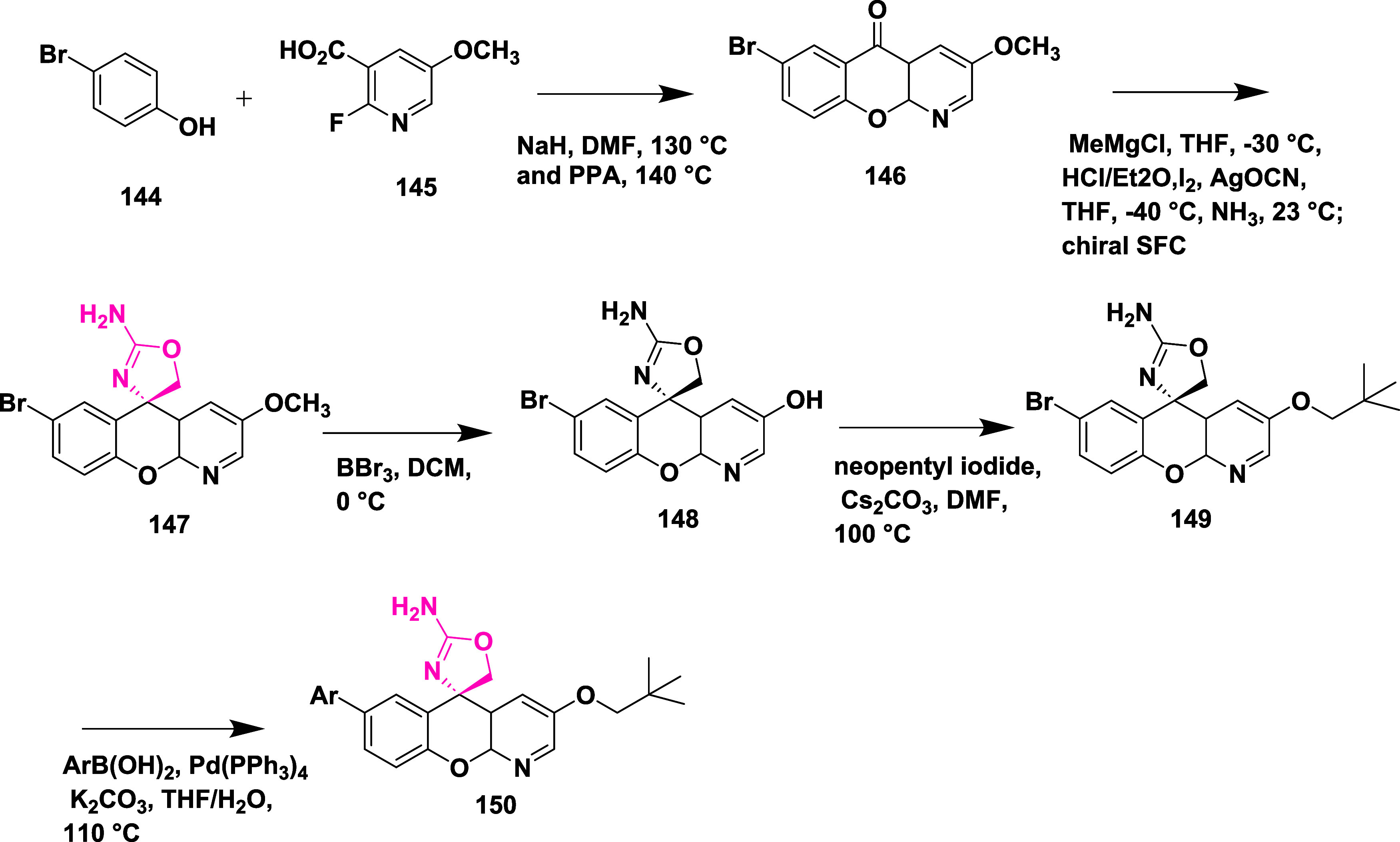
Synthesis of Neopentyloxy and Spirochromenopyridine Substituted Oxazolyl Amine **150**

Xanthenone **153** was synthesized by the reaction of 2-chloropyridine **152** and *p*-iodophenol **151**, followed by acidic ring closure, chiral separation, and 2-amino oxazoline xanthene unit formation, resulting in the bis-functionalized iodo-bromo intermediate **154**. **154**, then converted to **155** by coupling of **154** with 4-Fluoro-2-pyridylboronic acid. The bromide **155**, subjected to a Sonogashira cross-coupling reaction to generate the desired compounds **156**, was produced by selectively coupling the aryl iodide with 2-fluoro-3-pyridine boronic acid (Scheme 22). The isolated compound yielded in the range of 40 to 93%. SAR studies showed that introducing a nitrogen atom at the 4-position of the xanthene core strengthened interactions with Trp76 and reduced hERG liability. Substitution at the P3 site with a 2-fluoro-3-pyridyl group enhanced binding affinity through favorable contacts with Ser229 and preserved brain permeability. Modifications at the P2′ position, notably the addition of an oxetane-linked alkyne, further improved pharmacokinetic properties and reduced efflux. These collective optimizations led to the development of compound **156** (AMG-8718) (R = 3-methyl-3-(prop-1-yn-1-yl)­oxetane) (Table 2), which exhibited potent BACE-1 inhibition with an IC_50_ value of 0.7 nM, significant in vivo Aβ-lowering effects, and a favorable CNS and safety profile.

**Scheme 22:**
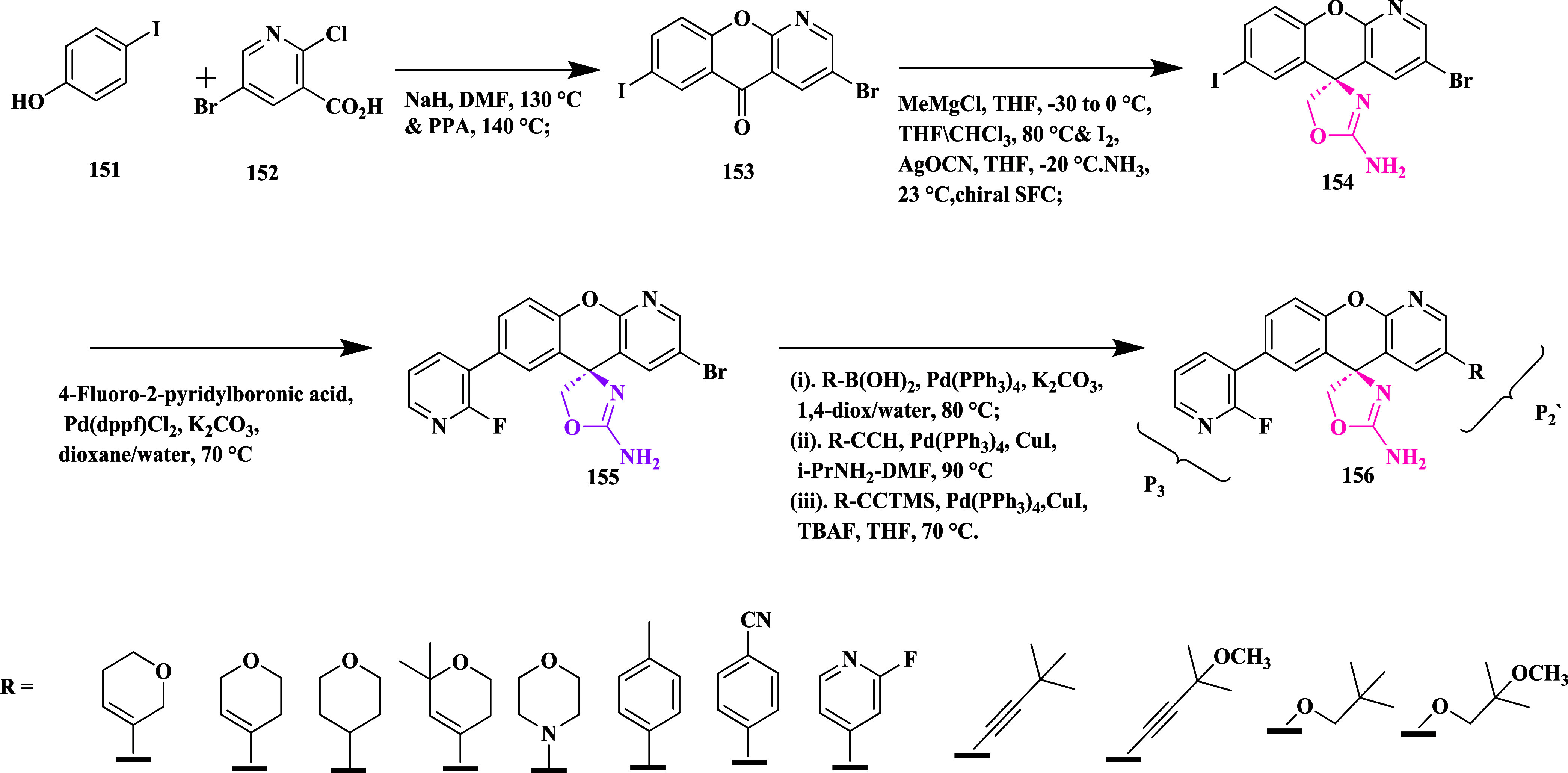
Synthesis of 2-Fluoropyridine Substituted Aminooxazoline Derivative **156**

Rombouts et al. reported the synthesis of novel 1,4-oxazine derivatives as AD’s BACE-1 inhibitors.^35^ The synthesis of 1,4-oxazine derivative **166** began with acetophenone **157**, which underwent a Strecker reaction to afford aminonitrile intermediate **158**, followed by acid hydrolysis and esterification using sulfuric acid to yield **160**. The racemic amino alcohol **161** was formed from **160** by reduction of **160** using lithium aluminum hydride, which was then subsequently resolved into its chiral enantiomers **162**. Lactam ring **163** formed via a one-pot, two-step reaction of **162** with chloroacetyl chloride in the presence of DIPEA and the potassium salt of tertiary butoxide, followed by the formation of amidine derivative **164** through thionation and aminolysis. Bromoarene undergoes Buchwald-type amination using benzophenone imine to produce an intermediate **165**, followed by 4-(4,6-dimethoxy-1,3,5-triazin-2-yl)-4-methylmorpholinium chloride-mediated coupling with reaction acid to yield **166** (Scheme 23). In Scheme 24, the diastereoisomeric mixture **167** was converted into target amidine **171**, using a similar synthesis sequence described in Scheme 23, with the key variation being the conversion of bromoarene into the aniline using a copper-catalyzed reaction with sodium azide, with the yields ranging from 30 to 96%. SAR analysis indicated that electron-withdrawing substituents like fluorine and trifluoromethyl were introduced at the C-2 position of the oxazine ring, effectively lowering the p*K*_a_ of the amidine moiety, which improved brain penetration and reduced efflux. The configuration at the 2*R*,3*R* positions, along with appropriate substitution, proved essential for maintaining biological activity and achieving desirable pharmacokinetic properties. Notably, compound (2*R*,3*R*)-**171** (if R_1_ = H, R_2_ = F) emerged as a lead molecule, combining low p*K*_a_, exhibiting notable β-secretase inhibition activity with an IC_50_ value of 12 nM, efficient CNS delivery, and minimal efflux, highlighting its potential for Alzheimer’s disease treatment.

**Scheme 23:**
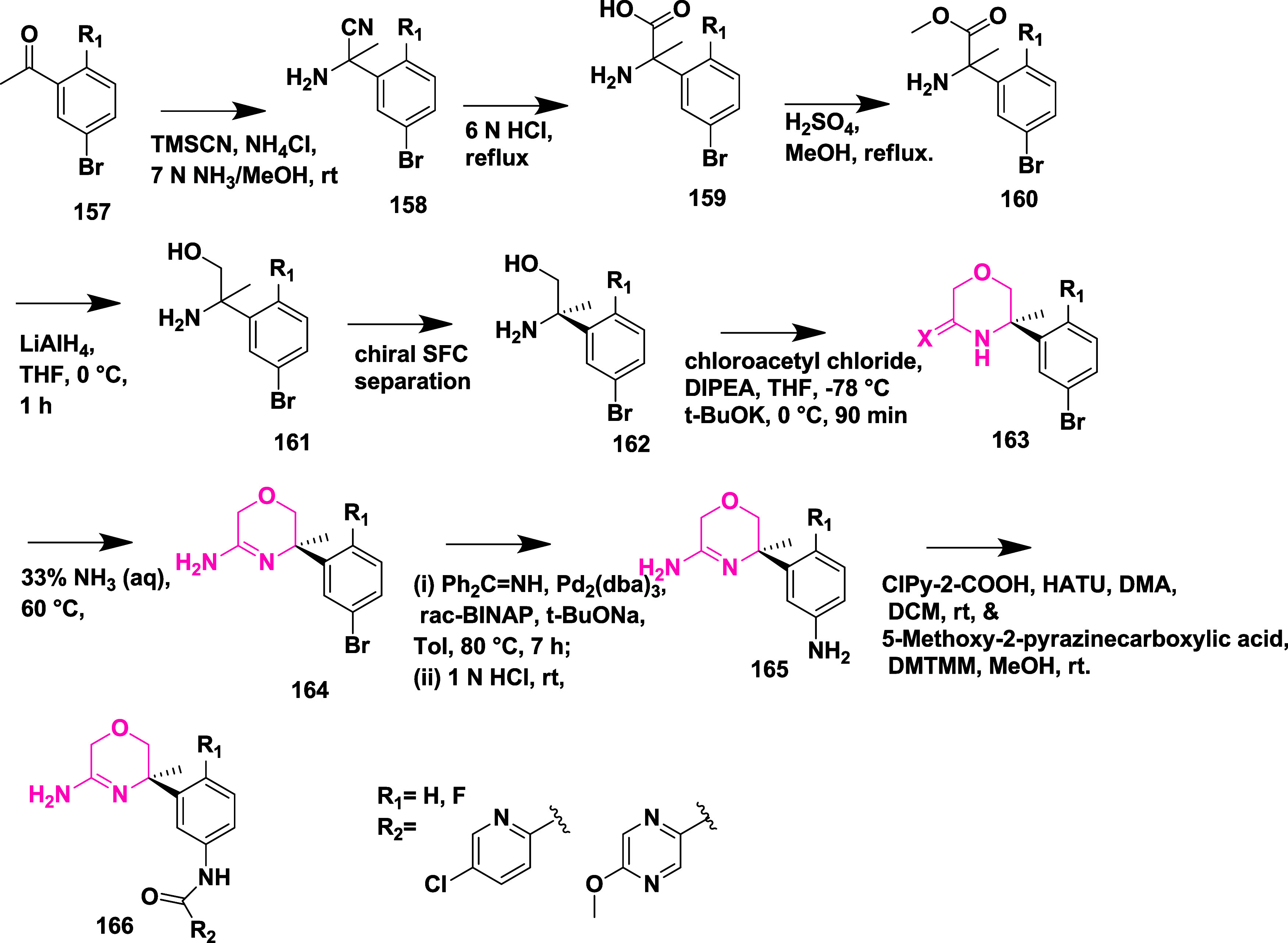
Synthesis of 1,4-Oxazine Derivative **166**

**Scheme 24:**
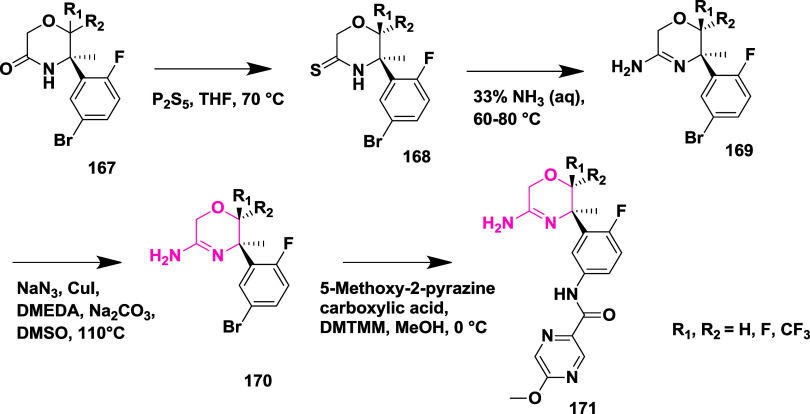
Synthesis of Amide-substituted 1,4-Oxazine Derivative **171**

Jeffrey et al. developed an orally effective 2-aminooxozoline 3-aza xanthene derivative by property and structure-based design as a BACE-1 inhibitor.^36^ The synthesis of this inhibitor involved the copper­(II) triflate-catalyzed coupling of 2,5-dibromobenzoic acid with 6-chloropyridin-3-ol, 172, produced an intermediate acid, which was then coupled with diethylamine to yield the amide intermediate **173**. Lithiation (using a lithium-diisopropylamine) of pyridine, followed by cyclization, results in the desired 3-azaoxanthenone **174**. Grignard addition to the ketone **174** and followed by dehydration results in intermediate 7-bromo-3-chloro-5-methylene-5*H*-chromeno­[2,3-*c*]­pyridine **175**. The racemic 2-aminoxazoline 3-aza xanthene was synthesized from compound **175** and treated with iodine isocyanate and ammonia. Chiral separation results in (*S*)-7-bromo-3-chloro-5′H-spiro­[chromeno­[2,3-*c*]­pyridine-5,4′-oxazol]-2′-amine **176**, which undergoes Suzuki coupling with (2-fluoropyridin-3-yl)­boronic acid **177** to yield an intermediate (*S*)-3-chloro-7-(2-fluoropyridin-3-yl)-5′H-spiro­[chromeno­[2,3-*c*]­pyridine-5,4′-oxazol]-2′-amine **178**. Further, Palladium-catalyzed coupling produced the desired compound **179** with yields within 22 to 93% (Scheme 25). SAR analysis found that incorporating a nitrogen atom at the third position of the xanthene ring proved beneficial, enhancing hydrogen bonding with Trp76 and leading to better metabolic properties and target selectivity. Modifications at the region corresponding to the S2′ pocket (substitutions at the P2′ position) using groups like dihydropyran, morpholine, difluoropiperidine, and fluorinated pyrrolidines were systematically assessed to fine-tune activity, reduce efflux by transport proteins, and maintain favorable pharmacokinetics. Compound **179** (if P2′ = fluoropyridine) (Table 2) was identified as a standout molecule, showing potent BACE-1 inhibition both in vitro and in vivo, minimal P-gp efflux, excellent bioavailability in multiple species, and a promising safety profile. Structural data supported its design, revealing essential binding features such as interactions with the enzyme’s catalytic aspartates and occupation of the S2′ and S3 subsites.

**Scheme 25:**
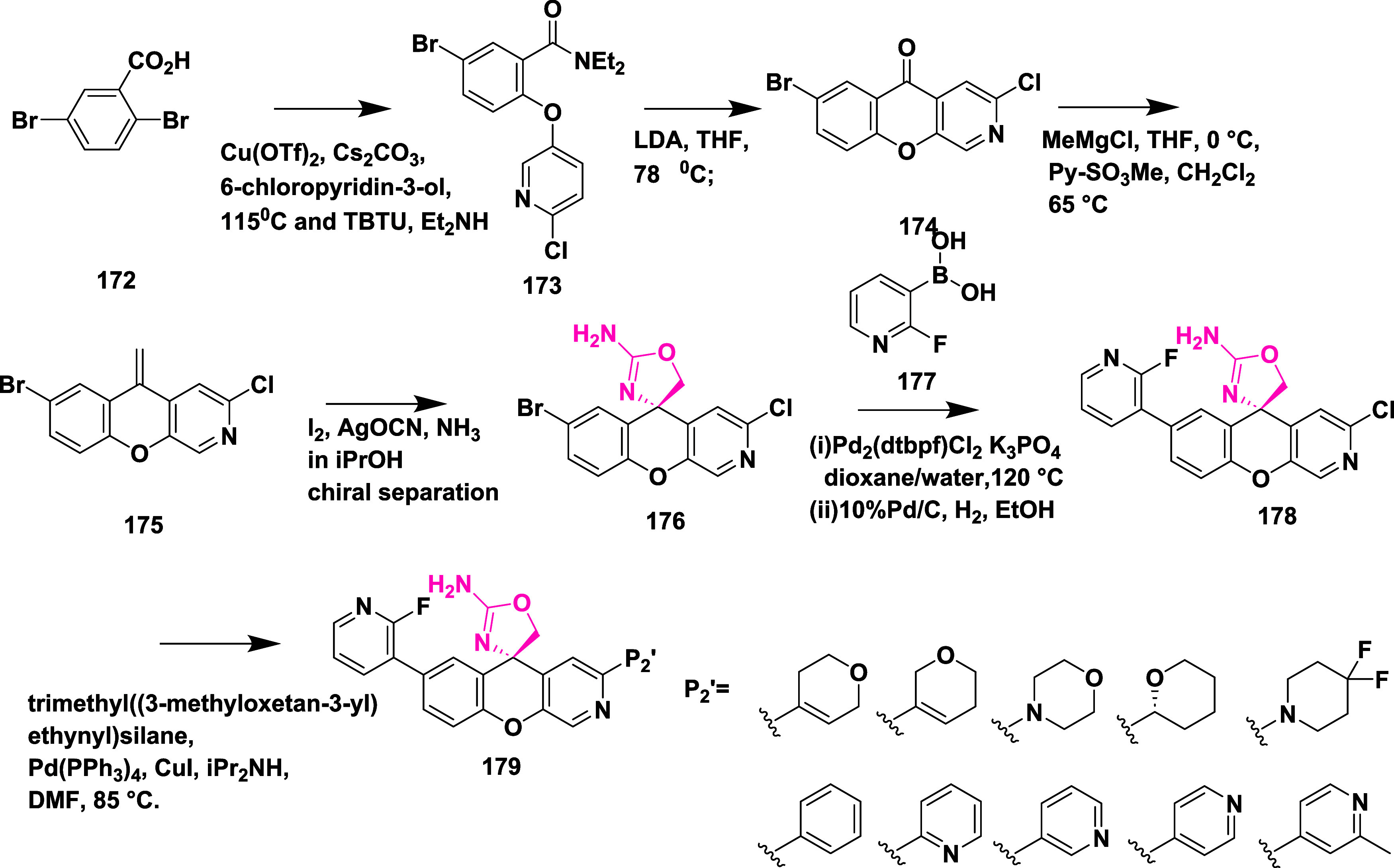
Synthesis of 2-Aminooxazoline 3-Azaxanthenes **179**

## Aminothiazines, Iminothiadiazine Dioxide, and Aminothiazole as BACE-1 Inhibitors

Aminothiazine and iminothiadiazine dioxide are six-membered heterocycles containing nitrogen and sulfur atoms with one amino group (aminothiazine), one imine group, and a sulfonyl group (Iminothiadiazine). In contrast, aminothiazole is a five-membered ring containing a thiazole ring with one amine group. These derivatives are known for their biological activity, such as anticancer, antimalarial, antimicrobial, anti-Alzheimer, etc.^37–41^ The iminothiadiazine dioxide showed improved solution stability, making it an attractive scaffold for drug discovery in medicinal chemistry.

Brodney et al. reported the synthesis of the novel 2-amino-4,5-dihydrothiazole derivatives as a BACE-1 inhibitor^42^ Lithium aluminum hydride-mediated reduction of acetylenic alcohol **180**, which afforded trans-allylic alcohol **181**, followed by oxime formation, resulting in intermediate **182**, which was then converted to the nitrile oxide using bleach. In situ, the cycloaddition of the nitrile oxide resulted in a single isomer of isoxazoline **183**, which was subsequently converted to substituted isoxazolines **186** by treating with methyl or trifluoromethyl substituted isoxazoline with DAST via a continuous flow method. **185** undergoes direct nitrile oxide formation in another synthetic pathway, and subsequent [3 + 2] cycloaddition produces the fused tetrahydropyran ring system **186** as a single stereoisomer. The isoxazolines **187** were formed by anion formation, followed by adding the boron trifluoride-complex of the isoxazoline or halogen–metal exchange at −78 °C in the presence of boron trifluoride–etherate. Zinc in acetic acid reduced the N–O bond of isoxazoline **187**, forming the thioamidine ring. A thiourea intermediate was obtained through a reaction between resistant amino alcohols **188** and benzoyl isothiocyanate. Selective removal of the benzyl ether with boron trichloride yielded the final product **189** (Scheme 26).

**Scheme 26:**
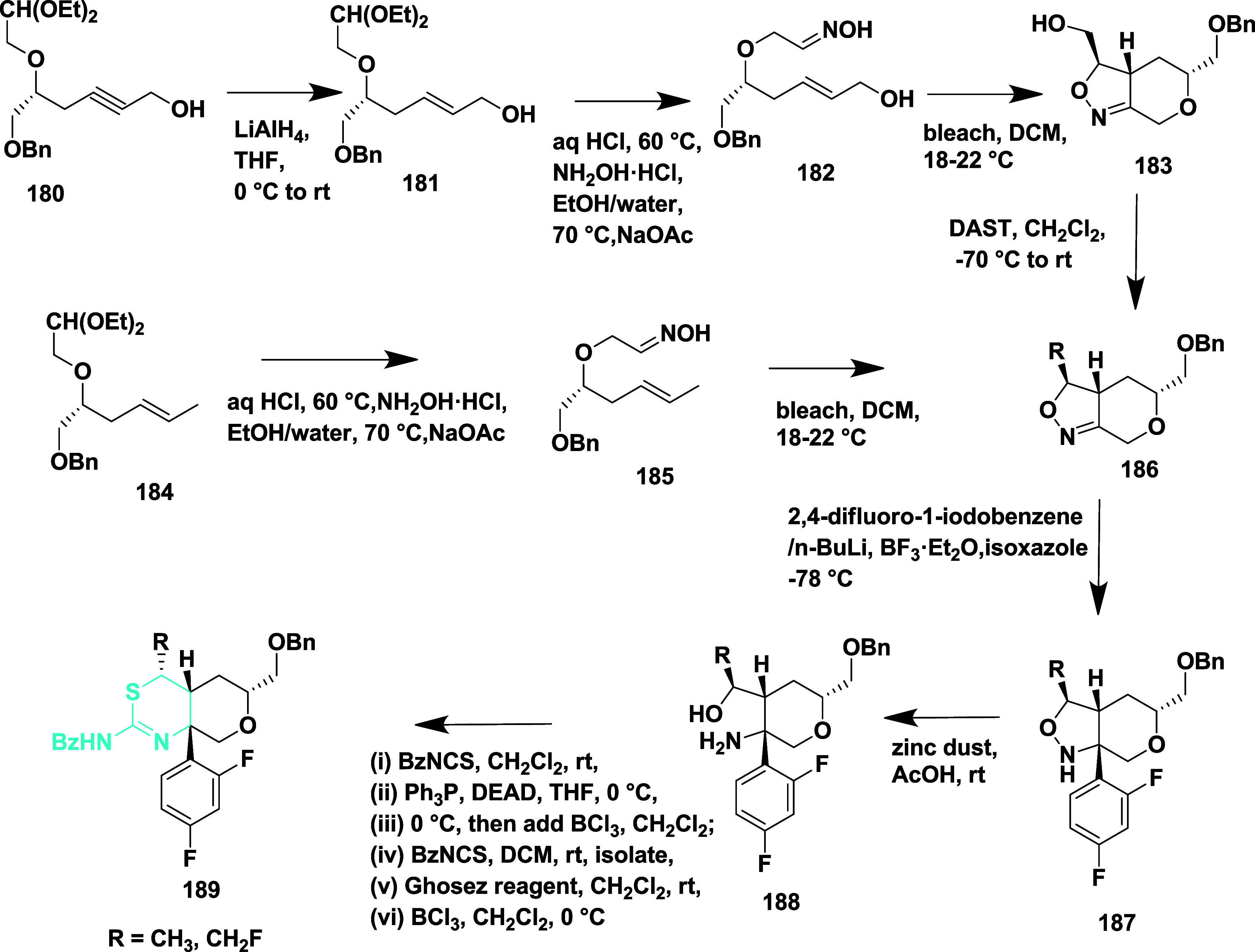
Synthesis of Difluoro Phenyl Substituted 2-Amino-4,5-dihydrothiazole Derivatives **189**

Diisopropylethylamine catalyzed amide coupling of compounds **190** yielded methyl-substituted alcohol **191**. The oxidation of the resulting alcohol with Dess-Martin reagent produced an aldehyde intermediate **192**. The aldehyde **192** was treated with Burgess reagent under microwave irradiation, resulting in the formation of oxazole **193**, followed by 1,8-diazabicyclo [5.4.0] under-7-ene (DBU)-mediated deprotection of **193**, resulting in the desired targeted compound **194** (Scheme 27). The isolated compounds yielded in the range of 64 to 87%. Compound **195** (Table 3) (synthesized similarly as described in Scheme 26) showed good inhibition with an IC_50_ value of 53 nM (WCA) by forming a hydrogen bond with Asp32 and Asp228. The SAR analysis revealed that, initially, modification of the tetrahydropyran (THP) ring by incorporating heteroaryl groups such as oxazole, isoxazole, and pyrazole to better engage with the S2′ subpocket of the enzyme. This strategy led to compounds with improved potency and lipophilic efficiency. Among them, pyrazole-containing derivatives exhibited potent BACE-1 inhibition and effective CNS penetration but were highly susceptible to CYP2D6-mediated metabolism. Small substituents like methyl and fluoromethyl were introduced to the thioamidine sulfur, yielding compounds **194** and **195** to mitigate this. These analogs retained vigorous activity while showing reduced CYP2D6 metabolism, enhanced metabolic stability, and promising efficacy in vivo. Additionally, lowering the basicity of the compounds contributed to safer hERG profiles. Collectively, the modifications led to candidates with a balanced profile of potency, brain availability, metabolic resilience, and safety, supporting their advancement in drug development.

**Scheme 27:**
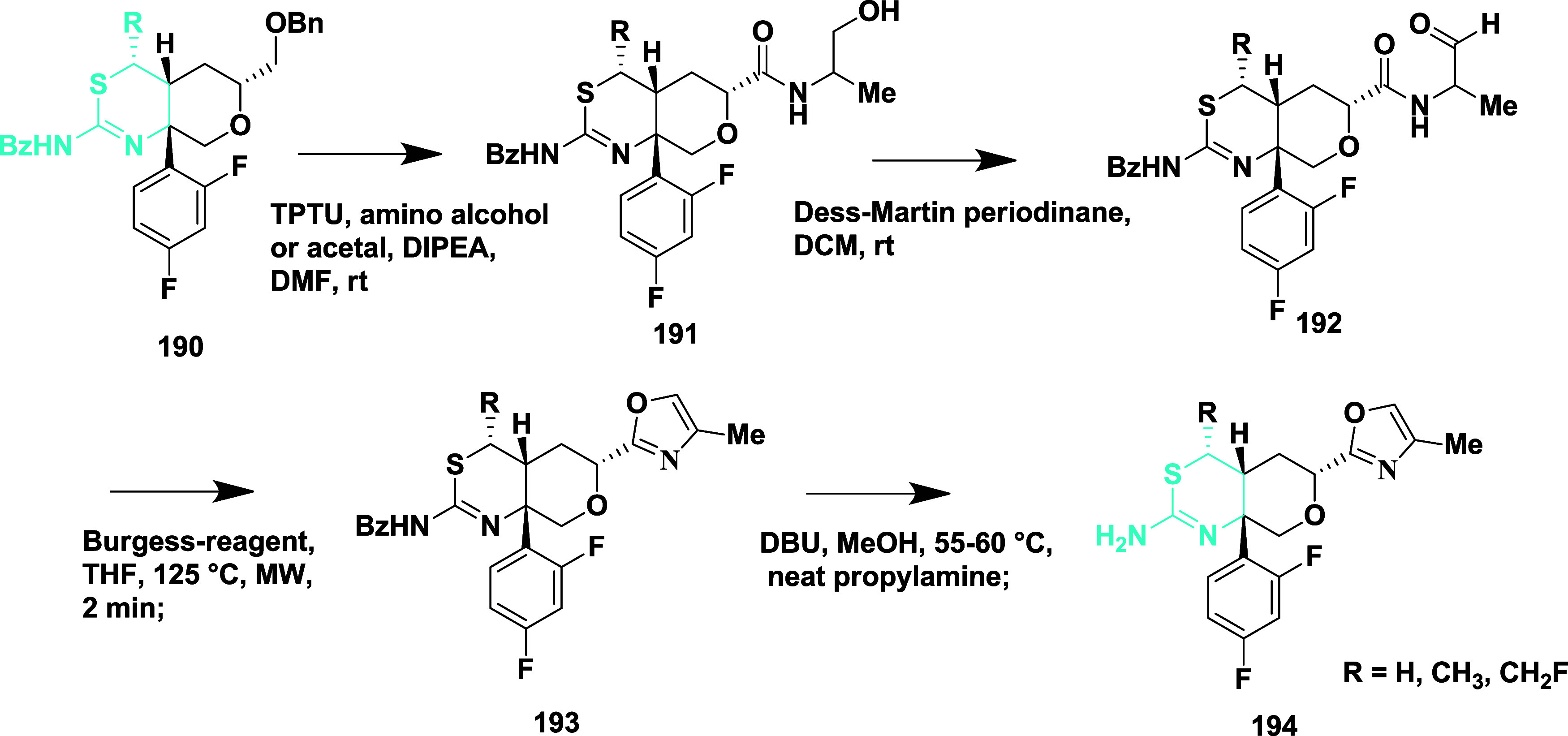
Synthesis of 3-Methylfuran Substituted 2-Amino-4,5-dihydrothiazole Derivatives **194**

**Table 3:**
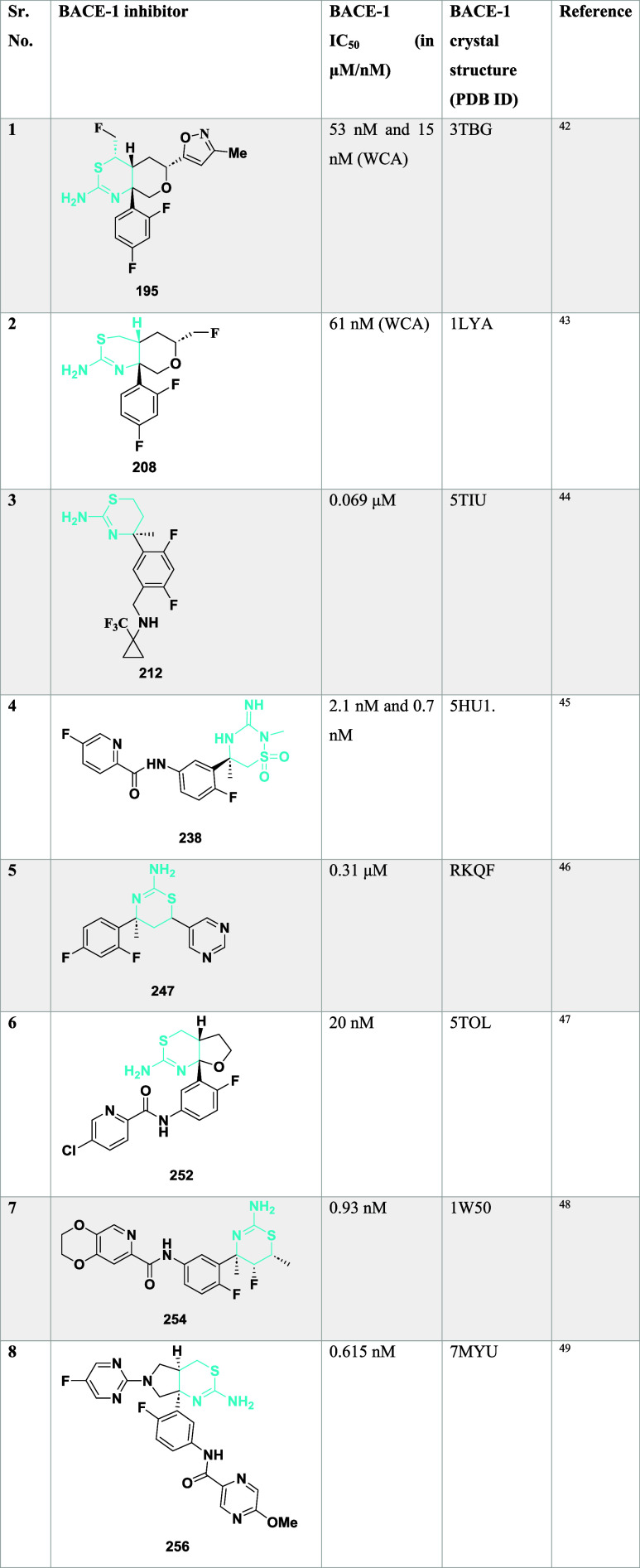
BACE-1 Inhibitors, the Co-Crystal Structure of BACE-1 Enzyme, and Their IC_50_ Values

Butler et al. reported the synthesis of an efficient BACE-1 inhibitor.^43^ The synthesis of **199** begins with the palladium-catalyzed reaction with 3-bromobut-3-en-1-yl 4-methylbenzenesulfonate with (2,4-difluorophenyl) boronic acid **196**, resulting in **197**, followed by treatment with thiourea and acid-catalyzed cyclization of intermediate **198**, yielding 17% of **199** (Scheme 28).

**Scheme 28:**
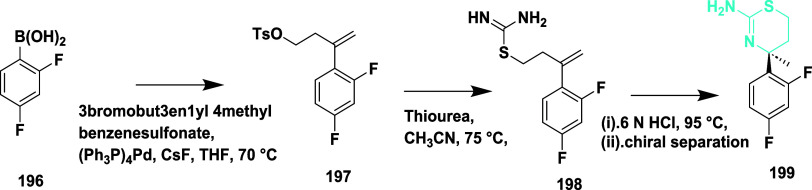
Synthesis of 2,4-Difluorophenyl Substituted 1,3-Thiazin-2-amine **199**

Sodium hydride catalyzed the reaction of (*R*)-homoallylic alcohol **200** with 2-bromo-1,1-diethoxyethane, followed by acidic cleavage, yielding an intermediate **202**, which further reacted with ammonium hydroxide-hydrochloride, resulting in **203**. TEA catalyzed the cyclization of **203** by reacting with sodium oxychloride, yielding **204**. Then, further treated with boron trifluoride diethyl etherate: isopropyl ether in toluene (1:1), and 1-bromo-2,4-difluorobenzene yielded **205**. Zinc-catalyzed ring opening of **205** and treatment with thioisocyanate substituent yielded **207**, followed by cyclization of **207**, which yielded the targeted compound **208** (Scheme 29). The synthesized compounds were isolated in a yield of 50 to 64%. This study focused on optimizing thioamidine-based BACE-1 inhibitors with high central nervous system (CNS) penetration while minimizing off-target activity, particularly against Cathepsin D, by improving selectivity through the use of conformationally constrained fused-ring systems such as tetrahydrofuran (THF) and tetrahydropyran (THP), which effectively stabilized bioactive conformations and enhanced occupancy of the S2′ pocket. Modifications on the THP ring, including small alkyl and fluorinated substituents, increased potency, reduced P-gp efflux, and improved metabolic properties. Among the resulting analogs, compound **208** (Table 3), bearing a fluoromethyl group, demonstrated potent BACE-1 inhibition, robust CNS exposure, significant Aβ-lowering effects in both rodent and canine models, and evidence of amyloid plaque reduction in Alzheimer’s disease mouse models.

**Scheme 29:**
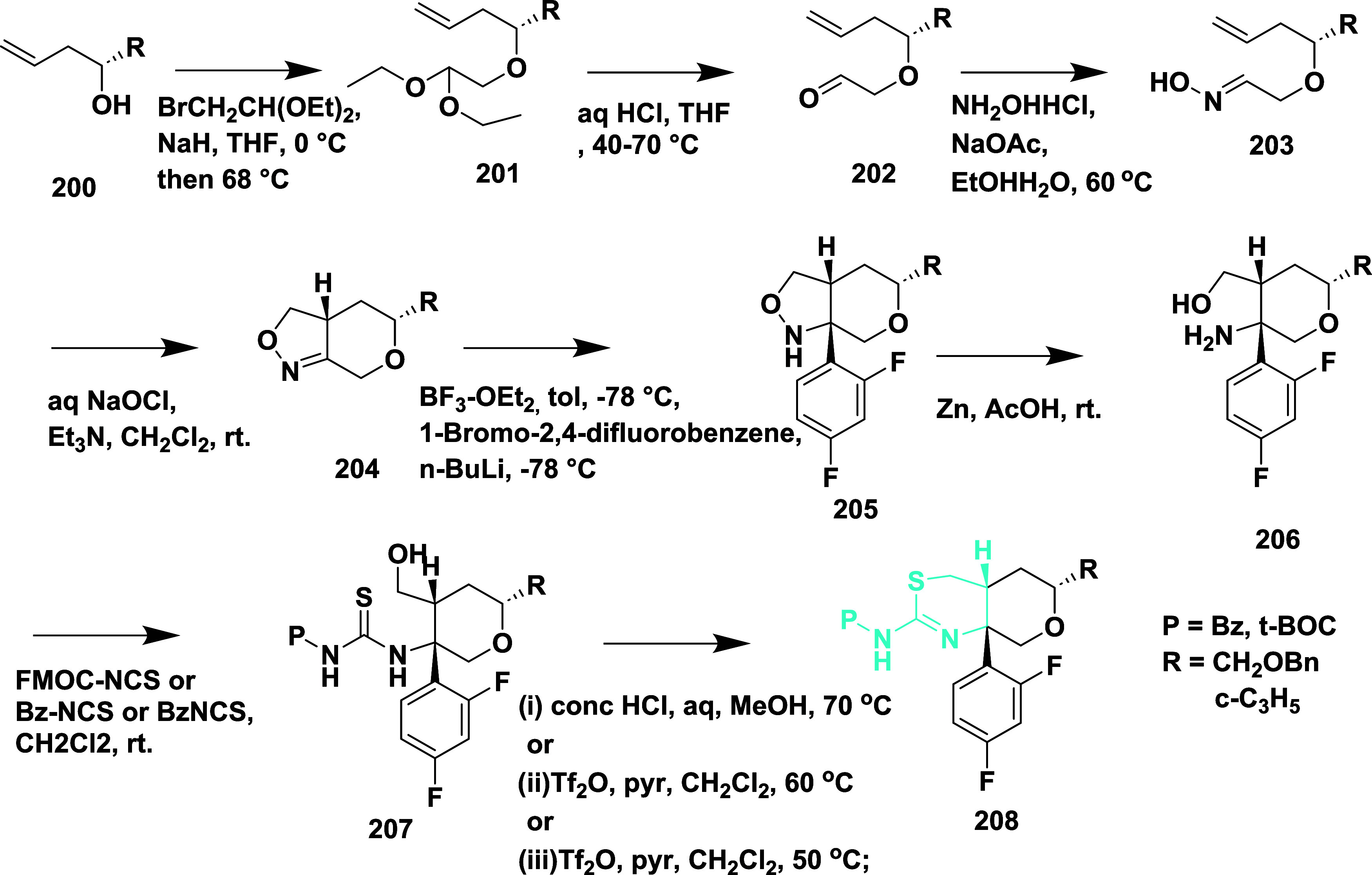
Synthesis of 2,4-Difluorophenyl-1,3-thiazin-2-amine Substituted Derivative **208**

Butler et al. reported synthesizing aminomethyl-based derivatives as a BACE-1 inhibitor.^44^ Formylation of carbamate **209** provided aldehydes **210**. The reductive amination of **210**, in the presence of sodium cyanoborohydride, yielded amidine **211**. The protecting group of **211** was removed under acidic conditions, yielding 19–97% of **212** (Scheme 30).

**Scheme 30:**
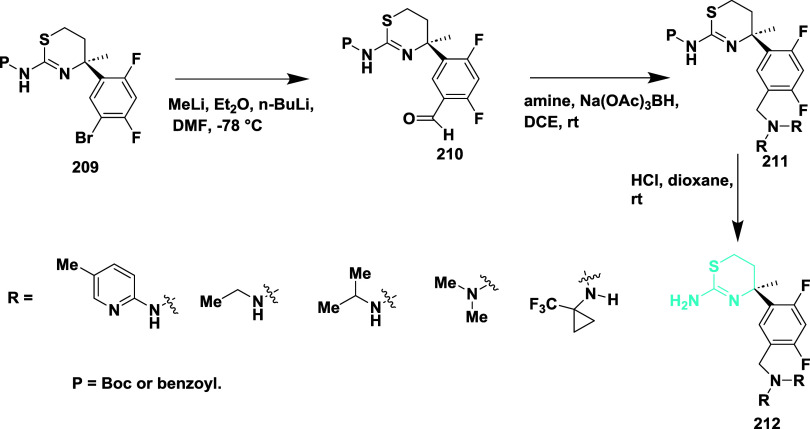
Synthesis of (2,4-Difluorophenyl) Methanamine Substituted 2-Amino-4,5-dihydrothiazole Derivatives **212**

The synthesis begins with a cyanation reaction, where **213** underwent palladium-catalyzed coupling reaction using Pd_2_(dba)_3_, dppf, zinc, and Zn­(CN)_2_ to afford the nitrile intermediate **214**, followed by reduction using raney nickel yields corresponding amines in the presence of hydrogen gas, triethylamine, and Boc_2_O, yielding Boc-protected amine **215**. The protecting group was removed under acidic conditions in methanol media and subsequently reacted with 2,2,2-trifluoroethylsilyl isothiocyanate in MeCN at 70 °C in the presence of DBU, affording final thiazolyl derivative **216**. (Scheme 31). The synthesized compounds were isolated with yields ranging from 19 to 97%. The **212 (**R = H, (trifluoromethyl)­cyclopropane) (Table 3) showed good inhibition with an IC_50_ value of 0.069 μM by hydrogen bond formed with the carbonyl group of Gly230, and it occupied the S1 and S3 binding potencies of the enzyme significantly. BACE-1 inhibitors featuring an aminomethyl linkage have shown significant potential as substitutes for conventional anilide-based structures, offering advantages such as enhanced brain penetration, improved metabolic stability, and reduced risk of hERG channel inhibition. SAR investigations revealed that incorporating small alkyl or ether substituents and electron-withdrawing groups like trifluoromethyl and cyano improved potency and lowered susceptibility to P-gp-mediated efflux. Compound **212** demonstrated effective BACE-1 inhibition and CNS exposure, overcoming CYP2D6-related metabolism issues by adding a fused tetrahydropyran ring. These results support the aminomethyl moiety as a promising design element for refining the therapeutic profile of BACE-1-targeting agents.

**Scheme 31:**
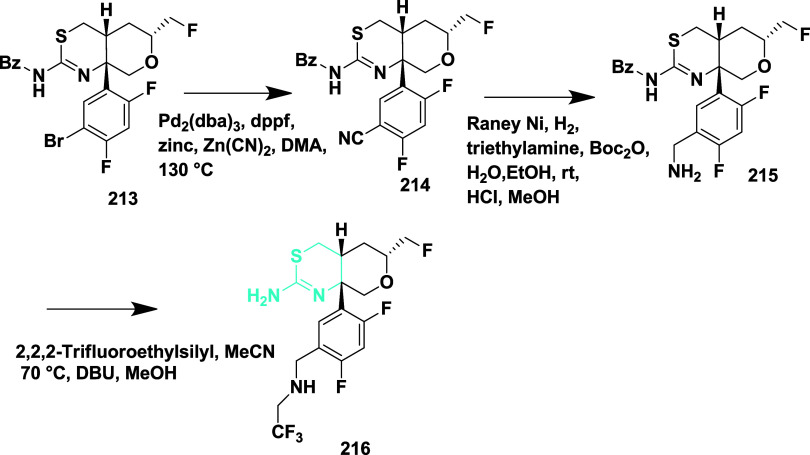
Synthesis of Difluorophenyl-trifluoroethylaminomethyl-pyranothiazinylamine **216**

Scott et al. reported synthesizing a new series of imidathiadiazine dioxide as a BACE-1 inhibitor.^45^ Synthesis of imidathiadiazine dioxide **225** by treating a conjugate base of sulfonamide **219** with (*R*)-*tert*-butyl sulfinyl ketamine **218**, obtained from **217**. The reaction yielded protected β-amino sulfonamide **220**. Hydrochloric acid-catalyzed cleavage of *tert*-butyl sulfonamide **220**, followed by trifluoroacetic acid, was used to remove paramethylbenzaldehyde, resulting in the formation of β-sulfonamide **221**. The amine **221** was benzoyl isothiocyanate, and it underwent cleavage of the benzoyl group, producing a thiourea intermediate, followed by methyl-induced intermolecular ring closure, yielding imino thiadiazinane dioxide **222**. BOC-protected guanidine forms **223**, followed by the bromination of the thiophene ring, generating intermediate **224**. The Suzuki coupling of **224** with (5-(prop-1-ynyl) pyridine-3-yl) boronic acid, followed by BOC deprotection, yielded biaryliminothiadiazine dioxide **225** (Scheme 32).

**Scheme 32:**
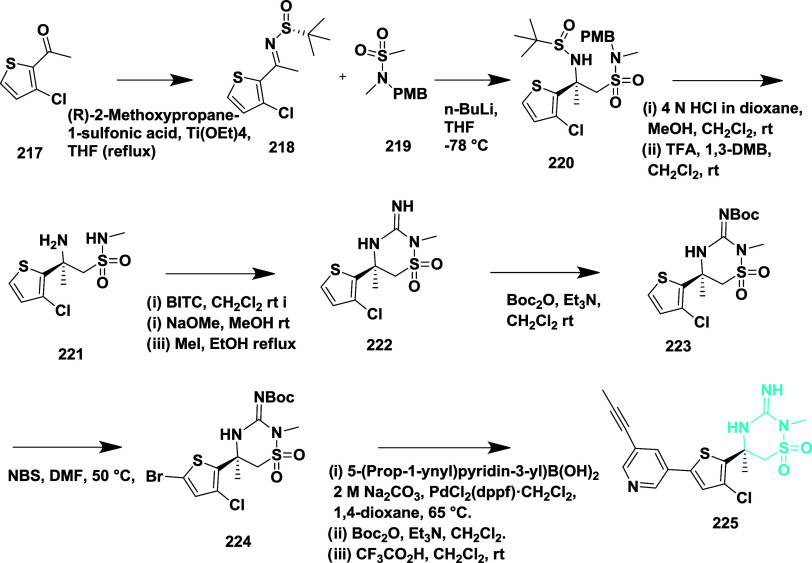
Synthesis of Prop-1-yn-1-yl Pyridine Substituted 1,2,4-Thiadiazinane 1,1-Dioxide **225**

Ketamine **227** was obtained from methyl ketone **226** when subjected to an aldol addition with the enolate of methyl acetate, yielding β-amino ester **228**. The amine produced from the acidic cleavage of sulfonamide was added to *N*-[(methylamino)­thioxomethyl]-butyl carbamate. The iminopyrimidinones core, which was Boc protected to produce **229**, was made possible by subsequent ring closure. The amide coupling with pyridine carboxylic acids and Boc deprotection, followed by nitro reduction, yielded the iminopyrimidinones carboxamide analogs **231** ^45^ (Scheme 33).

**Scheme 33:**
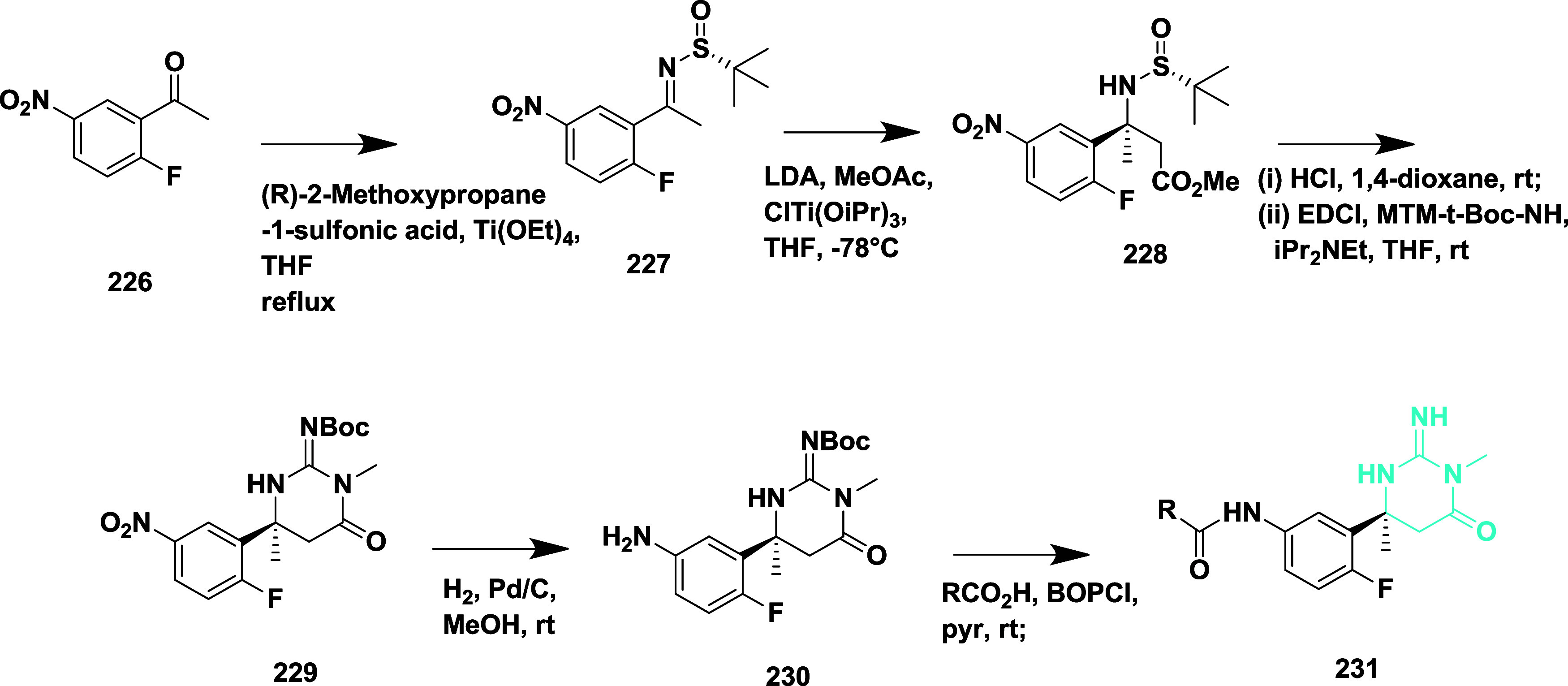
Synthesis of Fluorophenyl-imino-dimethylhexahydropyrimidinyl-acetamide Derivative **231**

Amino sulfonamide **234** was produced by the reaction of ketamine with the lithium anion of sulfonamide **233**, followed by the acidic cleavage of sulfonamide and *p*-methoxybenzyl (PMB) groups to yield the β-amino sulfonamide derivative **235**. Subsequently, cyanogen bromide was used to form the imino thiadiazinane dioxide ring. After the ring formation, the guanidine was protected with the Boc group, yielding **236**, and nitro reduction furnished aniline **237**. Finally, the heterocyclic imino thiadiazinane dioxide amides **238** were produced by amide formation and deprotection of the amine **237** (Scheme 34). The yield of the isolated compounds ranges from 35 to 90%. The verubecestat **238** (if R = 3-fluoropyridine) (Table 3) is the most potent among this series and potentially inhibited the Aβ-40 and Aβ-42 with IC_50_ values of 2.1 and 0.7 nM, respectively, by occupying the hydrophobic S1 pocket of the enzyme. SAR analysis indicated that the 3-imino-1,2,4-thiadiazinane 1,1-dioxide scaffold led to the discovery of verubecestat (MK-8931), a potent and brain-penetrant BACE-1 inhibitor. Strategic replacement of carbonyl-containing motifs with a sulfonyl group provided conformational rigidity while enhancing membrane permeability and CNS distribution. Incorporation of a picolinamide side chain improved binding affinity within the S1–S3 subpocket and significantly increased selectivity over cathepsin D. Additional fluorine substitution on the pyridine ring further optimized potency and pharmacokinetic performance. Verubecestat exhibited robust Aβ-lowering activity and favorable brain exposure across preclinical species and humans, ultimately progressing to Phase 3 clinical trials. These findings underscore the importance of balancing potency, permeability, metabolic stability, and off-target selectivity in developing clinically viable BACE-1 inhibitors.

**Scheme 34:**
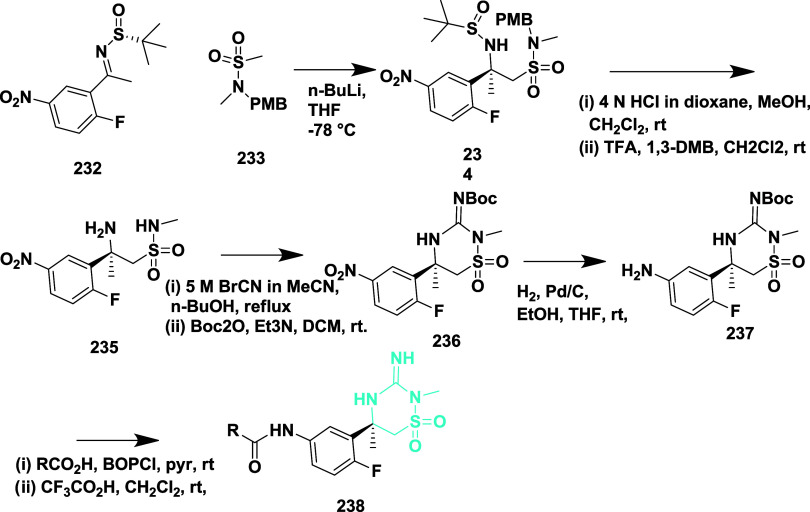
Synthesis of 4-Fluoro Substituted 1,2,4-Thiadiazinan-5-ylacetamide Derivatives **238**

Wu et al. synthesized substituted aminothiazine with a heteroaryl group as a new inhibitor class.^46^ The synthesis begins with the lithiation of compound **239** with *n*-butyllithium, generating intermediates that undergo cyclization and trifluoracetic acid deprotection in dichloromethane, which produces 57% of the targeted compound **240** (Scheme 35).

**Scheme 35:**
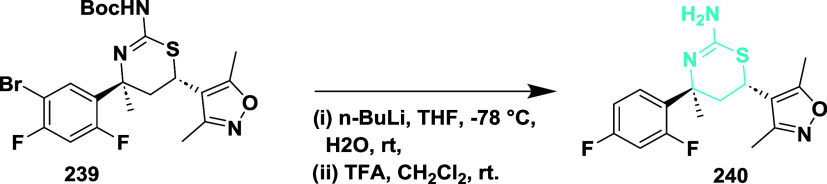
Synthesis of 2,4-Difluorophenyl Substituted Compound **240**

In a stereoselective addition reaction, *N*-*tert*-butanesulfinyl imine **241** reacts with the enolate of methyl ketone **242**, yielding an intermediate *N*-sulfinyl β-aminoketone **243**. The tritertbutylaluminum-mediated reduction of **243** afforded amino alcohol **244**. The removal of sulfinyl from **244** afforded syn 1,3-amino alcohol **245**, then it was treated with benzoyl thiocyanate to form an intermediate **246**. Finally, under acidic conditions, **232** was converted to the target molecule **247** with a yield of 42% (Scheme 36). Compound **247** showed good BACE-1 inhibition with an IC_50_ value of 0.31 μM. Structure–activity relationships (SAR) of aminothiazine-based BACE-1 inhibitors to enhance brain penetration by truncating the S3 substituent, thereby reducing molecular weight and polar surface area. While this modification slightly reduced potency, it significantly improved CNS exposure and robust in vivo Aβ-lowering activity. Various alternative heterocycles were evaluated to address metabolic instability associated with the 6-dimethylisoxazole moiety. Among these, compound **247** (Table 3), incorporating a pyrimidine ring, maintained potent BACE-1 inhibition, demonstrated enhanced metabolic stability across preclinical species, and achieved superior Aβ-lowering efficacy in rodent models. X-ray cocrystal structures confirmed the preservation of key binding interactions, supporting the overall design strategy and reinforcing the value of S3 truncation and heteroaryl optimization at the C-6 position in advancing CNS-active BACE-1 inhibitors.

**Scheme 36:**
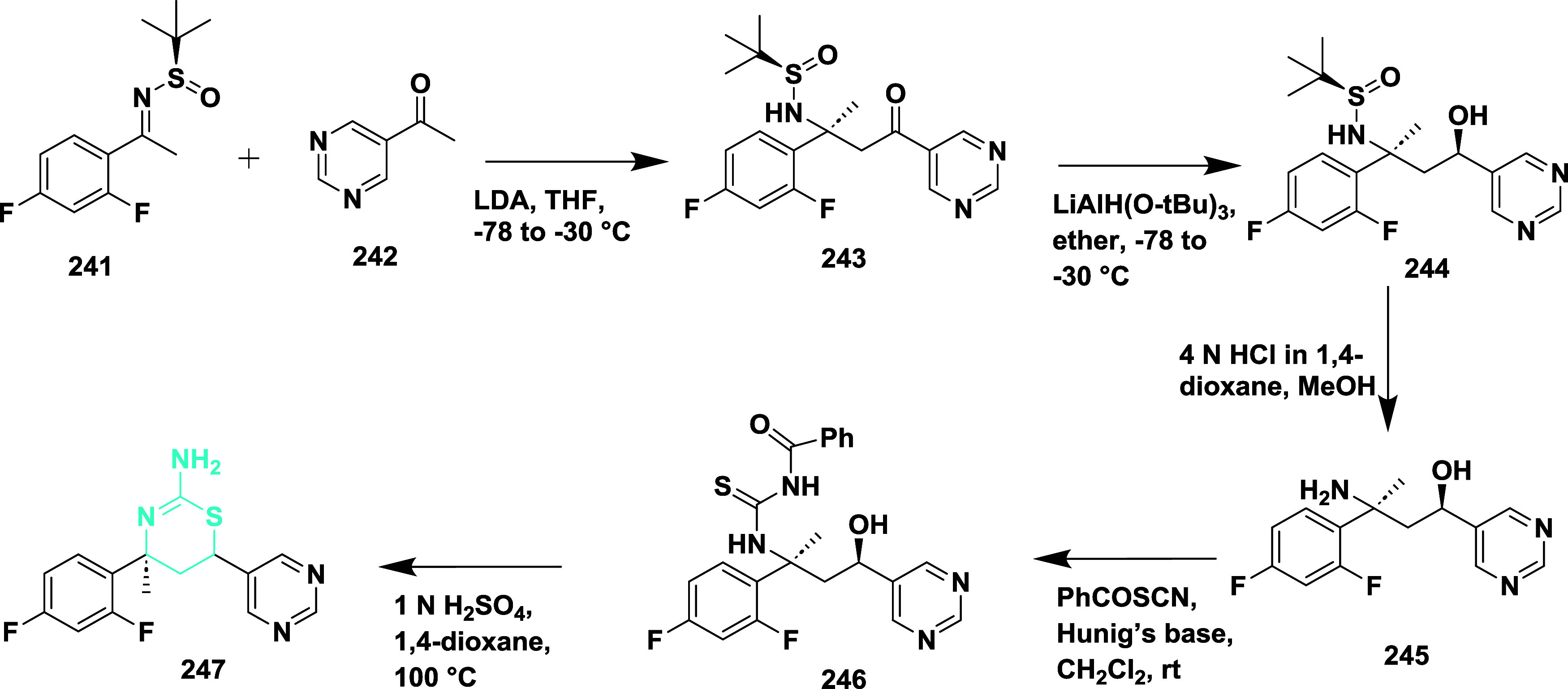
Synthesis of 2,4-Difluorophenyl and Pyrimidin-5-yl Substituted Compound **247**

Wu et al. reported the synthesis of new fluoro­[2,3-*d*] [1,3] thiazinamines as β-secretase inhibitors.^47^ The synthesis of fluoro­[2,3-*d*] [1,3] thiazinamines began with the azidation of bromide intermediate **248**, which produced an intermediate **249**, further reduced and Boc protected to yield Boc-aniline **250**. The coupling of **250** with various heteroaryl acids gave amide intermediate **251**, and cleavage of Boc, producing a target compound **252** (90%) (Scheme 37). Fluoro­[2,3-*d*]­[1,3]­thiazinamine derivatives were developed as simplified BACE-1 inhibitors inspired by LY2886721, aiming to retain potency with improved CNS drug-like properties. SAR studies revealed that C5-substitution on the picolinamide side chain (e.g., Cl, Br, CN) enhanced binding affinity by engaging the S3 subpocket. Potent compound **252** (if X = Cl, Y = CH, Z = H) (Table 3) showed strong in vitro activity and favorable permeability profiles. However, in vivo Aβ-lowering effects were limited, likely due to reduced brain exposure. Despite this, the scaffold presents a promising starting point for further optimization as a bioisosteric alternative in BACE-1 inhibitor design.

**Scheme 37:**
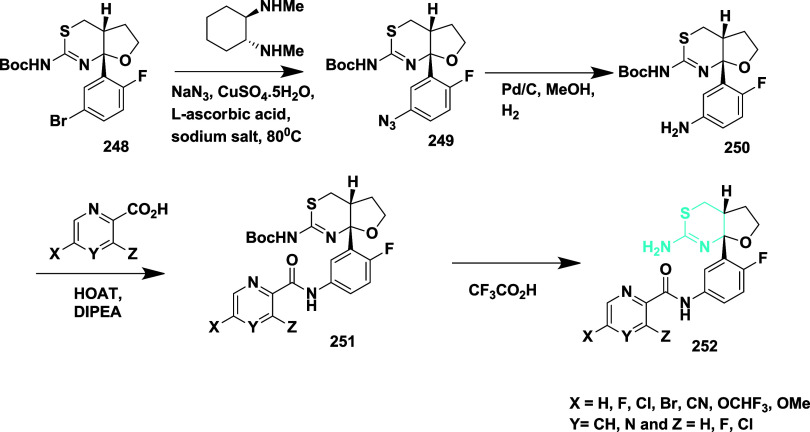
Synthesis of 2-Amino-4,5-dihydrothiazole Derivatives **252**

Ueno et al. reported the synthesis of fused pyridine derivatives as β-secretase inhibitors using (4*R*,5*R*,6*R*)-4-(5-amino-2-fluorophenyl)-5-fluoro-4,6-dimethyl-5,6-dihydro-4*H*-1,3-thiazin-2-amine- amine core **253**.^48^ The synthesis involved amide coupling **253** with various bicyclic pyridine carboxylic acids using EDC·HCl as a coupling agent and hydrochloric acid were used to protonate the 1,3-thiazine nitrogen at room temperature in methanol media, yielding dihydro1,3-thiazine derivatives 254 in yields ranging from 15 to 100% (Scheme 38). A fused pyridine derivatives were developed to enhance BACE-1 selectivity by targeting the flexible 10s loop adjacent to the S3 pocket. Structure–activity relationship (SAR) studies guided key modifications, particularly at the tail region, to optimize interactions with the open conformation of the BACE-1 10s loop while reducing affinity for the rigid BACE-2 loop. The compound **254** (if R = 7-methyl-2,3-dihydro- [1,4] dioxino [2,3-*c*] pyridine) (Table 3) showed good inhibition with an IC_50_ value of 0.93 nM, showing over 500-fold enzymatic and 1400-fold binding selectivity for BACE-1. Structural analyses confirmed its favorable fit within BACE-1 and destabilizing effect on BACE-2. The compound also demonstrated strong in vivo Aβ-lowering activity and promising pharmacokinetic properties.

**Scheme 38:**
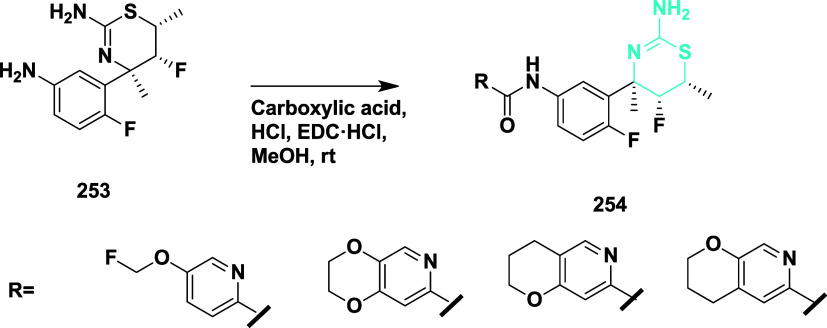
Synthesis of *N*-(3-((4*R*,5*R*,6*R*)-2-Amino-5-fluoro-4,6-dimethyl-5,6-dihydro-4*H*-1,3-thiazin-4-yl)-4-fluorophenyl) Acetamide Derivatives **254**

Synthesis of CNS-penetrating BACE1 inhibitor reported by McKinzie et al.^49^ The synthesis started from diamine intermediate **255**, which undergoes amide coupling with various aryl carboxylic acids in acetonitrile/ethanol at 50 °C produced 2,6-difluoropyridine/4-methoxy pyrazine/4-methoxypyrazine carboxamide derivative **256** with a yield in the range from 40 to 75% (Scheme 39). Carboxamide derivatives demonstrated effective BACE-1 inhibition, favorable CNS penetration, and reduced β-amyloid levels while addressing safety concerns such as retinal toxicity and liver enzyme elevation. Compound **256** (LY3202626) (Table 3), featuring a 2-methoxy-5-methylpyrazine moiety, exhibited potent inhibitory activity with an IC_50_ of 0.615 nM. SAR studies emphasized strategies like S3 moiety truncation and thiophene incorporation to enhance brain permeability and ligand efficiency, though CatD selectivity was compromised. Analogues with reduced p*K*_a_ and log *P* were developed to overcome lysosomal trapping, leading to improved CNS distribution. Optimization with amide-containing S3-binding groups ultimately yielded LY3202626, a potent, selective, and brain-penetrant candidate suitable for clinical advancement.

**Scheme 39:**
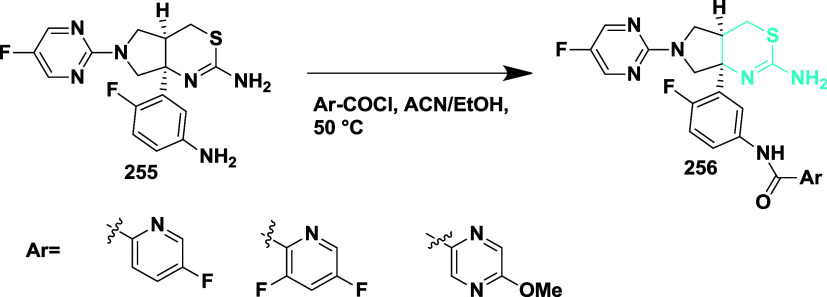
Synthesis of *N*-(3-((4a*R*,7a*S*)-2-Amino-6-(5-fluoropyrimidin-2-yl)-4,4a,5,6,7,7a-hexahydropyrrolo­[3,4-*d*]­[1,3]­thiazin-7a-yl)-4-fluorophenyl)­acetamide Derivatives **256**

## Dihydro Pyrimidines and Dihydropyridines as BACE-1 Inhibitors

Dihydropyrimidines and dihydropyridines are six-membered heterocycles that play a vital role in drug discovery.^50^ These core structures are known for their wide range of pharmacological activity, such as antibacterial, antimalarial, antiviral, anticancer, antianginal, etc. The structural versatility of both (like flexible chemical structure allowing various substitutions) enhances the binding efficiency and specificity of inhibitors with enzymes.^51^ Mainly, dihydropyridines were known for their optimal pharmacokinetic profile, such as BBB permeability and bioavailability.^51^

Based on dihydropyrimidines scaffold and their imino and thia analogs, Bais and co-workers synthesized a novel BACE-1 inhibitor^52^ 260 through a one-pot Biginelli reaction using various aldehydes **258**, β-dicarbonyl compounds **257**, and urea (or thiourea) **259** under solvent-free conditions using ammonium chloride as a catalyst (Scheme 40). The compounds were isolated in yields ranging from 50 to 92%. The SAR studies in this work revealed that all synthesized dihydropyrimidinone (DHPM) derivatives featuring either sulfur (X = S) or imino (X = NH) groups demonstrated notable inhibitory activity against BACE-1, with IC_50_ values ranging from 0.2 to 71 μM. Among them, compound **260** (Table 4) emerged as the most potent, exhibiting an IC_50_ of 0.32 μM. This compound incorporated a phenyl moiety at the R_1_ position and a methyl ester at R_2_, effectively engaging the target enzyme’s S1 and S2′ binding pockets. Substitution with sulfur or imino groups consistently yielded higher activity than their oxygen-containing counterparts (X = O), suggesting that these functionalities better support key interactions within the active site. Moreover, the nature of the R_2_ substituent played a critical role, with methyl esters outperforming ethyl esters, amides, and carboxylic acids. The latter groups appeared to reduce activity, potentially due to unfavorable ionization states within the enzyme’s active site. Overall, the enhancement in inhibitory potency was linked to molecular features facilitating hydrogen bonding and hydrophobic interactions at the S1 and S1′ subsites of BACE-1.

**Scheme 40:**
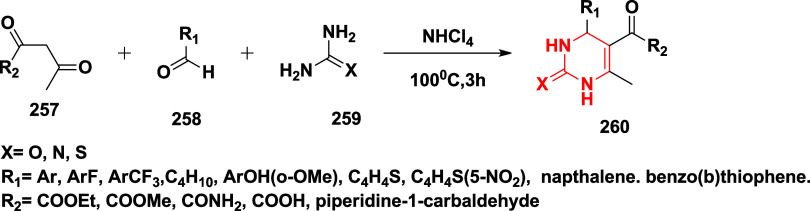
Synthesis of Dihydropyrimidines **260**

**Table 4:**
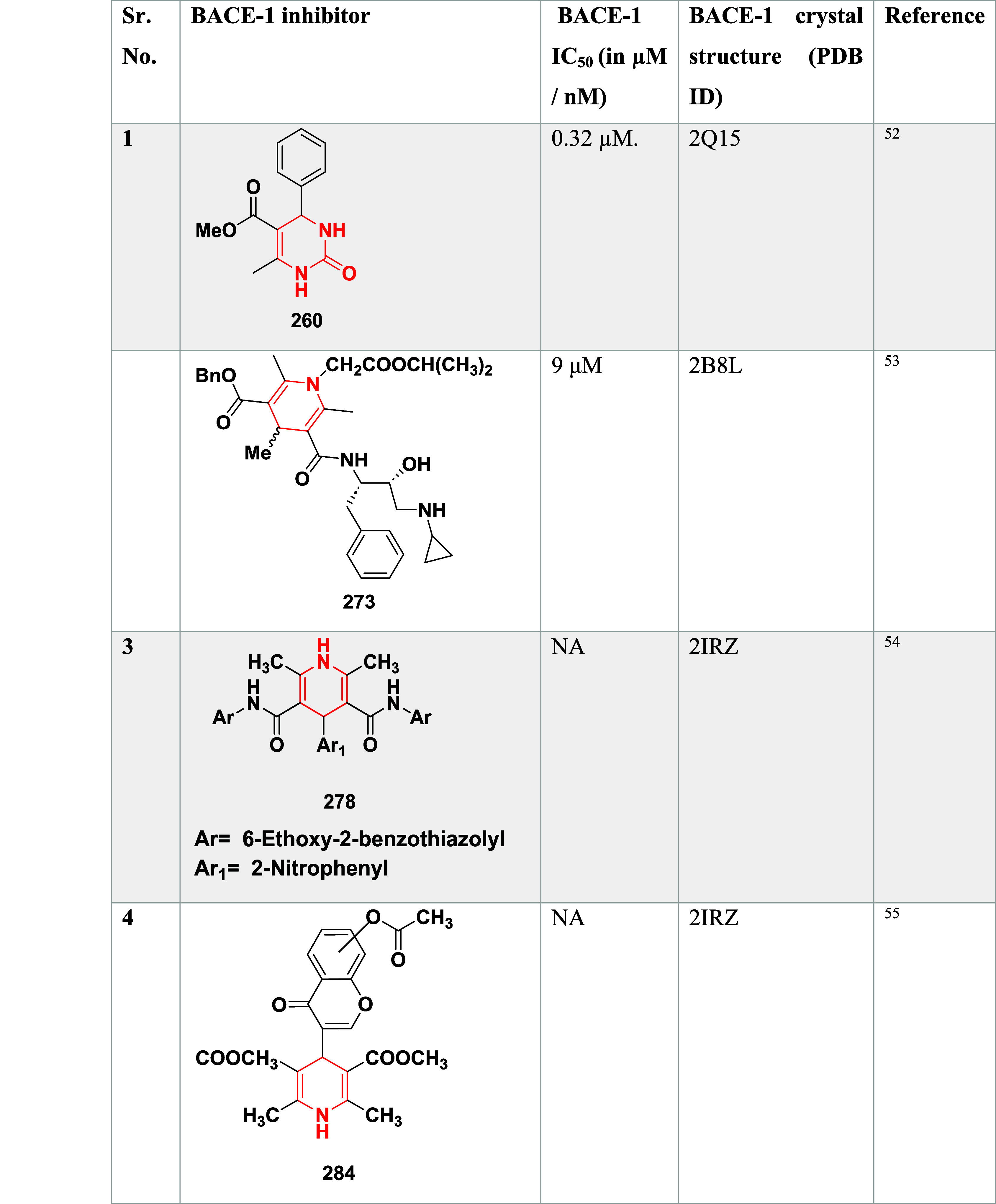
BACE-1 Inhibitors, the Co-Crystal Structure of BACE-1 Enzyme, and Their IC_50_ Values

Choi et al. synthesized a series of 1,4-dihydro pyrimidine (DHP) derivatives through a one-pot reaction, a PyBOP-mediated coupling reaction, and using Lewis’s acid as a β-secretase inhibitor.^53^ The 1-methyl sulfonamide-2,6-dimethyl-1,4-dihydropyridine derivatives **267** were obtained from 1,4 DHP dibenzyl ester **261** through mesylation of the N-1 position of the ester and followed by selective monohydrolysis catalyzed by Lewis acid; this leads to the formation of an intermediate **264**. A PyBOP-mediated amide coupling of (*R*)-methyl benzylamine with **253**, followed by hydrolysis to yield an ester **265**. Ester **265** coupled with (2*R*,3*S*)-3-amino-1-(cyclopropylamino)-4-phenylbutan-2-ol **266**, a hydroxyethylamine (HEA) isostere moiety, resulting in the formation of the target 1,4-dihydro pyrimidine **267** in 65% yield (Scheme 41). A similar approach was employed to synthesize compound **273** (Scheme 42), with isolated yields ranging from 10 to 65%. Compound **273** (if R_1_ = Me, R_2_ = OBn, R_3_ = CH_2_COOCH­(CH_3_)_2_, R_4_ = cyclopropyl) (Table 4) was the most potent in this series (Table 4). The structure–activity relationship (SAR) investigation revealed that substituting the isophthalamide moiety of hydroxyethylamine-based BACE-1 inhibitors with a 1,4-dihydropyridine (DHP) framework yielded analogues with moderate activity, exhibiting IC_50_ values between 8 and 30 μM. Incorporation of bulkier alkyl groups at the R_1_ position, such as propyl, led to a slight improvement in potency, whereas introducing a phenyl group diminished activity. At the R_3_ site, ester functionalities outperformed amide groups, likely due to enhanced hydrogen bonding capacity, and the use of a short carbon linker proved more favorable for engaging the S2 pocket. In contrast, larger aromatic substitutions at R_4_ and the removal of steric hindrance at the 2,6-positions of the DHP ring were detrimental to binding affinity. These findings support the viability of the DHP scaffold, though additional optimization is necessary to match the efficacy of earlier isophthalamide-based leads.

**Scheme 41:**
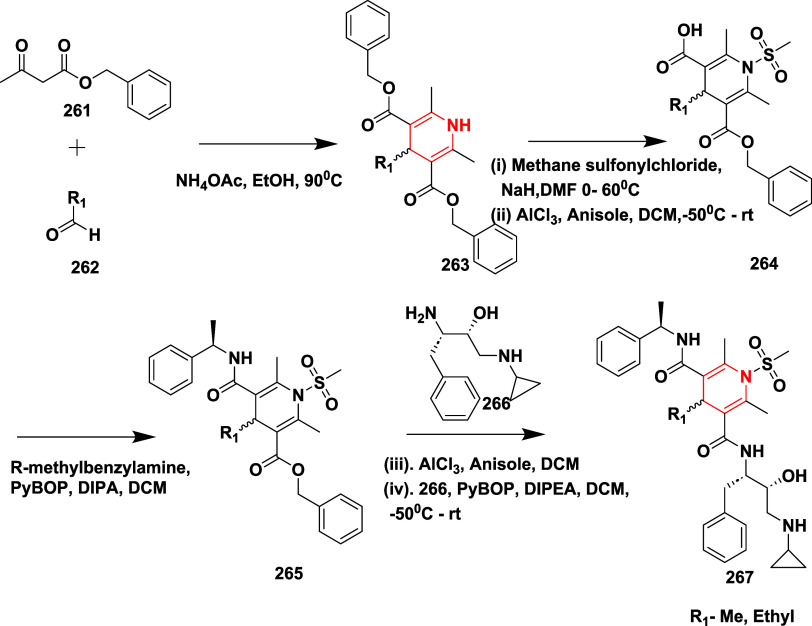
Synthesis of 1-Methyl Sulfonamide Substituted 1,4-Dihydropyridine Derivatives **267**

**Scheme 42:**
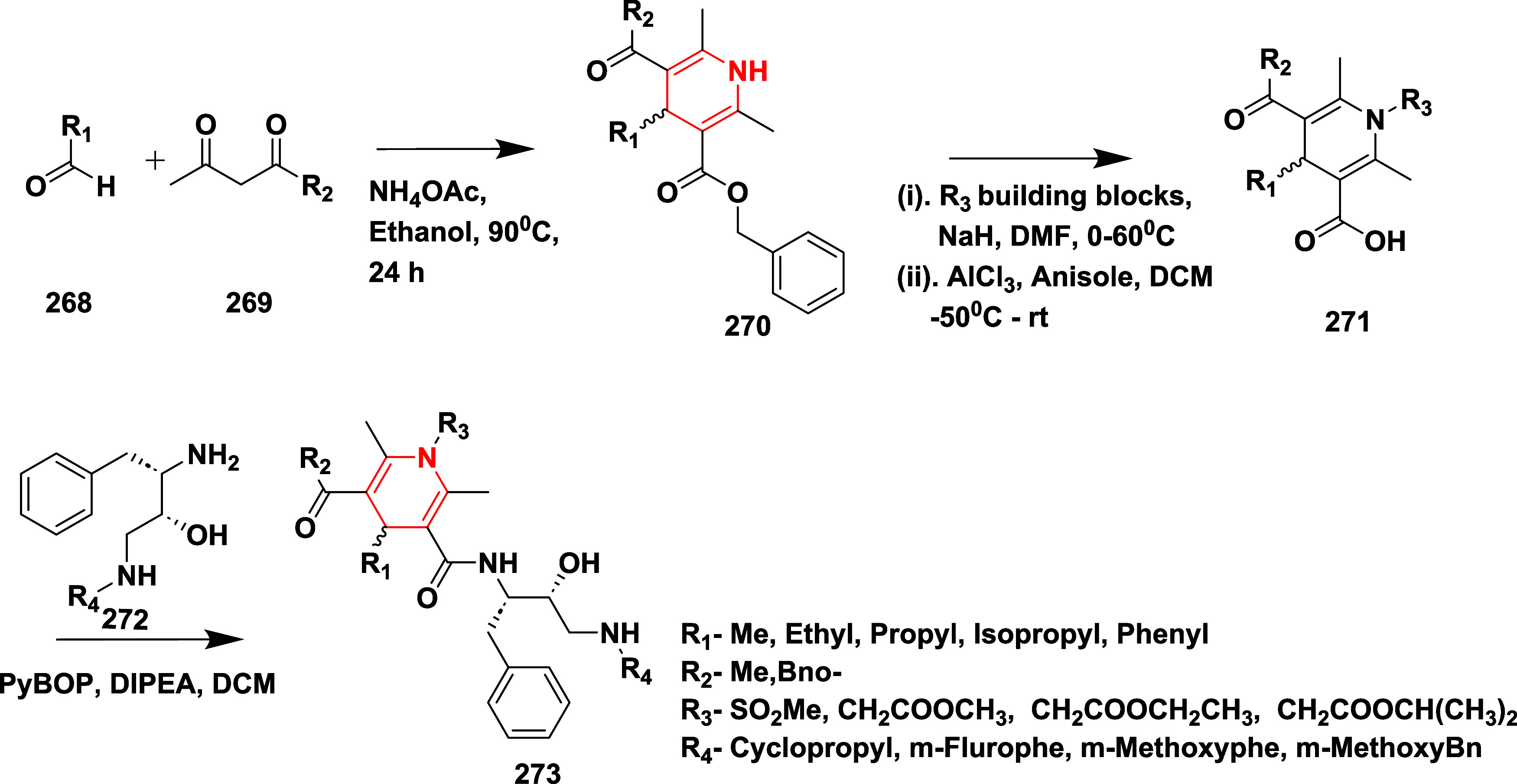
Synthesis of 1-Alkyl Acetate Substituted 1,4-Dihydropyridine Derivatives **273**

Razzaghi-Asl et al. reported the synthesis of novel dihydropyridine (3,5-bis-*N*-(aryl/heteroaryl) carbamoyl-4-aryl-1,4-dihydropyridine) as an inhibitor of AD’s β-secretase.^54^ Synthesis begins with a reaction of (2,6-dimethyl-4-oxo-4*H*-1,3-dioxin-2-yl)­methylium **274** with various aromatic amines **275** in xylene medium, affording the corresponding keto-enamine intermediate **276** and **277**. These intermediates underwent cyclo-condensation reactions with various aldehydes and urea/thiourea in ethanol media in the presence of ammonium acetate to yield the desired 1,4-dihydropyridines **278** with a 12–36% yield (Scheme 43). Compound **278** (if Ar = 6-Ethoxy-2-benzothiazolyl and Ar_1_ = 2-Nitrophenyl) (Table 4) exhibited 83.76% inhibition at 10 μM concentration. SAR studies indicated that the BACE-1 inhibitory activity of 1,4-dihydropyridines (DHPs) highly depended on the aryl or heteroaryl groups at the C3, C4, and C5 positions. Derivatives featuring 2- or 4-nitrophenyl at C4 and thiazole or benzothiazole at C3 and C5 exhibited potent inhibition, with several compounds showing over 70% activity at 10 μM. Effective binding was attributed to key hydrogen bonds formed by the amide NH groups at C3 and C5 with Asp228 and Gly230 in the BACE-1 active site. Substitution with dimethylamino groups at C4 led to reduced potency, likely due to unfavorable protonation and desolvation effects. While isoxazole-based analogues were generally less active, some maintained potency through enhanced lipophilic interactions. This underscores the need to carefully balance hydrogen bonding, hydrophobic forces, and electronic characteristics for optimal enzyme inhibition.

**Scheme 43:**
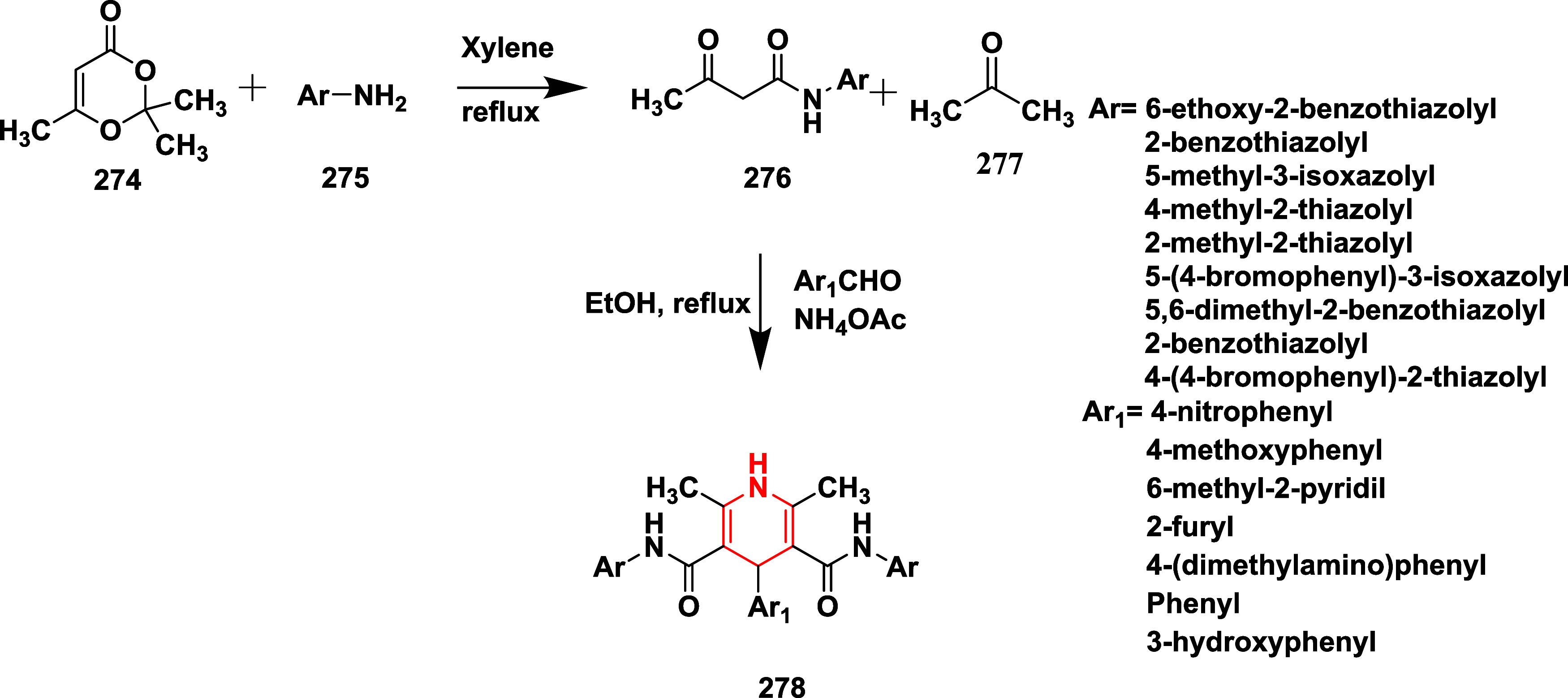
Synthesis of 1,4 1,4-Dihydropyridine Derivatives **278**

In another report, Razzaghi-Asl and co-workers reported synthesizing a series of novel dihydropyridines as inhibitors of AD’s BACE-1.^55^ The compound **279**, synthesized from dihydroxy acetophenone, undergoes monoacetylation with acetic anhydride using pyridine to the hydroxyl group, followed by Vilsmeier–Hack formylation, yielding chroman **281**. The deacetylation of **281** with sodium hydroxide produces hydroxy chroman **282**. The hydroxy chroman **282** undergoes Hantzsch pyridine reaction with β-ketoester, resulting in **283** (hydroxy chromonyl dihydropyridines). The acylation of **283** with hexanoic and acetic anhydride produced compound **284** with a 62–85% yield (Scheme 44). SAR findings revealed that BACE-1 inhibition by 1,4-dihydropyridine derivatives was strongly influenced by the type and position of chromone substitution at the C4 position. Compounds containing the 4-[7-(ethanoyloxy)-4-oxo-4*H*-chromen-3-yl] group demonstrated superior activity compared to those with larger or 6-substituted chromone moieties, with compound **284** (R = C_2_H_5_, R_1_, R_2_ = CH_3_ **)** being the most potent due to minimal steric hindrance. Compound **284** (R = C_2_H_5_, R_1_ = propane, R_2_ = pentane), featuring a bulkier hexanoyloxy group and n-propyl chains at C2/C6, also showed notable activity, likely from enhanced hydrophobic interactions. Docking studies supported these observations, highlighting essential hydrogen bonding with Asp32 and favorable binding within the S1 and S2 pockets. Compound **284** (R = C_2_H_5_, R_1_, R_2_ = CH_3_ **)** (Table 4) exhibited good inhibition among this series with 51.32% at 10 μM concentration.

**Scheme 44:**
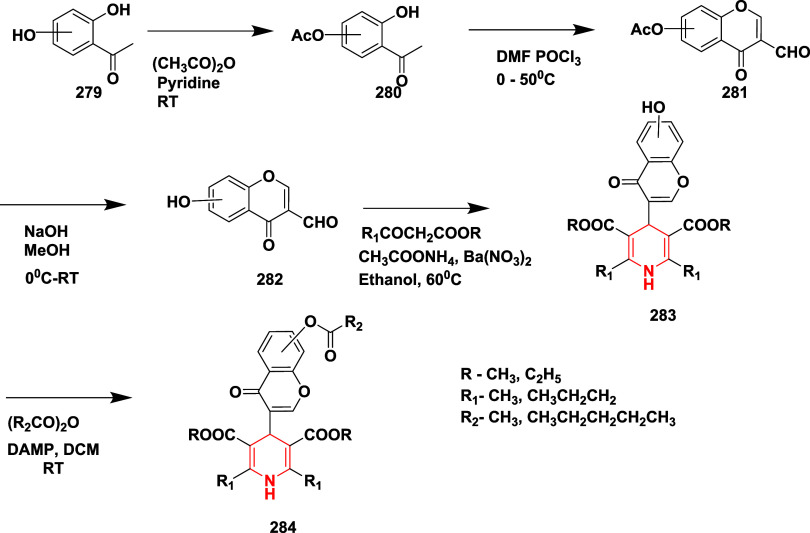
Synthesis of 1,4-Dihydropyridines **284**

## Iminopyrimidinones as BACE-1 Inhibitors

Iminopyrimidinones are a class of heterocycles characterized by a pyrimidinone ring with an amino group. This scaffold’s structural features enhance the inhibitor’s binding efficiency with the binding site of the BACE-1 enzyme.^56^ In preclinical trials, iminopyrimidinones showed a strong Aβ-lowering effect, and the incorporation of the difluorophenyl enhances the specificity and reduces the potential binding interaction.

Stamford et al. reported the synthesis of iminopyrimidinones using an Ellman aldol condensation.^57^ The addition of **286**, derived from 1-(3-chlorothiophen-2-yl)- ethenone **285**, to methyl acetate in the presence of chlorotitanium­(IV) tri isopropoxide (ClTi­(OiPr)_3_) results in (*S*)-β-amino ester derivative **287**. The acidic cleavage of **287**, followed by the resulting intermediates, undergoes a coupling and cyclization reaction to yield **288**. The bromination of **288** resulted in *tert*-butyl (*S*,*E*)-(4-(5-bromo-3-chlorothiophen-2-yl)-1,4-dimethyl-6-oxotetrahydropyrimidin-2­(1*H*)-ylidene)­carbamate **289** with a yield of 46%. This intermediate was then subjected to Suzuki coupling, followed by Boc deprotonation, yielding 45% of iminopyrimidinones **290** (Scheme 45). SAR studies reveal that replacing the iminohydantoin core with a six-membered iminopyrimidinones ring enhanced cellular activity, particularly when a small 6-methyl substituent was used to avoid steric hindrance at Ile118. Biaryl scaffold modifications identified thiophenyl analogs, especially 2,4- and 2,5-substituted derivatives, superior to biphenyl analogs in enzyme binding and cell potency. Further enhancement was achieved by incorporating a 3-propynylpyridyl moiety and chloro substitution on the thiophene ring, yielding compound **290** (Table 5), the most potent inhibitor with a *K_i_* of 1.7 nM and IC_50_ of 11 nM. Compound **290** also demonstrated strong selectivity, favorable physicochemical and pharmacokinetic properties, and significant Aβ-lowering effects in vivo, supporting its potential for clinical development.

**Scheme 45:**
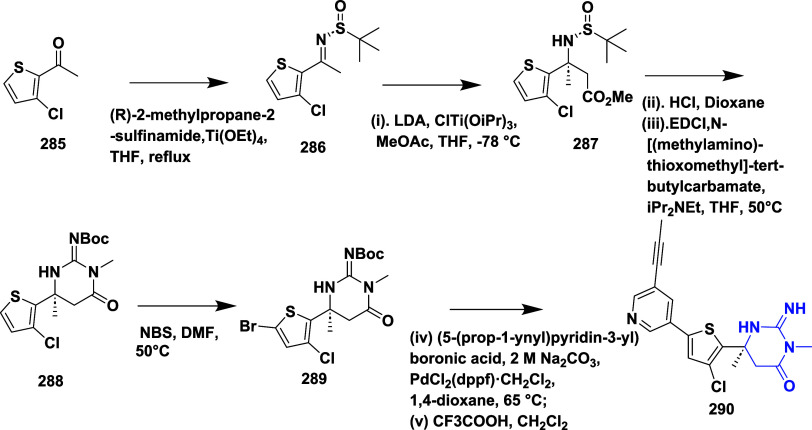
Synthesis of Iminopyrimidinones **290**

**Table 5:**
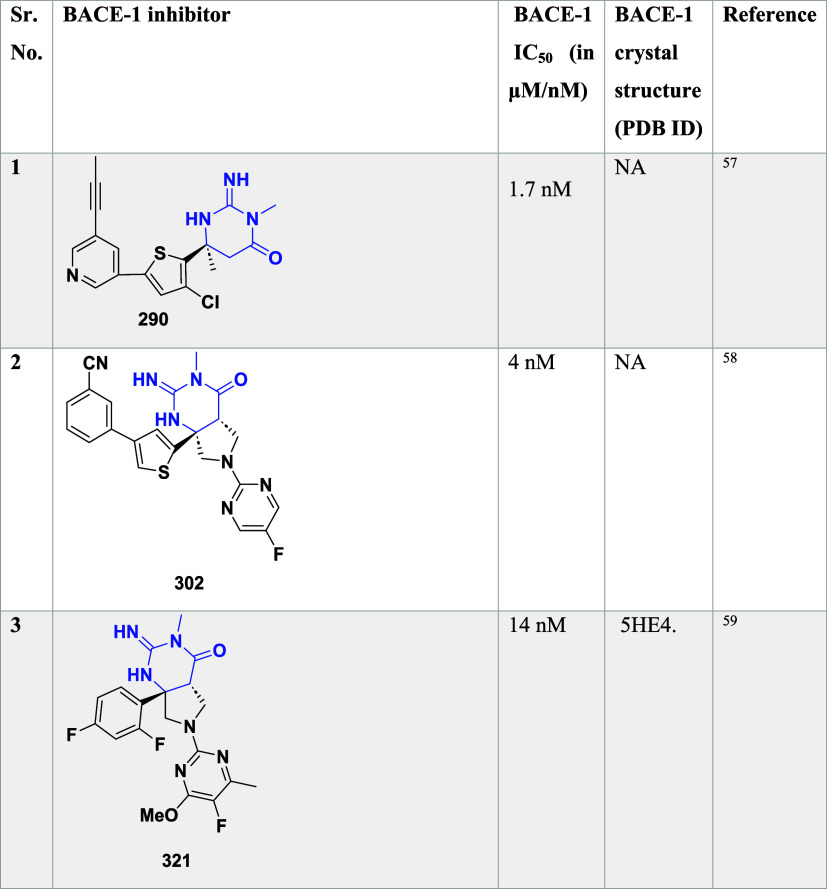
BACE-1 Inhibitors, the Co-Crystal Structure of Bace-1 Enzyme, and Their IC_50_ Values

Mandal et al. reported the synthesis of novel bicyclic iminopyrimidinones as a β-secretase inhibitor.^58^ Initially, the Suzuki coupling reaction of **291** with various boronic acids to afford the amide-substituted iminomyridines **293**, which were then treated with tetrabutylammonium fluoride (TBAF) to generate intermediate **292**. The coupling of **292** with trifluoracetic acid yielded amide-substituted iminopyrimidinones **293** (Scheme 46).

**Scheme 46:**
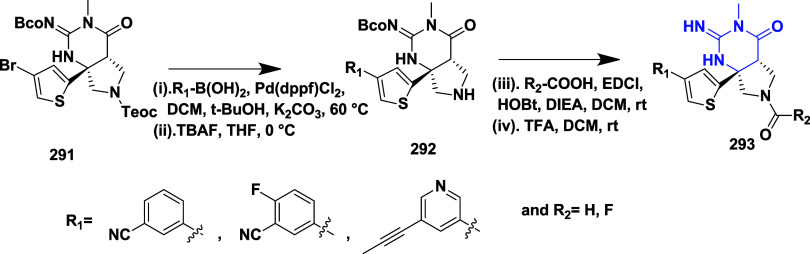
Synthesis of Amide-Substituted Iminopyrimidinones **293**

First, the debromination of **294**, followed by treatment with *N*-bromosuccinamide (NBS), resulted in intermediate **295**. This intermediate was then converted into free amine **296** via Suzuki coupling reaction of **295** with (3-cyano-4-fluorophenyl) boronic acid, catalyzed by dichloro [1,1′-bis­(diphenylphosphine)-ferrocene] palladium in the presence of potassium carbonate. Arylation of the pyrrolidine core of **296** mediated by palladium and acid deprotection, yielding analog iminopyrimidinones **297** ^58^ (Scheme 47).

**Scheme 47:**
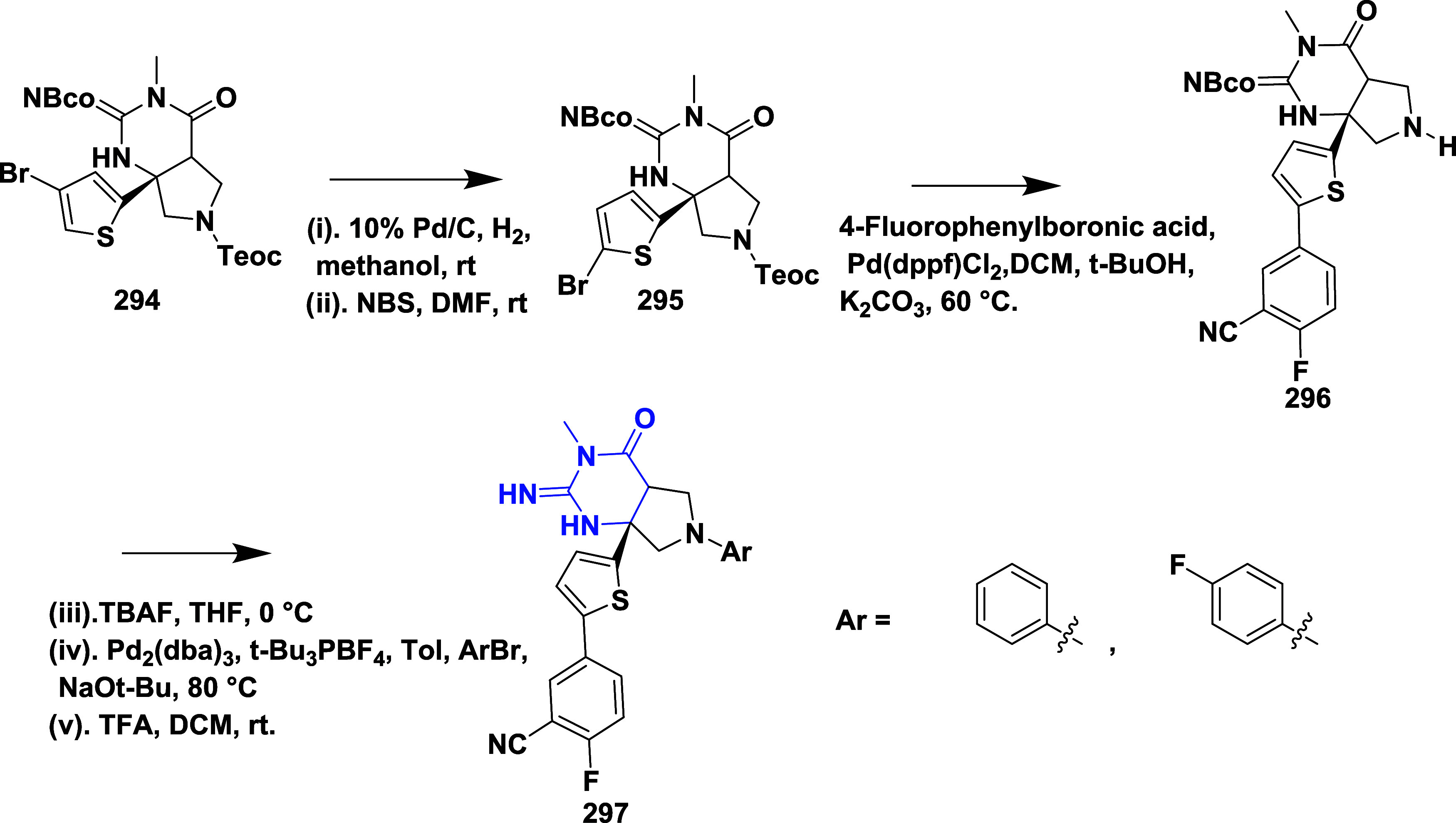
Synthesis of *N*-Phenyl Analogues of Iminopyrimidinones **297**

Compound **299** was synthesized by coupling an appropriate boronic acid with copper acetate. However, **301** and **302** were synthesized by *N*-arylation mediated by palladium with the suitable pyridine or pyrimidine halide, followed by acid-mediated deprotection of the imine group, yielding the final targeted iminopyrimidinones (Scheme 48). SAR studies on fused bicyclic iminopyrimidinones aimed to lock the biaryl group in a pseudoaxial conformation to maximize interactions within the S1 to S3 subsites of BACE-1. Introducing a fused pyrrolidine ring improved potency by stabilizing the binding conformation and engaging the S2′ and S2″ pockets. Amide-substituted compounds, such as **293**, showed potent BACE-1 inhibition with a *K_i_* of 2 nM, but exhibited poor pharmacokinetic properties. Replacing the amide with *N*-aryl groups maintained high potency and significantly enhanced oral bioavailability. The optimized compound **302** (Table 5), featuring a fluoropyrimidine moiety, demonstrated potent inhibition (*K_i_* = 4 nM), good brain penetration, and dose-dependent CNS Aβ40 lowering in rats, emphasizing the importance of conformational control and physicochemical tuning in BACE-1 inhibitor development.

**Scheme 48:**
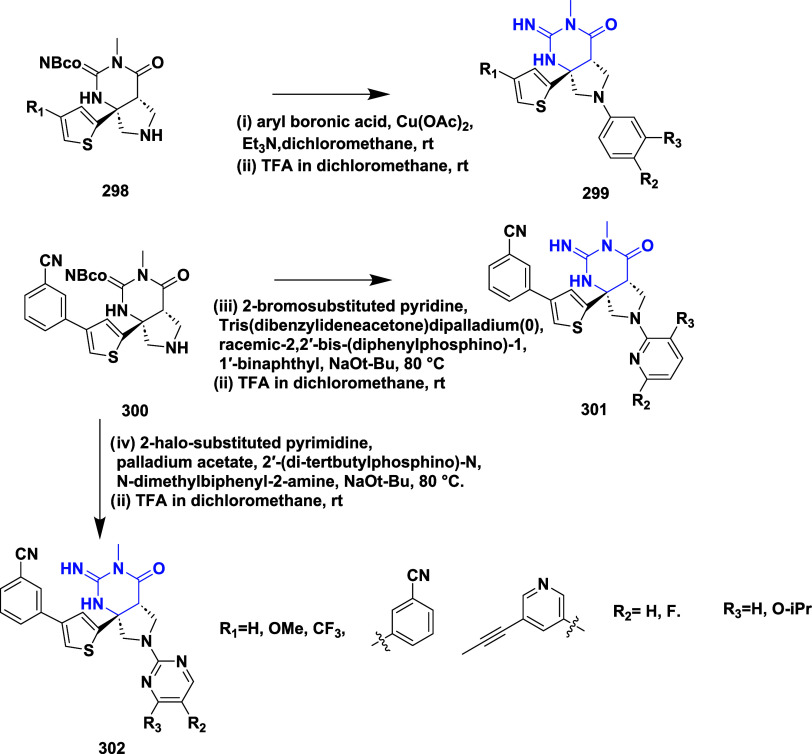
Synthesis of *N*-Phenyl 288, *N*-Pyridinyl **301**, *N*-Pyrimidinyl **302** Analogs of Iminopyrimidinones

Mandal and co-workers reported the synthesis of substituted iminopyrimidinones as a BACE-1 inhibitor.^59^ The synthesis involved the debromination of bromothiophene **303** under controlled hydrogenation conditions to yield intermediate **304**. Further, it undergoes bromination in the presence of *N*-bromosuccinamide, resulting in an isomeric bromothiophene **305**. The cyanation of **305** using a zinc/zinc chloride under palladium­(II) chloride (1,1′-bis (diphenylphosphine) ferrocene) and the acidic deprotection of the Teoc group using a tributyl ammonium fluoride (TBAF) yields pyrrolidine **306**. The intermediate **304** was chlorinated with *N*-chlorosuccinimide to generate an intermediate **309**, then treated with TBAF for deprotection. The compounds **307** and **310** are obtained from **306** and **309**, respectively, under palladium-catalyzed *N*-arylation with various heteroaryl halides and treated with TFA (Scheme 49).

**Scheme 49:**
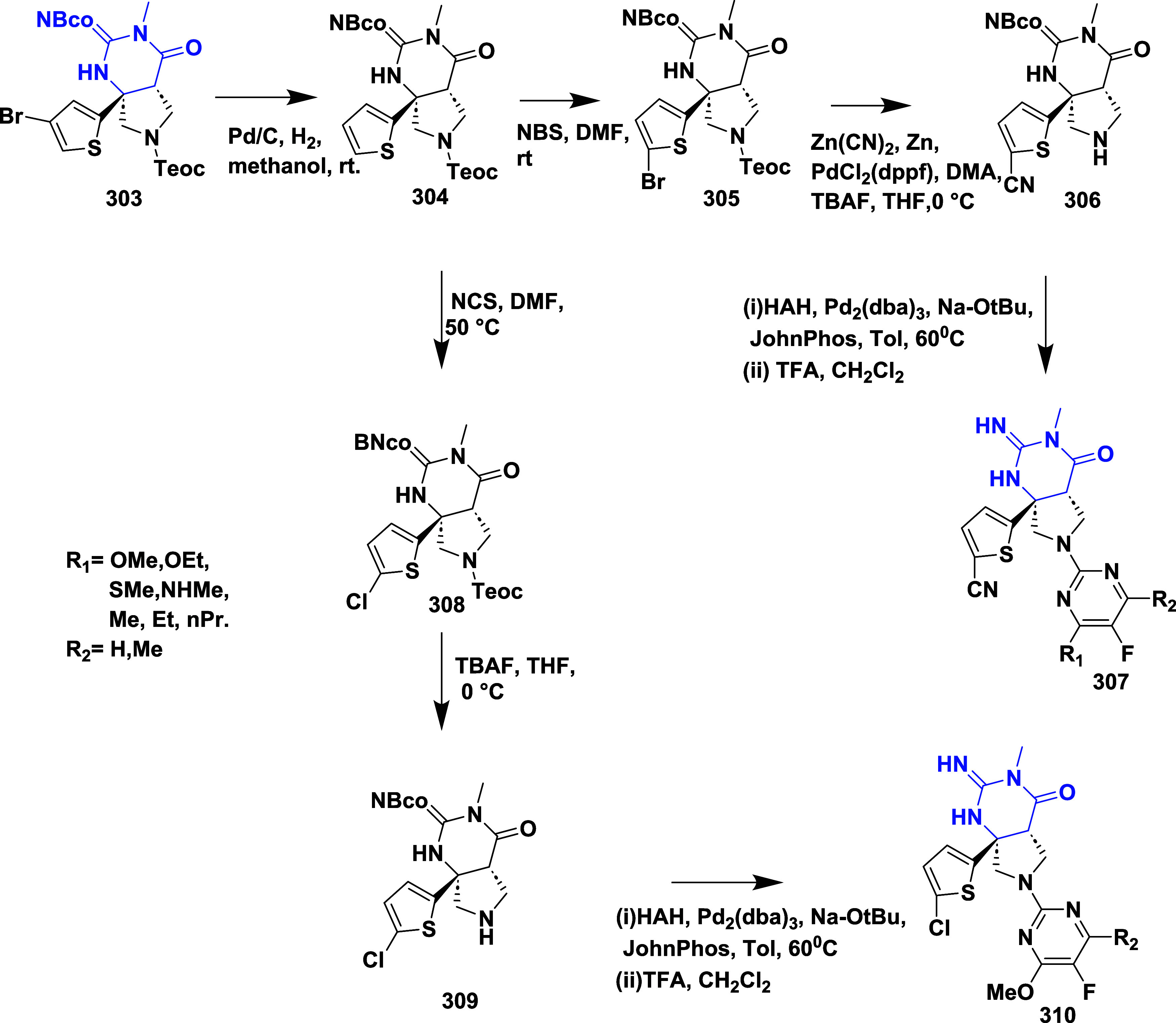
Synthesis of Thiophene Analogs of Iminopyrimidinones **307** and **310**

The condensation of 2-(2,4-fluorophenyl) acetonitrile **311** with aq. Glyoxylic acid, yielding compound **312**. The hydrolysis of the nitrile moiety **312** using a formic and sulfuric acid mixture to produce the anhydride **313**. Then, **313** undergoes dipolar cycloaddition with *N*-benzyl-1-methoxy-*N*-((trimethylsilyl)-methyl) methanamine **314** to yield methyl ester **315**. DPPA mediated Curtius rearrangement of a carboxylic acid moiety of **316** converted to amines, yielding an intermediate **317**, which was coupled with *t*-butyl-*N*-((methylamino)-chloromethyl) carbamate to form iminopyrimidinones with Boc protected **318**. Separate the enantiomer and provide a desired compound **319**, followed by benzyl group removal and coupling with pyrrolidine **320**, yielding 2,4-difluoro compound **321** by the treatment with trifluoracetic acid with a yield of 34%^59^ (Scheme 50). The SAR strategy began with the modification of a potent lead compound featuring a fused pyrrolidine iminopyrimidinone core, along with adjustments to the S3-binding cyanophenyl group and the S2′/S2″ pyrimidine substituent, led to the development of analogs such as **307** and **308**, which exhibited enhanced potency and reduced CYP3A4 inhibition. Substituent changes on the pyrimidine ring showed the region could accommodate minor modifications, while bulkier or highly polar groups diminished activity. Enhancing interactions in the S1 pocket through replacement of the thienyl group with fluorinated phenyl rings led to compound **321** (MBi-4) (Table 5), a standout molecule with a *K_i_* of 5 nM, IC_50_ of 14 nM, brain-to-plasma ratio of 1.1, and over 400-fold selectivity against CatD. Compound **321** also produced significant Aβ40 lowering in rodent and primate models, making it a promising brain-penetrant BACE-1 inhibitor with favorable safety and pharmacodynamic properties. Similarly, fluorophenyl analogs of iminopyrimidinones 333 were prepared using the starting materials **322** and **323** (Scheme 51).

**Scheme 50:**
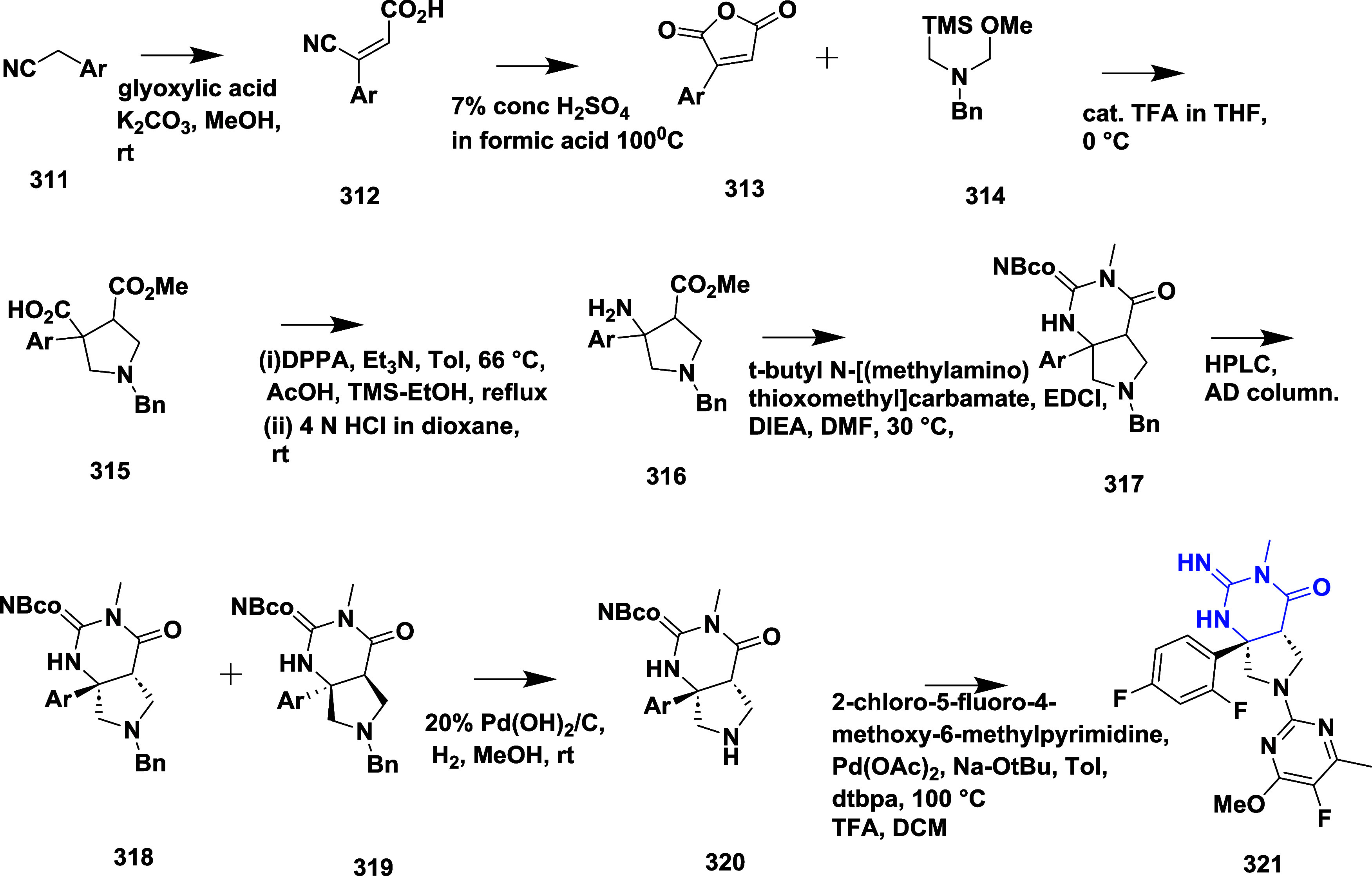
Synthesis of 2,4-Difluorophenyl Analogue of Iminopyrimidinones **321**

**Scheme 51:**
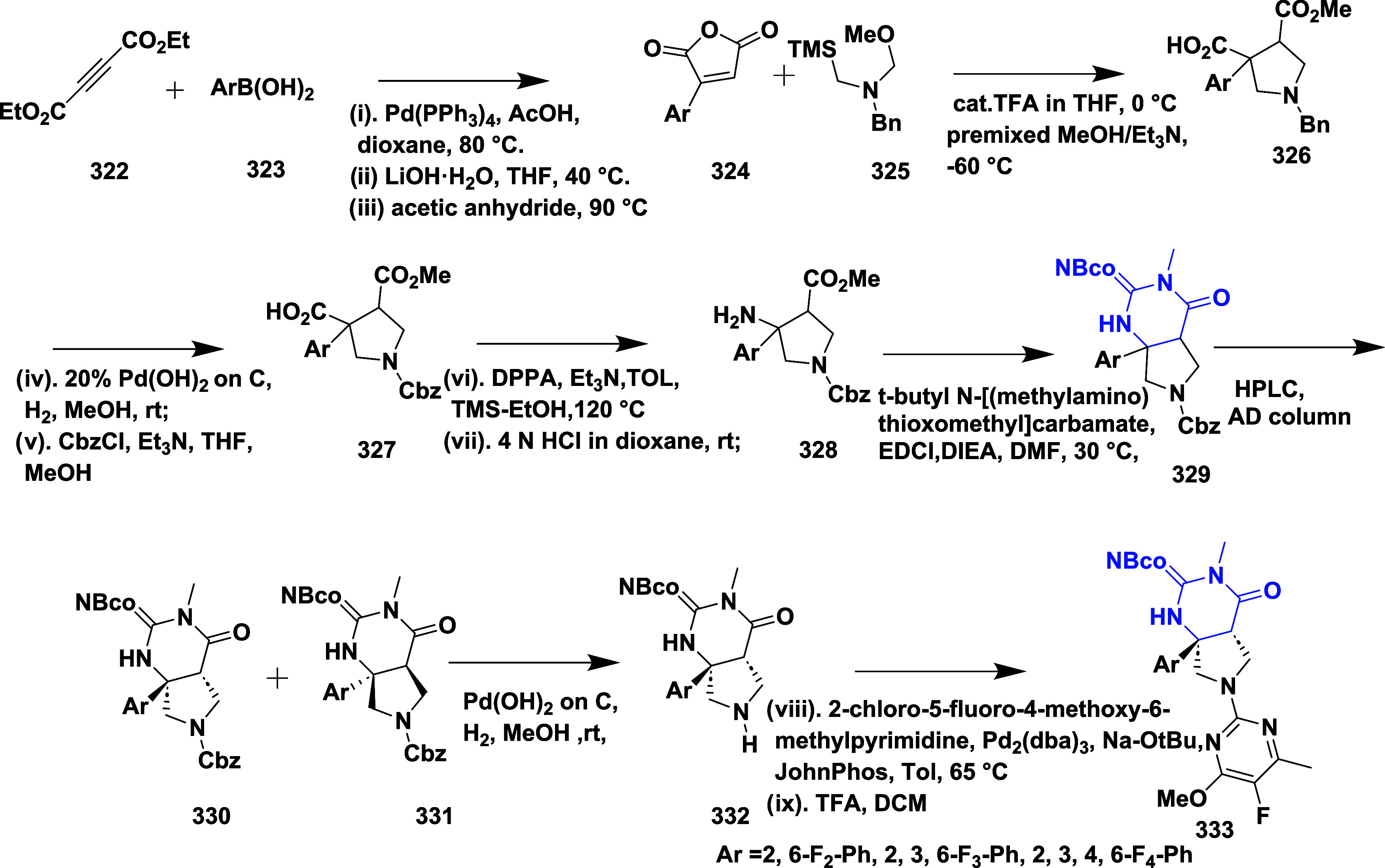
Synthesis of Fluorophenyl Analogue of Iminopyrimidinones **333**

## Synthesis of Other BACE-1 Inhibitors

Ortega et al. reported that the synthesis of aryl quinones as BACE1 inhibitors for AD,^60^ which involved Pd (II) catalyzed coupling reaction of 2-aryl-1,4-benzoquinones **334** with aryl boronic acids leading to the formation of two regioisomers: 2,5-diaryl-1,4-benzoquinones **335** and 2,6-diaryl-1,4-benzoquinones **336**. The synthesized compounds were obtained in good yields, ranging from 60 to 90% (Scheme 52). The SAR studies highlighted that the activity of arylquinones against BACE-1, Aβ aggregation, and fibril destabilization was closely linked to the quinone core’s substitution pattern and redox state. Compound 8-hydroxy-2-phenylnaphthalene-1,4-dione was the only naphthoquinone to exhibit all three activities, while **335** (Ar_1_ = Ph and Ar_2_ = p-CF_3_–C_6_H_4_) (Table 6) emerged as the most potent BACE-1 inhibitor among benzoquinones with an IC_50_ value of 6.52 μM. Free hydroxyl groups proved critical, as their acetylation led to a significant loss of activity, likely due to the disruption of hydrogen bonding, and specific substitutions (e.g., methoxy, trifluoromethyl) influenced both activity and cytotoxicity. Reduction to hydroquinones generally impaired aggregation inhibition but could preserve BACE-1 activity in some cases.

**Scheme 52:**
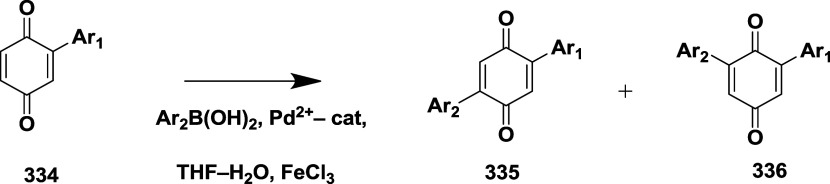
Synthesis of Aryl Quinones **335** and **336**

**Table 6:**
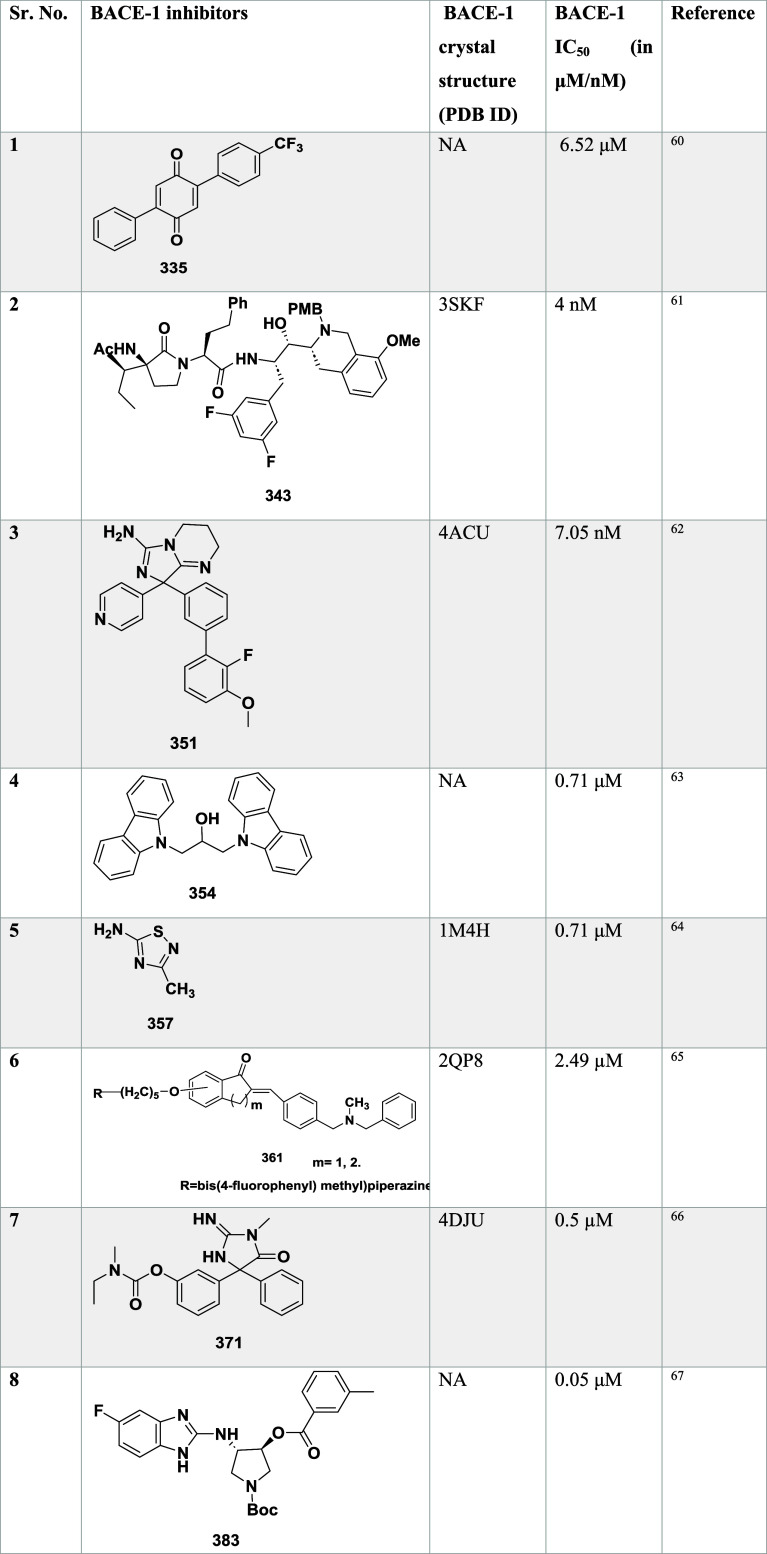

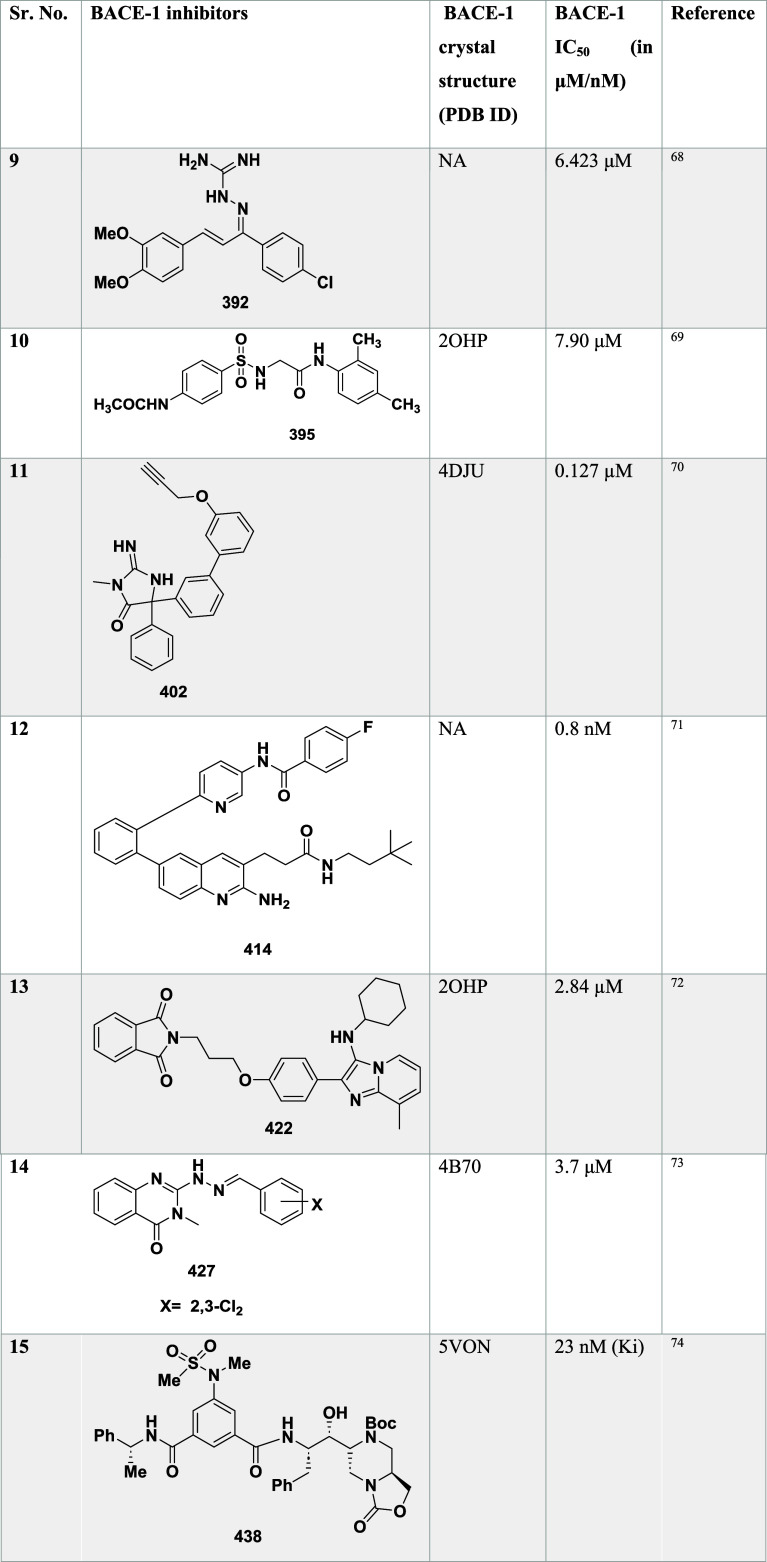

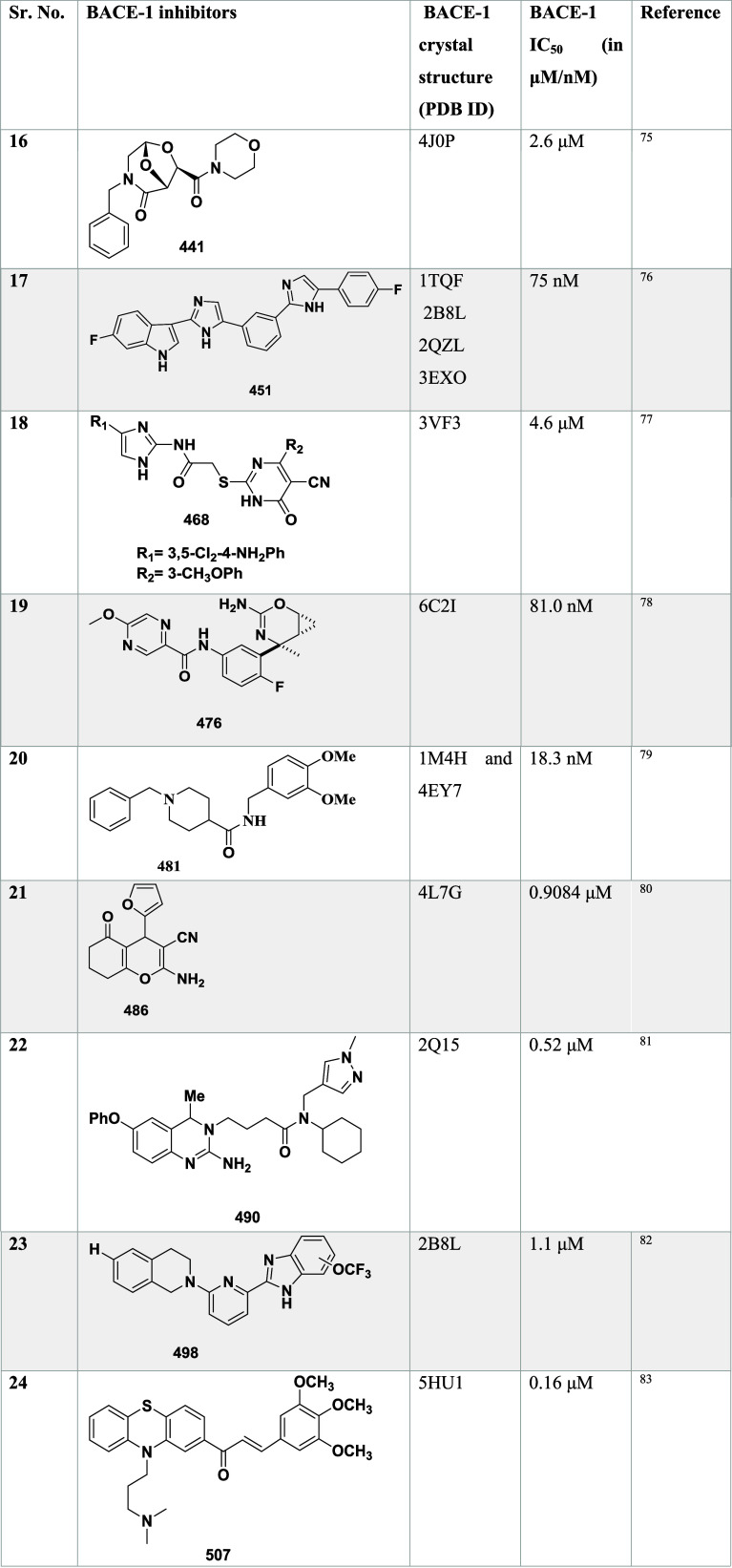

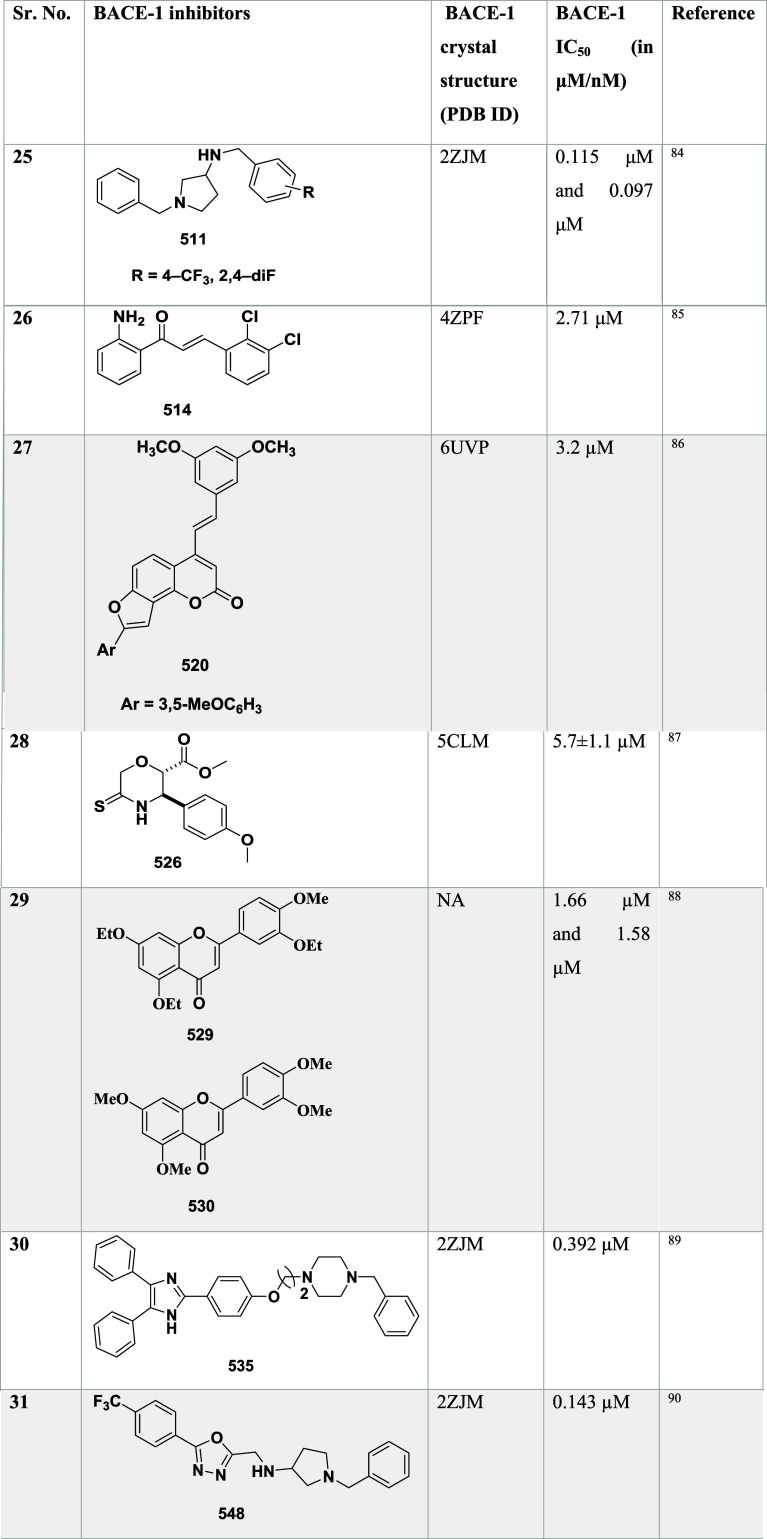

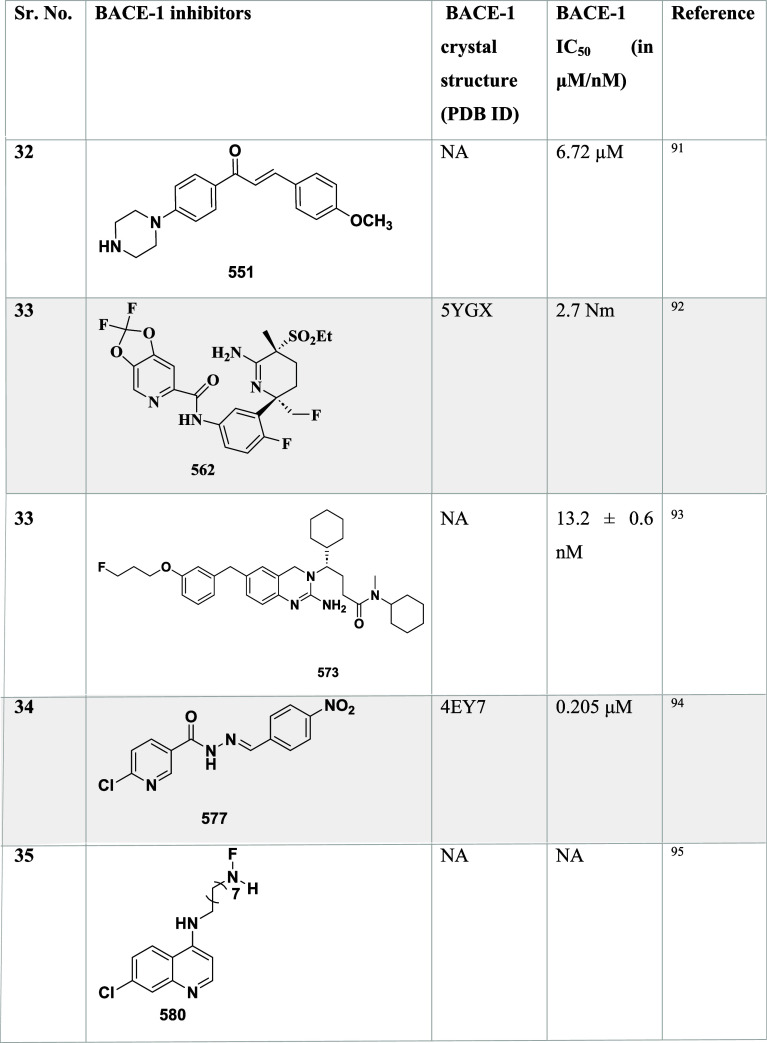
BACE-1 Inhibitors, the Co-Crystal Structure of Bace-1 Enzyme, and Their IC_50_/*K_i_* Values

Thompson et al. synthesized cyclic diamino propane analogs as BACE-1 inhibitors,^61^ which involved the aldol condensation between acyl oxazolidine **337** and an aldehyde **338**, yielding an aldol adduct **339**, followed by chiral auxiliary removal and Curti’s rearrangement, which led to the formation of acyclic oxazolidine **341**. The Boc group on compounds **341** was converted to *p*-methoxybenzyl (PMB), followed by saponification, which afforded intermediate **342** with good yield. The amide coupling of lactam headgroup **342**, followed by acidic deprotection, provides cyclic diamino propane **343**. The synthesized compounds were isolated in good yields ranging from 85 to 99% (Scheme 53). The structure–activity relationship (SAR) investigations within this series of BACE-1 inhibitors centered on refining cyclic diaminopropane scaffolds to enhance potency while minimizing peptide-like features. Early structural modifications involved constraining the P2–P3 region through lactam incorporation and replacing the traditional hydroxyethylene isostere with a diaminopropane motif, yielding highly active compounds such as BMS-599240 (IC_50_ = 5 nM). Continued optimization introduced tetrahydroisoquinoline (THIQ) derivatives, where the stereochemistry at the THIQ core played a crucial role in determining inhibitory strength. For instance, compound **343** (X = OMe, Table 6) outperformed its diastereomer. Activity was further improved by incorporating groups like methoxy and isobutyl on the lactam ring. Additionally, the addition of 4-sulfone substituents on pyrrolidine rings led to enhanced cellular efficacy, although in vivo brain activity was limited to P-glycoprotein (P-gp) knockout models, underscoring the importance of efflux transport in central nervous system penetration.

**Scheme 53:**
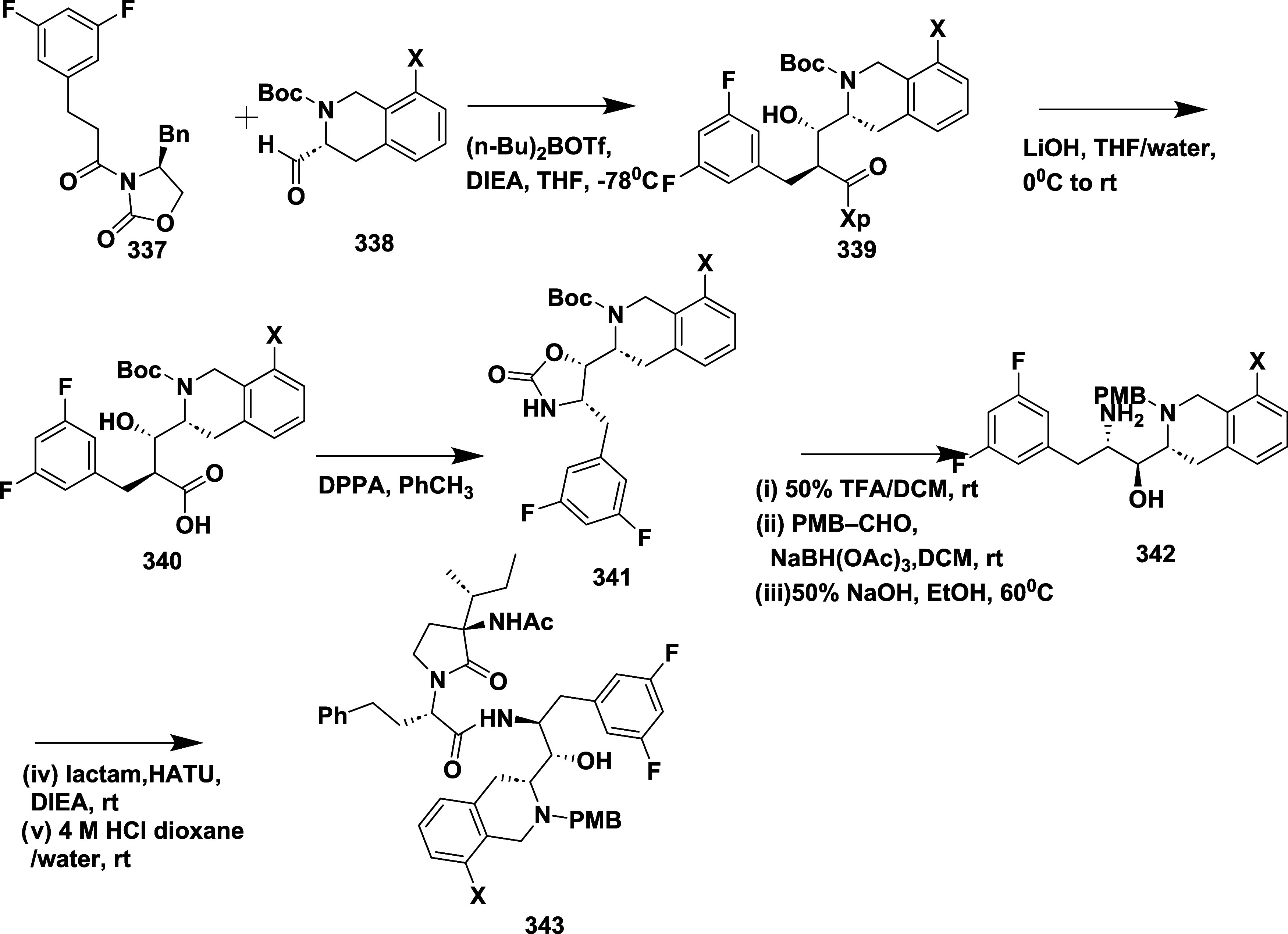
Synthesis of Cyclic Diamino Propane **343**

Swahn et al. reported the synthesis of amino imidazoles as a BACE-1 inhibitor.^62^ The synthesis begins with a reaction of 1,3-dibromobenzene **344** with butyl lithium, followed by 4-cyanopyridine and sodium borohydride reduction, resulting in an amine **345**. The amine is converted into isothiocyanate **346** via a reaction of **345** with thiocarbonyldiimidazole in dichloromethane. This compound then reacted with potassium *tert*-butoxide and carbon disulfide in THF at a low temperature to yield thiazolidine-2,5-dithionite derivatives **347**. Thiazolidine-2,5-dithionite derivatives **347** react with propylenediamine to produce 3,4,7,8-tetrahydroimidazo­[1,5-*a*] pyrimidine-6-thione derivative **348**. This derivative was then treated with ammonia and *tert*-butylhydroperoxide in methanol to yield the corresponding amine, 2,3,4,8-tetrahydroimidazo­[1,5-*a*]­pyrimidine-6-amine (THIP) derivative **349**. In the last stage, compound **351** was synthesized by introducing the 2-fluoro-3-methoxyphenyl group via a palladium-catalyzed microwave-assisted Suzuki coupling reaction. The synthesized compounds were isolated in good yields of 45% (Scheme 54). Compound **351** showed an IC_50_ value of 7.05 nM (Table 6), making it a potent compound, but it showed a low Caco-2 value, which resulted in poor blood-brain barrier penetration. The SAR analysis revealed a strategic progression from dihydroisocytosine to aminohydantoin scaffolds, eventually culminating in the design of bicyclic aminoimidazole derivatives to enhance both inhibitory potency and membrane permeability. Incorporation of difluoro groups onto the tetrahydroimidazopyrimidine framework led to a reduction in basicity (as indicated by a lower p*K*_a_) and notably enhanced Caco-2 permeability. Replacing biaryl side chains with more linear alkyl groups effectively decreased efflux liability and improved in vivo profiles. Moreover, coadministration with a dual P-gp/BCRP inhibitor successfully facilitated Aβ40 lowering in both plasma and brain tissues.

**Scheme 54:**
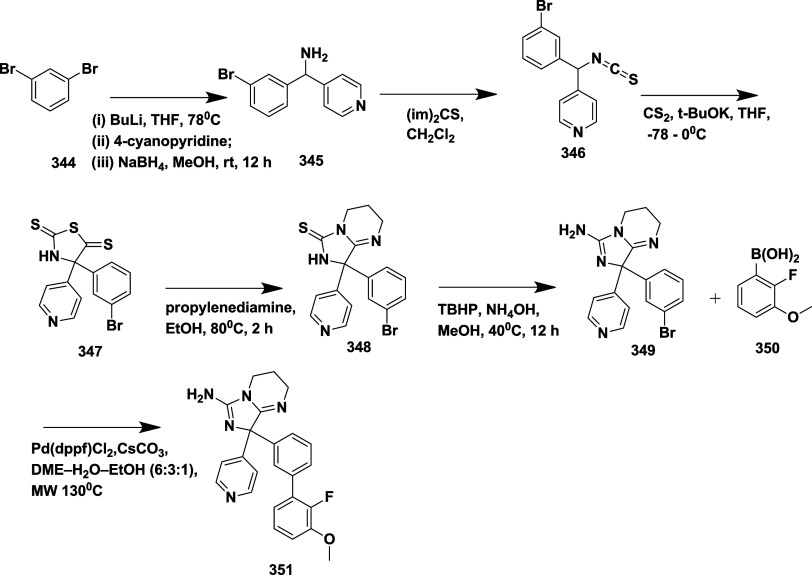
Synthesis of 2-Fluoro-3-methoxyphenyl Aminoindazole **351**

Kumar et al. synthesized a new 1,2-disubstituted 2-propanol as a BACE-1 inhibitor.^63^ The nucleophilic ring opening of epoxide (9­(oxiran-2-ylmethyl)-9*H*-carbazole) **352** in the presence of napthalene-2-thiol **353** yielded 1,3-disubstituted-2-propanol derivative **354** with good yield (52%) under reflux (Scheme 55). SAR findings demonstrated that potent BACE-1 inhibition was achieved by combining a naphthyl or carbazole core with an extended aromatic fragment such as carbazole or 2-phenyl-indole, enabling engagement with multiple subpockets of the enzyme. High potency depended on a short linker containing four methylene units and a single hydroxyl group, which served to mimic the aspartyl protease transition state. Among the series, compound E7T18 showed promising activity (IC_50_ = 4.93 μM), while further structural refinement produced bis-carbazole derivative **354**, exhibiting an IC_50_ of 0.71 μM and over 422-fold selectivity versus cathepsin D. These top candidates also showed favorable blood–brain barrier penetration predictions, underscoring their potential as CNS-targeted agents.

**Scheme 55:**
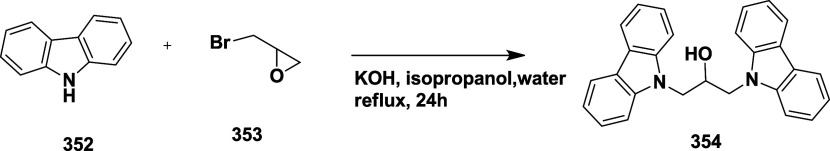
Synthesis of Bis-carbazole **354**

Gurjar et al. reported the synthesis of novel thiadiazole urea analogs as a BACE-1 inhibitor.^64^ 3-substituted-1,2,4-thiadiazol-5-amine **357** was synthesized from thiourea 355, reacted with dimethyl sulfate 356, yielding an intermediate **358**, which undergoes cyclization with sodium thiocyanate (NaSCN), followed by bromination in the presence of triethylamine. The condensation of **357** with phenyl compounds resulted in a series of phenyl-1,2,4-thiadiazolylurea analogs **359** (Scheme 56). The synthesized compounds were isolated in good yields ranging from 38 to 86%. SAR evaluations indicated that 3-substituted-1,2,4-thiadiazol-5-amines demonstrated notable BACE-1 inhibitory potential, with compound **357** (IC_50_ = 5.96 μM, Table 6) standing out due to its strong interactions with the catalytic dyad (Asp32 and Asp228) and its favorable pharmacokinetic profile. Variations at the R position revealed that short alkylthio substituents such as methylthio and ethylthio enhanced binding affinity, while extending the chain beyond six carbons reduced interaction efficiency. Modifying the amine core into phenyl urea frameworks typically led to diminished activity; however, select derivatives like **359s** maintained moderate inhibitory effects, attributed to their capacity to form multiple hydrogen bonds with residues such as Gln73 and Thr231.

**Scheme 56:**
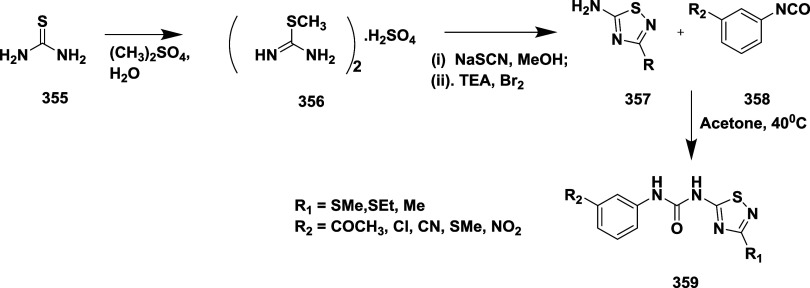
Synthesis of Phenyl-1,2,4-thiadiazolylurea Analogues **359**

Rampa et al. reported the synthesis of new (*E*)-2-(4-((benzyl­(methyl)­amino) methyl) benzylidene)-6-hydroxy-2,3-dihydro-1*H*-inden-1-one derivatives as a β-site APP cleaving enzyme inhibitor.^65^ Which involves the nucleophilic addition of 2- {4-[(benzyl methylamino)­methyl] benzylidene} (5-iodopentyloxy)-2,3-dihydro-1*H*-indan-1-one (*m* = 1) and [(benzyl methyl amino)­methyl]­benzylidene-6-(5-iodopentyloxy) 3,4-dihydro-2*H*-naphthalen-1-one­(*m* = 2) with amines offered a desired target compounds **361** (Scheme 57). The synthesized compounds were isolated with 12 to 58% yields. SAR analysis demonstrated that enlarging the amine substituent on the indanone framework markedly improved BACE-1 inhibition while diminishing AChE activity. The original compound, although a potent AChE inhibitor, showed minimal activity against BACE-1; however, substituting the terminal piperidine with bulkier amines such as bis­(4-fluorophenyl)­methyl piperazine (compound **361**, Table 6) led to significant BACE-1 inhibition (IC_50_ = 2.49 μM). Incorporating fluorinated aromatic groups enhanced target binding, likely through improved protein–ligand interactions and favorable molecular orientation. Notably, compound **361** exhibited no signs of neurotoxicity, in contrast to the parent molecule, indicating a superior pharmacological profile. These findings suggest that amine bulk and fluorination are pivotal in redirecting activity from AChE toward selective BACE-1 inhibition.

**Scheme 57:**
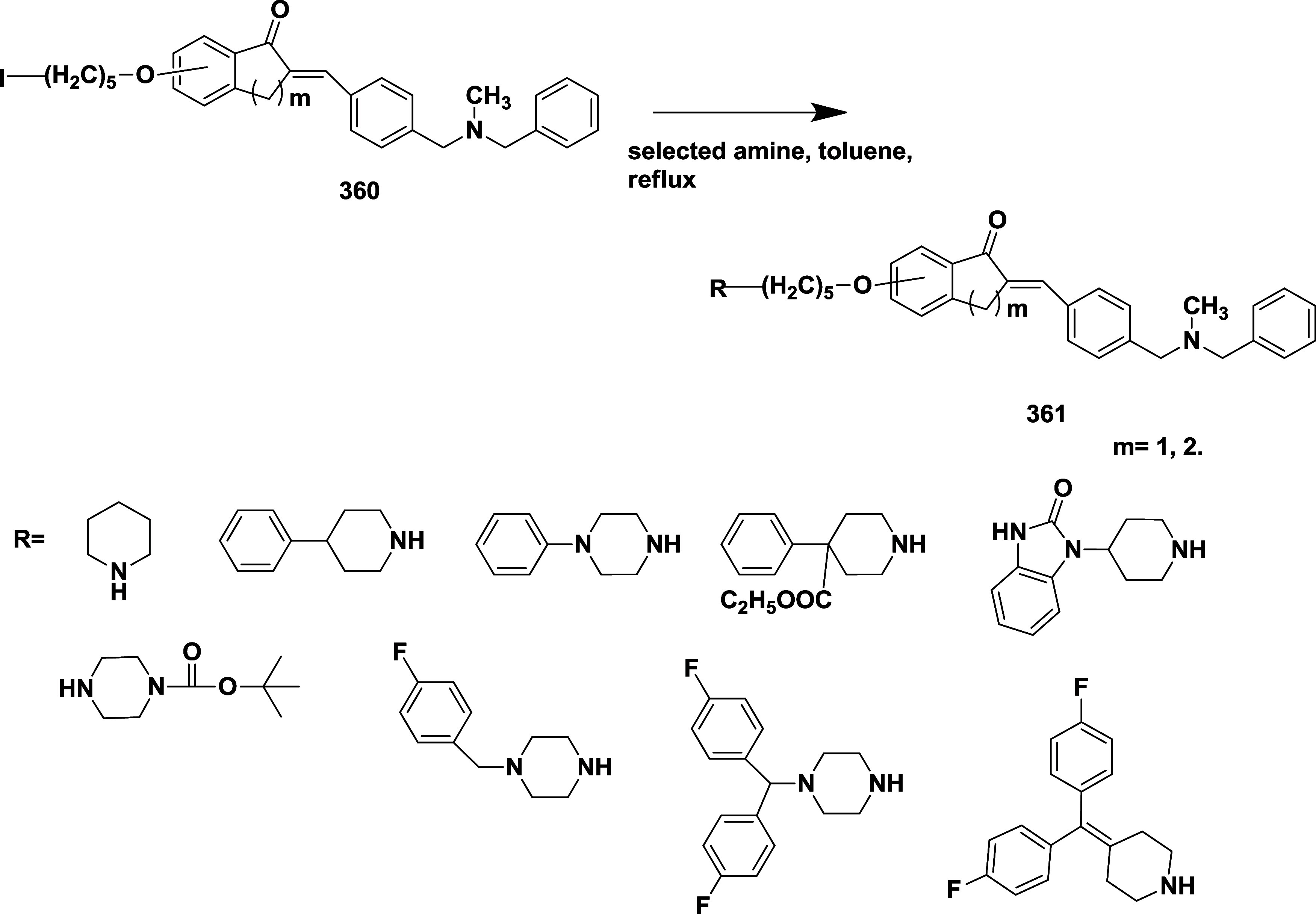
Synthesis of Indanone Derivatives **361**

Liu et al. reported the synthesis of acyl guanidines as a β-site APP cleaving enzyme inhibitor.^66^ First, protecting the hydroxy group of 3-hydroxybenzoic acid **362** by reaction with methyl iodide in basic medium affords intermediate **363**. The hydrolysis of **363** with NaOH yields intermediate **364**. The 3-methoxybenzoyl chloride **365** was made from **364** by reacting with thionyl chloride, followed by Friedel–Crafts reaction with benzene, catalyzed by aluminum trichloride. Acetic acid-mediated deprotection of the methyl group under reflux yielded intermediate **366**. In the reaction of **366** with ethyl­(methyl)­carbamic chloride, the phenol group was changed into a phenol ester group, yielding **367**. The refluxing compound **367** with sodium cyanate and ammonium carbonate in ethanol/water yielded **368**. The methylation of **368** with methyl iodide in acetone and potassium carbonate produced target compound **369**. The carbonyl group **369** was converted to thiocarbonyl using Lawesson’s reagent to get **370**. Finally, the thiocarbonyl (CS) group of compounds **370** was transformed into a cyano group (CN) via treatment with ammonium hydroxide and *tert*-butyl hydroperoxide, producing **371** (Scheme 58).

**Scheme 58:**
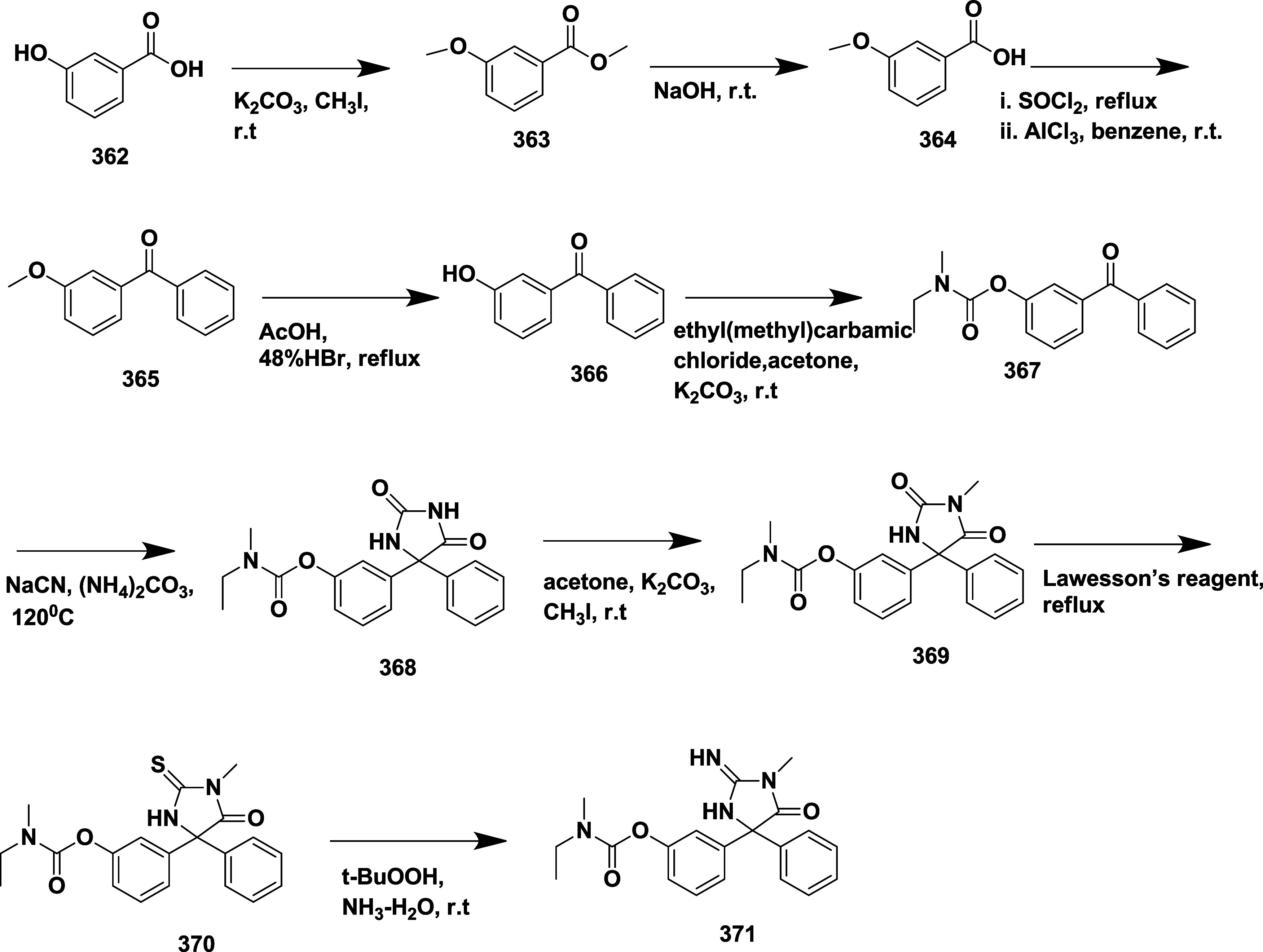
Synthesis of Iminomethylphenylimidazolidinyl-phenyl-carbamate **371**

Scheme 59 depicts the synthesis of designed compounds **378**.^66^ Copper iodide/dichlorobis­(triphenylphosphine)­palladium­(II)/TEA catalyzed the reaction of 3-iodophenol with ethynyltrimethylsilane, yielding **373**. The cross-coupling of **373** with 1-bromo-3-fluorobenzene at 80 °C catalyzed by PdCl_2_(PPh_3_)_2_/TBAF yielded **375**. The oxidation of the ethynyl group of **375** with potassium permanganate in magnesium sulfate and sodium carbonate yielded **376**. After that, **376** and 1-methyl guanidine hydrochloride were cyclized and underwent rearrangement at 90 °C, yielding **377**. Subsequently, the reaction **377** with ethyl (methyl) carbamic chloride yielded **378** (Scheme 59). The yield of potent compound **371** was around 21%. This compound showed a 92.65% inhibition rate against BACE-1 at 10 μmol/L with the IC_50_ value of 0.5 μM (Table 6). The SAR study was initiated with 2-imino-3-methyl-5,5-diphenylimidazolidin-4-one, a modestly active yet brain-penetrant BACE-1 inhibitor (IC_50_ = 7.1 μM), and focused on structural modifications to extend molecular interactions into the S3 pocket. Enhancing this interaction, compound **371** retained the guanidine group. It introduced a phenyl ester moiety directed at the S3 region, resulting in a 14-fold increase in inhibitory potency, and substituting the guanidine functionality with urea or thiourea significantly diminished activity, emphasizing its essential role in forming hydrogen bonds with key catalytic residues Asp32 and Asp228. Additional changes to the second phenyl ring, as seen in compound **378**, led to reduced efficacy, likely due to steric clashes or unfavorable conformations. These results underline the importance of maintaining the guanidine scaffold while strategically extending into the S3 pocket to optimize potency and CNS accessibility.

**Scheme 59:**
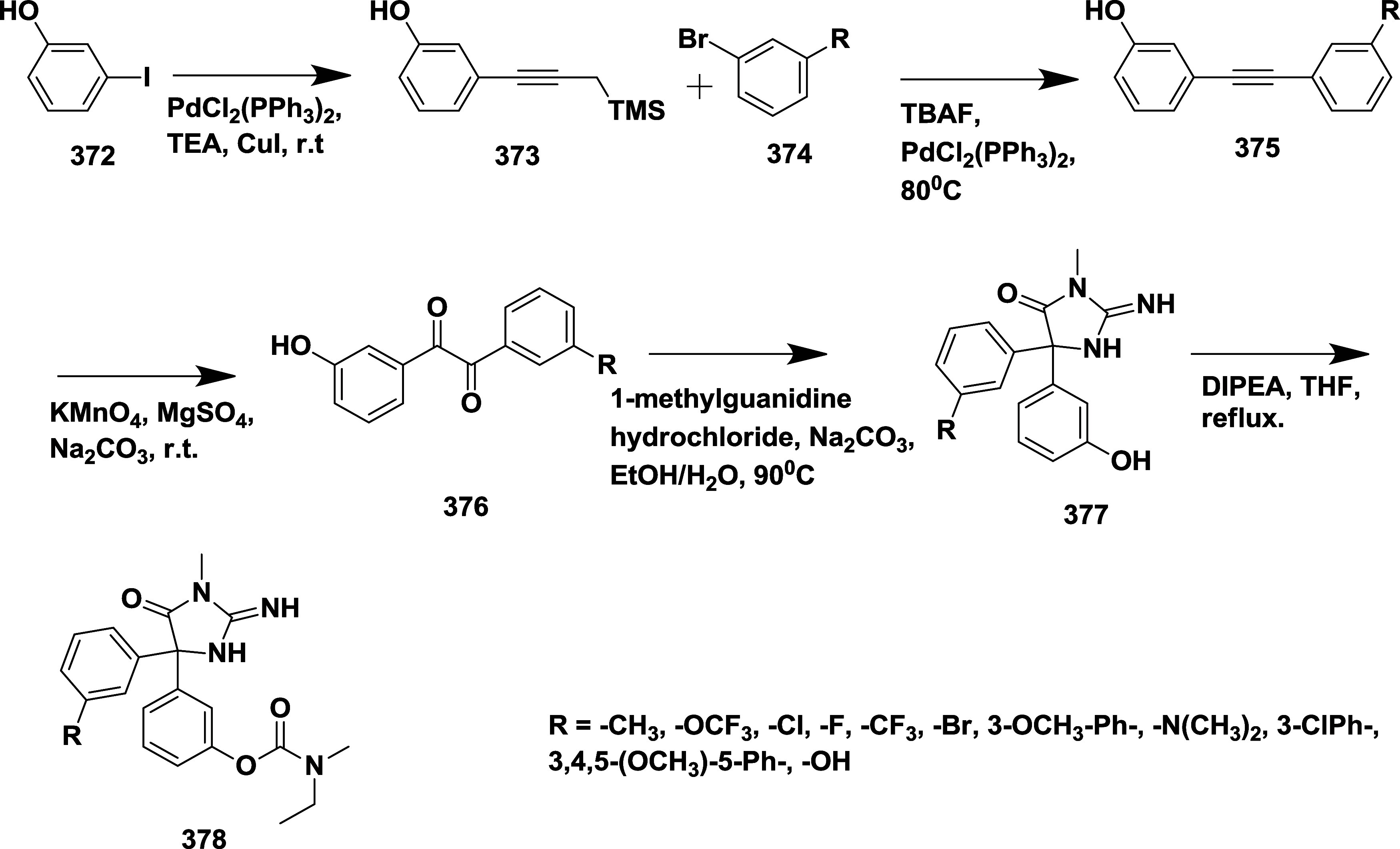
Synthesis of 3-(2-Imino-1-methyl-5-oxo-4-phenylimidazolidin-4-yl)­phenyl Ethyl­(methyl)­carbamate Derivatives **378**

Tran et al. reported the synthesis of a novel (3*S*,4*S*)-4-aminopyrrolidine-3-ol derivative as a β-secretase inhibitor.^67^ Synthesis involves Jacobsen’s chiral (salen) chromium­(III) complex-induced ring opening of epoxide **379** to give chiral azido alcohol **380**. It, coupled with various benzoic acids, azido alcohol 380, afforded esters, followed by palladium-catalyzed reduction of the amino group, producing aminobenzoates **381**. The treatment of di-2-pyridinyl thiocarbamate with **381** produced isothiocyanates **382**, which were then coupled with 1,2-diaminobenzene and cyclized to afford benzimidazole derivative **383**. The desired compound is (3*S*,4*S*)-4-((1*H*-benzo [d]-imidazoles-2-yl) amino) pyrrolidine-3-yl benzoate derivatives **384**, obtained from **383**, by Boc deprotection of **384**, followed by acylation (Scheme 60). Similarly, benzyl ether-linked pyrrolidine derivatives **388** were produced from azido benzyl ether **386** (Scheme 61). The SAR findings revealed that substituting the piperidine ring in the lead 3-amino-4-aryl-piperidine and 4-arylpiperazinylpiperidine series with a (3*S*,4*S*)-pyrrolidine-3-ol scaffold led to a marked increase in BACE-1 inhibitory activity, exemplified by compound **383** (IC_50_ = 0.05 μM, Table 6). Incorporation of functional groups such as 5-fluorobenzimidazole and aryl esters further enhanced binding through key interactions with Asp228, Gln73, and Tyr198. On the other hand, modifications like Boc group removal or sulfonyl/acylation at the pyrrolidine nitrogen diminished potency, likely by disrupting the optimal binding conformation. Notably, compound **388** exhibited more vigorous cellular activity despite weaker enzymatic inhibition, suggesting that permeability plays a significant role in overall bioactivity

**Scheme 60:**
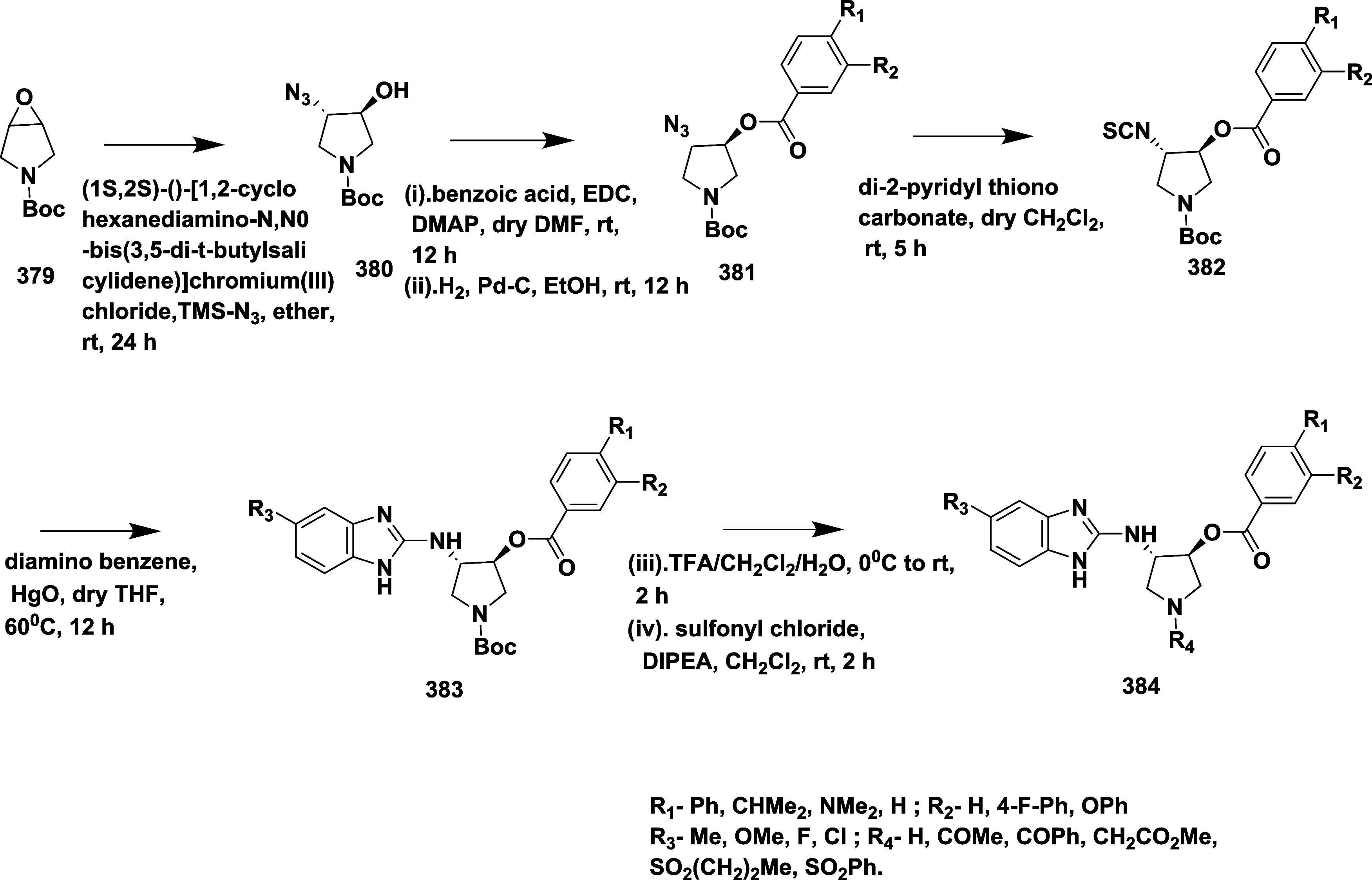
Synthesis of Substituted Pyrrolidine-3-yl Benzoate Derivatives **384**

**Scheme 61:**
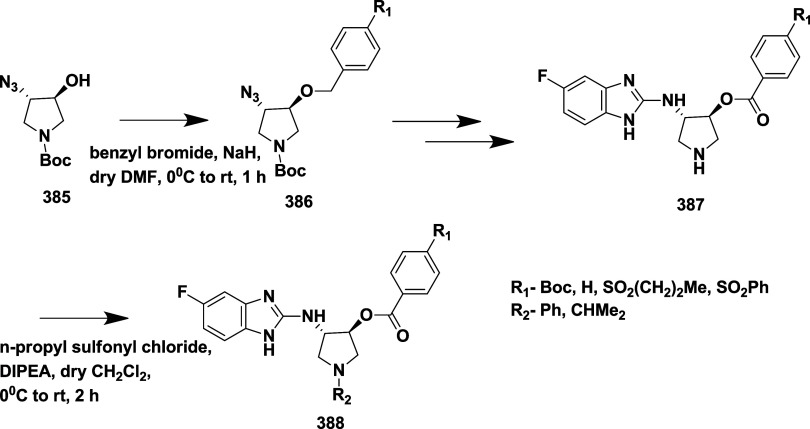
Synthesis of Benzyl Ether-Linked Pyrrolidine Derivatives **388**

Jain et al. reported the synthesis of allylidene hydrazine carboxamide as a novel β-secretase inhibitor.^68^ The synthesis involves the condensation of substituted aromatic aldehydes **389** with acetophenones **390** in the presence of a base, resulting in benzylidiene acetophenones **391**. It then reacts with aminoguanidine hydrochloride, producing the desired product, allylidenehydrazine carboxamide **392**, by eliminating water as a side product (Scheme 62). The synthesized compounds were isolated with yields ranging from 21 to 75%. SAR analysis revealed that the aminoguanidine group is crucial for forming hydrogen bonds with the key catalytic residues Asp32 and Asp228. At the same time, the two aromatic rings contribute significantly through hydrophobic interactions within the S1 and S3 pockets. Enhancements in activity were observed when ring A substitutions facilitated interactions with residues like Thr232 and Gly11 in the S3 pocket, and electron-withdrawing groups on ring B, such as para-chloro or para-nitro, strengthened π–π stacking with Tyr71. Among the tested compounds, derivative **392**, featuring a para-chloro group on ring B and meta, para-dimethoxy substitution on ring A, demonstrated the highest potency with an IC_50_ of 6.423 μM (Table 6). These findings underscore the importance of incorporating a moderately flexible three-atom linker and precisely positioned hydrophobic and hydrogen-bonding elements for effective BACE-1 inhibition.

**Scheme 62:**
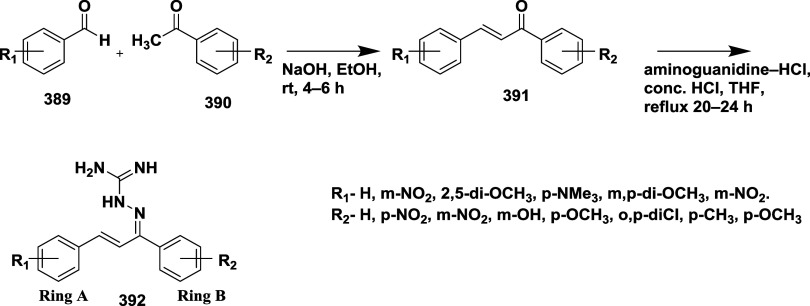
Synthesis of Allylidene Hydrazine Carboximidamide Derivatives **392**

In another report, Jain et al. reported the synthesis of small molecules like sulfonyl-amino-acetamide as a β-secretase inhibitor.^69^ The compounds were obtained in good yield, ranging from 68 to 91%. The synthesis involves the nucleophilic substitution reaction between halides **393** and the amine of glycine, resulting in an intermediate **394** with good yield. HOBT-EDC. HCl-mediated coupling of **394** with substituted aniline produced the desired sulfonyl-amino acetamide **395** with substituted urea as a byproduct (Scheme 63). The SAR analysis highlighted that modifications on ring A had a more pronounced impact on BACE-1 inhibitory activity than those on ring B. Among electron-donating substituents, the para-acetamide group on ring A, when paired with ortho,para-dimethyl substitution on ring B (compound **395**), delivered the highest level of inhibition (61.9%, IC_50_ = 7.90 μM), attributed to strong hydrogen bonding with Asp32, Asp228, and residues spanning the S1 to S3 pockets. Electron-withdrawing substituents such as para-chloro and meta-nitro on ring A enhanced potency by facilitating π-cation interactions and additional hydrogen bonds. In contrast, nitro groups at ortho or para positions and bulky side chains that impaired fit within the active site significantly decreased activity.

**Scheme 63:**
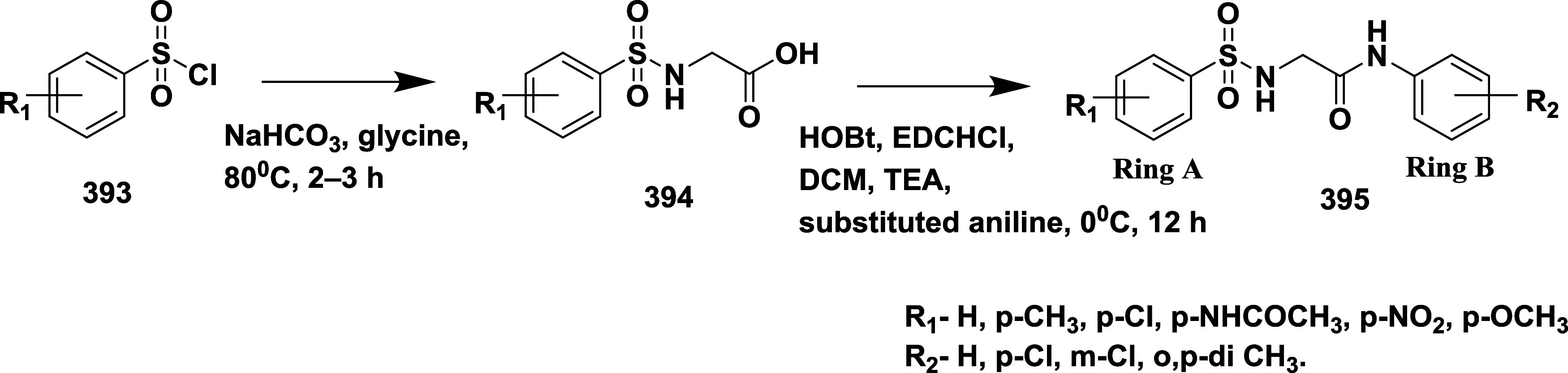
Synthesis of Sulfonyl-Amino-Acetamides **395**

The synthesis of biphenyl-substituted cyclic acyl guanidines as an inhibitor of Alzheimer’s disease was reported by Liu et al.^70^ The synthesis involved the reaction of 1-Bromo-3-iodobenzene **396** with ethynyl-trimethyl silane in the presence of bis (triphenylphosphine) palladium­(II) dichloride, copper iodide, and triethylamine formed an intermediate **397**, which then reacted with a boronic acid in the presence of [1,1′-bis (diphenylphosphine) ferrocene] palladium­(II) dichloride afforded **398**. The reaction of **398** with 1-bromo-3-chlorobenzene yielded **399**, followed by oxidative cleavage of **399** using potassium permanganate in magnesium sulfate and sodium carbonate, which afforded diketones **400**. Cyclization of diketones by reacting with acyl guanidine yielded cyclic acyl guanidines intermediate **401**. Subsequently, the reaction of **401** with 3-bromoprop-1-yne or the corresponding carbamic chloride in acetone at room temperature, yielding target compound **402**. The compounds were obtained with a low yield (12%) (Scheme 64). The SAR investigation revealed that incorporating a third phenyl ring bearing a prop-2-yn-1-yloxy substituent into the cyclic acylguanidine framework markedly improved BACE-1 inhibitory activity by engaging the S3 subpocket and enhancing hydrophobic interactions. Compound **402** emerged as the most potent derivative, exhibiting an IC_50_ of 0.127 μM (Table 1), representing a 60-fold increase in potency compared to the original lead compound 2-imino-3-methyl-5,5-diphenylimidazolidin-4-one. The nature of the substituents on both the phenyl ring (−R) and the side chain (−X) played a significant role in modulating activity; overly bulky or strongly electron-withdrawing groups often reduced efficacy by disrupting optimal binding or failing to occupy the S3 region properly. These results confirm the utility of the prop-2-yn-1-yloxy fragment in refining the activity profile of this inhibitor class.

**Scheme 64:**
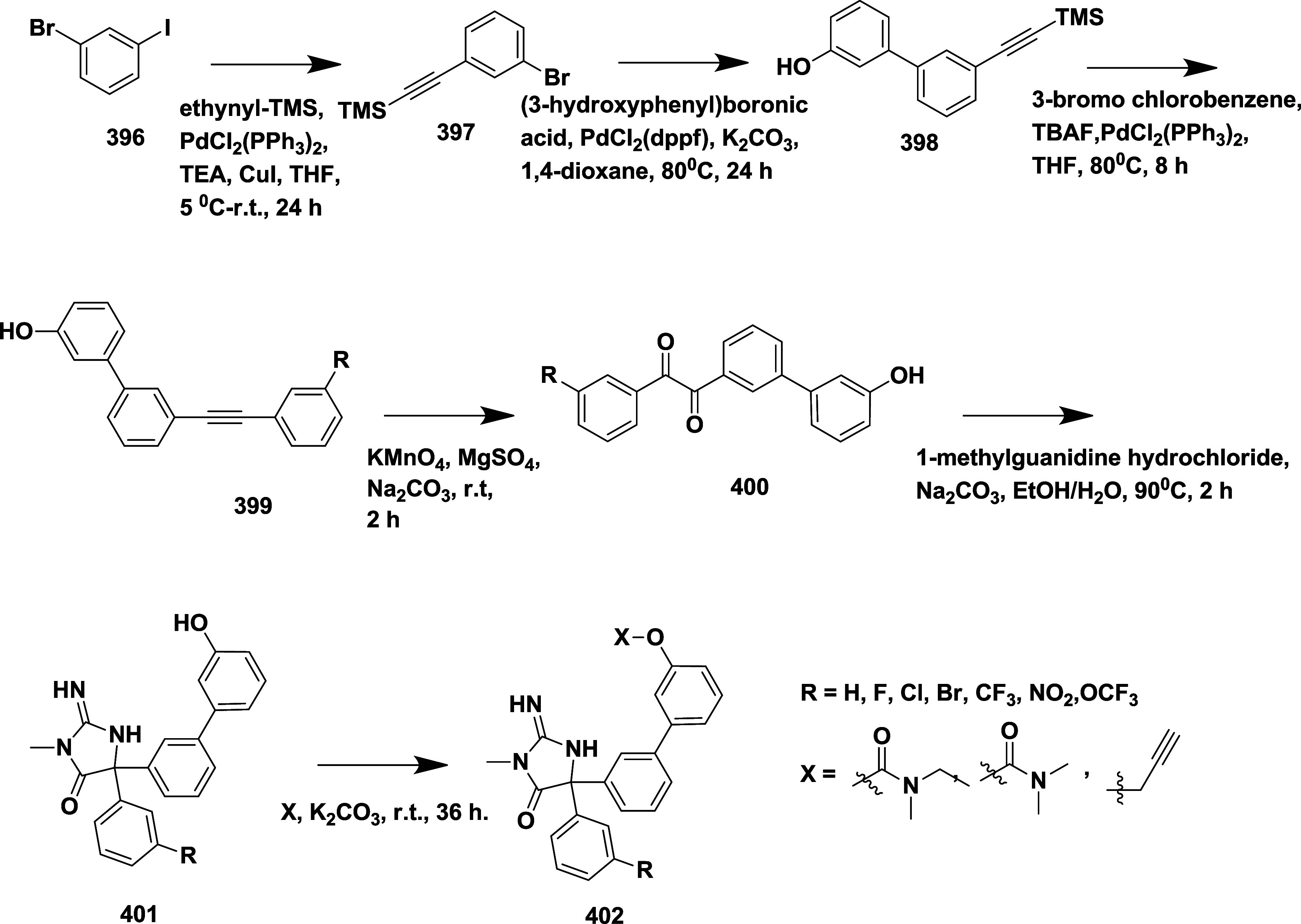
Synthesis of Hydroxy-Substituted Phenylimidazolidin-4-one Derivatives **402**

**Scheme 65:**
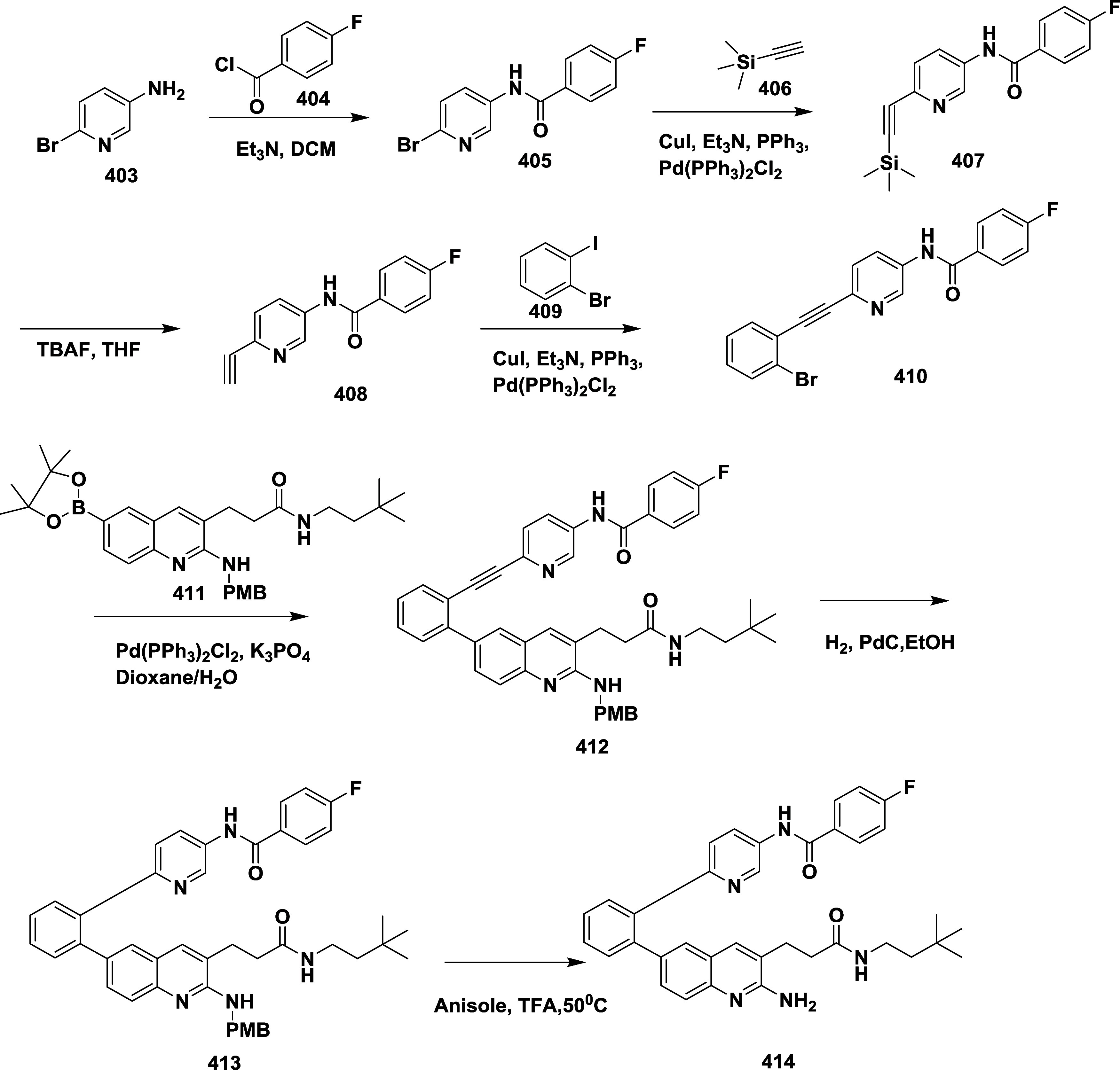
Synthesis of *N*-(6-(2-(2-Amino-3-(3-((3,3dimethylbutyl)­amino)-3-isopropyl)­quinoline-6-yl)­phenyl)­pyridine-3-yl)-4-fluoro Benzamide **414**

Jordan et al. synthesized a novel β-secretase inhibitor for treating AD.^71^ The reaction of 6-bromopyridin-3-amine **403** with 4-fluorobenzoyl chloride **404** in DCM yielded the crude *N*-(6-bromopyridin-3-yl)-4-fluorobenzamide. The obtained crude was then treated with trimethylsilylacetylene under a nitrogen atmosphere in the presence of a copper catalyst to yield 4-Fluoro-*N*-(6-((trimethylsilyl)­ethynyl) pyridine-3-yl) benzamide **407**. Intermediate **407** was then treated with TBAF to yield *N*-(6-ethynylpyridin-3-yl)-4-fluorobenzamide 408, which was then treated with bromo-2-iodobenzene in the presence of palladium catalyst to yield *N*-(6-((2-Bromophenyl) ethynyl) pyridine-3-yl)-4-fluorobenzamide **410**. The Pd­(PPh_3_)_2_Cl_2_ catalyzed reaction of intermediate **410** with *N*-(3,3-dimethylbutyl)-3-(2-((4 methoxybenzyl)­amino)-6-(4,4,5,5-tetramethyl-1,3,2-dioxaboro lan-2-yl)­quinolin-3-yl)­propenamide **411** to yield *N*-(6-((2-(3-(3-((3,3-dimethylbutyl)­amino)-3-oxopropyl)-2-((4-methoxybenzyl)­amino)­quinolin-6-yl)­phenyl)­ethynyl)­pyridin-3-yl)-4-fluorobenzamide **412**. The compound **412** was reduced with H_2_ in the presence of a Pd catalyst to yield **413**, which was then treated with TFA to yield 69% of the final product *N*-(6-(2-(2-amino-3-(3-((3,3dimethylbutyl)­amino)-3-isopropyl)­quinoline-6-yl)­phenyl)­pyridine-3-yl)-4-fluoro benzamide **414** (Scheme 65). This study employed a fragment-linking strategy guided by ^19^F NMR to develop BACE-1 inhibitors with enhanced potency and remarkable selectivity over cathepsin D. Optimization of aromatic and polar substituents, along with careful linker design, improved binding to the S3 region while minimizing off-target effects. Crystallographic analysis confirmed the predicted binding interactions, supporting the structure-based SAR approach. Compound **414** was more potent, with an IC_50_ value of 0.8 nM against the BACE-1 enzyme (Table 6). This compound was selective in inhibiting BACE-1 by penetrating deep into the S3 subpocket. However, it suffered a significant drawback due to complex synthetic procedures and failed to show optimal drug-like properties.

Azimi et al. synthesized a novel class of imidazopyridines that inhibit BACE1 by containing isoindoline-1,3-dione derivatives.^72^ The synthesis involves the reaction of phthalimide **422** with 1,3-dibromopropane **423** in the presence of potassium carbonate in acetone at reflux, which gave 2-(3-bromopropyl) isoindoline-1,3-dione **424**. The phthalimide-substituted aldehyde **426** was then produced by compound **424** reacting with 4-hydroxy aldehyde derivatives **425** in the presence of potassium carbonate in *N*,*N*-dimethylformamide. The target compounds **429** were made by the reaction of aldehyde **426**, 2-aminopyridine **427**, and isocyanides **428** with ammonium chloride (Scheme 66). The compounds were obtained in good yield, ranging from 67 to 94%. The structure–activity relationship (SAR) study of imidazopyridine–phthalimide hybrids demonstrated that introducing an aminocyclohexyl group at the imidazopyridine core considerably increased BACE-1 inhibitory potency compared to its *tert*-butyl-substituted counterparts. Within this series, the compound featuring a 7-methyl group on the imidazopyridine ring (compound **422**) exhibited the highest activity, achieving an IC_50_ of 2.84 μM (Table 6), attributed to favorable interactions within the S2 and flap regions of BACE-1. Analysis of methyl substitution patterns revealed a potency order of 7 > 5 > 6 ≈ 8, indicating that both steric and electronic effects play significant roles in binding affinity. Furthermore, the presence of methoxy groups in the phenoxypropyl linker affected inhibitory activity selectively, depending on the core modifications, likely influencing the spatial orientation and accessibility to key catalytic residues.

**Scheme 66:**
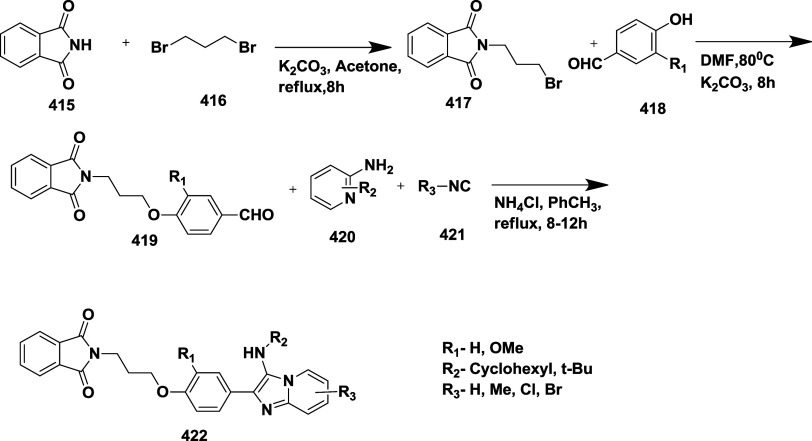
Synthesis of Imidazopyridines Bearing Phthalimide Moiety **422**

Haghighijoo et al. reported the synthesis of quinazolinone-based hydrazones as a BACE-1 inhibitor.^73^ The synthesis starts with anthranilic acid **423** under basic conditions, treated with methyl thiocyanate to give a high yield of 2-mercapto-quinazolinone **424**. Subsequently, hydrazinolysis of **424** using hydrazine hydrate in butanol to afford 2-hydrazinyl-3-methylquinazoline derivative **425**. The reaction of **425** with different aldehydes or isatin in the presence of acid produced the desired Schiff base derivatives **426–428** (Scheme 67). The compounds obtained good yields ranging from 46 to 87%. SAR investigations indicated that quinazolinone-derived hydrazones with a 2,3-dichlorophenyl group (compound **427**, X = 2,3-Cl_2_) displayed the most substantial BACE-1 inhibitory effect, achieving an IC_50_ of 3.7 μM (Table 6) and surpassing analogues with mono- or differently positioned substitutions. The position of chlorine atoms was crucial, as analogs substituted at the 2,4- or 4-positions showed little to no activity, highlighting the importance of chlorine positioning for proper alignment in the enzyme active site. Additionally, para-hydroxyl-substituted compounds demonstrated improved inhibition, likely due to their capacity to form hydrogen bonds. Docking studies further validated these observations by revealing that compound **427** established key interactions with Asp32, Asp228, and Thr231 in the BACE-1 active site. Collectively, the SAR data highlight the essential roles of electronic characteristics and precise substituent placement in achieving effective and selective inhibition.

**Scheme 67:**
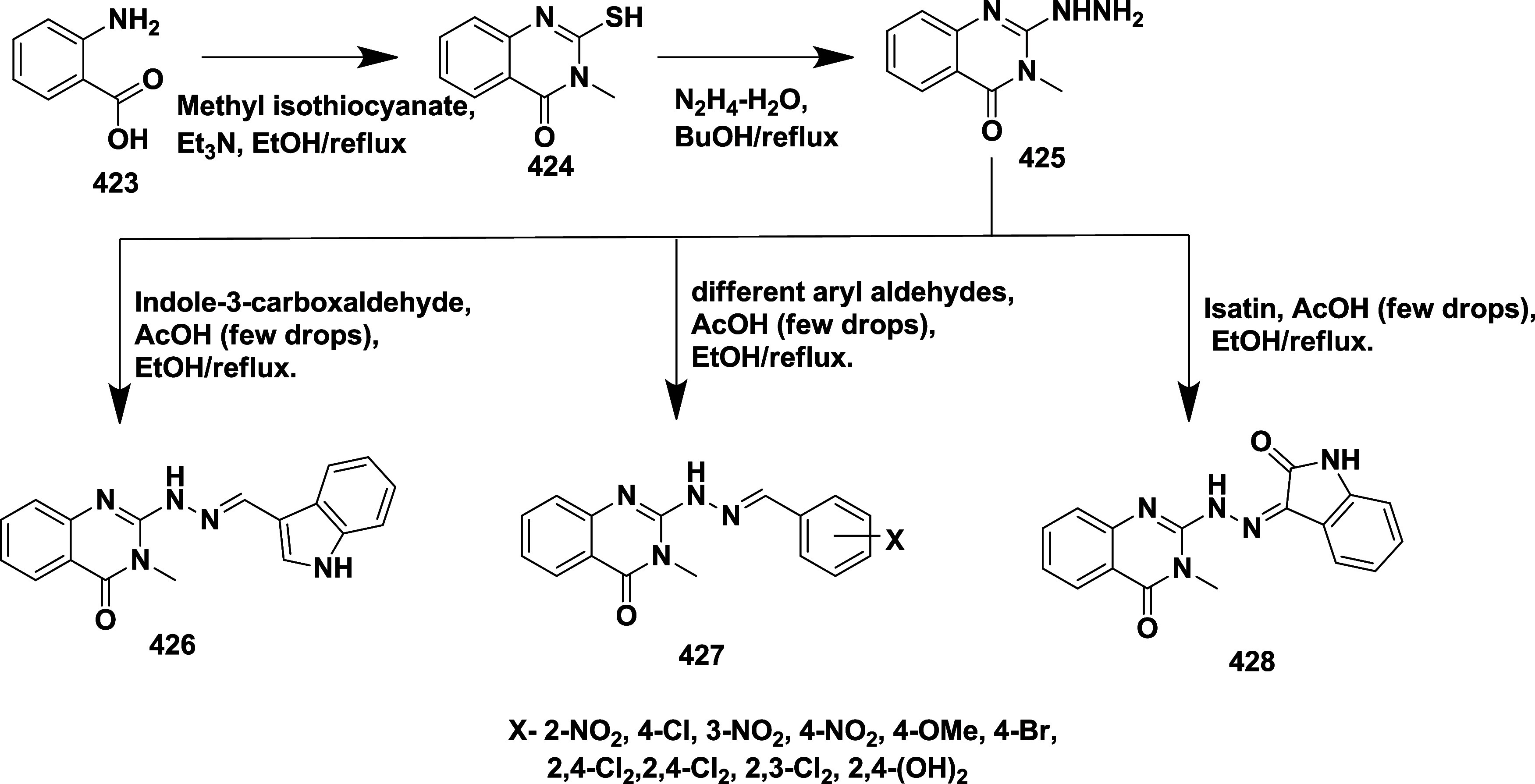
Synthesis of 3-Methylquinazolin-4­(3*H*)-one-hydrazone Derivatives **426**–**428**

Ghosh et al. reported the synthesis of substituted 2-oxopiperazines as a BACE-1 inhibitor.^74^ The synthesis involves the aldol reaction between substituted 4 4-Boc-2-oxopiperazines **430** and (*S*)-*N*,*N*-dibenzyl phenylalanine **429** in the presence of Lithium hexamethyldisilazide, which afforded aldol intermediate **431**. By catalytic hydrogenation over Pearlman’s catalyst, **431** yields the corresponding amines **432**. The coupling of Amines **432** with substituted isophthalic acid derivative **433** in dichloromethane in the presence of 1-Ethyl-3-(3-(dimethylamino)­propyl)­carbodiimide hydrochloride, 1-hydroxybenzotriazole (HOBT), and diisopropylethylamine (DIPEA) afforded inhibitors **434** in good yields (Scheme 68). When R = Ph, compound **434** showed potent activity with a *K_i_* value of 2 nM, but this compound was found to destabilize the hydrogen bonding with the –NH group of the Thr flap. Hence, inhibitor **437** was synthesized (Scheme 69) to overcome this drawback. The preparation of intermediate **435** from the aldol reaction of oxopiperazine and **429**. The carbonyl group on **428** was reduced by treating it with borane-dimethyl sulfide complex to give compound **436**. This compound was then coupled with acid **433** to yield **437**, which, upon further treatment with TBAF in THF, removed the TBS group to afford amino alcohol. Exposure of **437** with CDI in dioxane gave a corresponding oxazolidinone compound. This resulted in oxazolidinone reacting with TFA to yield potent compound **438** with over 30% yield. SAR findings showed that introducing an *N*-benzyl group to the 2-oxopiperazine scaffold markedly improved BACE-1 inhibitory activity compared to other *N*-alkyl variants. Substituting with methoxy groups or incorporating indole-based P2 ligands led to decreased potency, emphasizing the need for optimal steric and electronic fit within the S2 binding pocket. Exploration of 6-substituted and bicyclic 2-oxopiperazines revealed that both the type and stereochemistry of the 6-substituent played a vital role, with the (*R*)-benzyl and *N*-methyl substitutions producing the most active analogue (compound **434**, *K_i_* = 12 nM). Another potent derivative, compound **438**, containing an oxazolidinone moiety, achieved a *K_i_* of 23 nM (Table 6) and showed vigorous cellular activity. Structural data from X-ray crystallography confirmed the presence of essential hydrogen bonds and hydrophobic contacts, supporting the SAR observations.

**Scheme 68:**
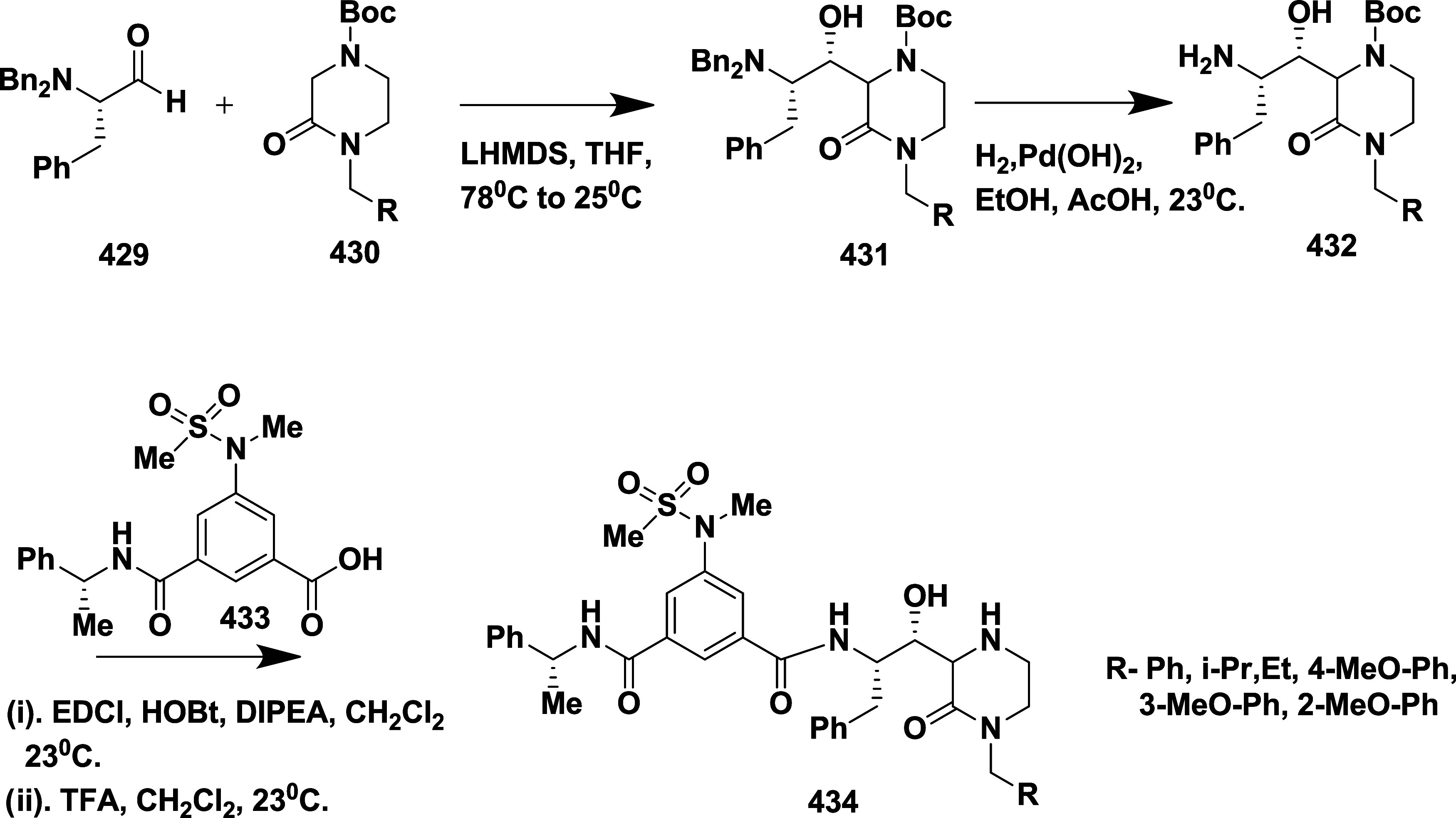
Synthesis of Hydroxy-phenylpropyl-methyl-oxopiperazinyl-methylsulfonamido-phenylethyl-isophthalamide Derivatives **434**

**Scheme 69:**
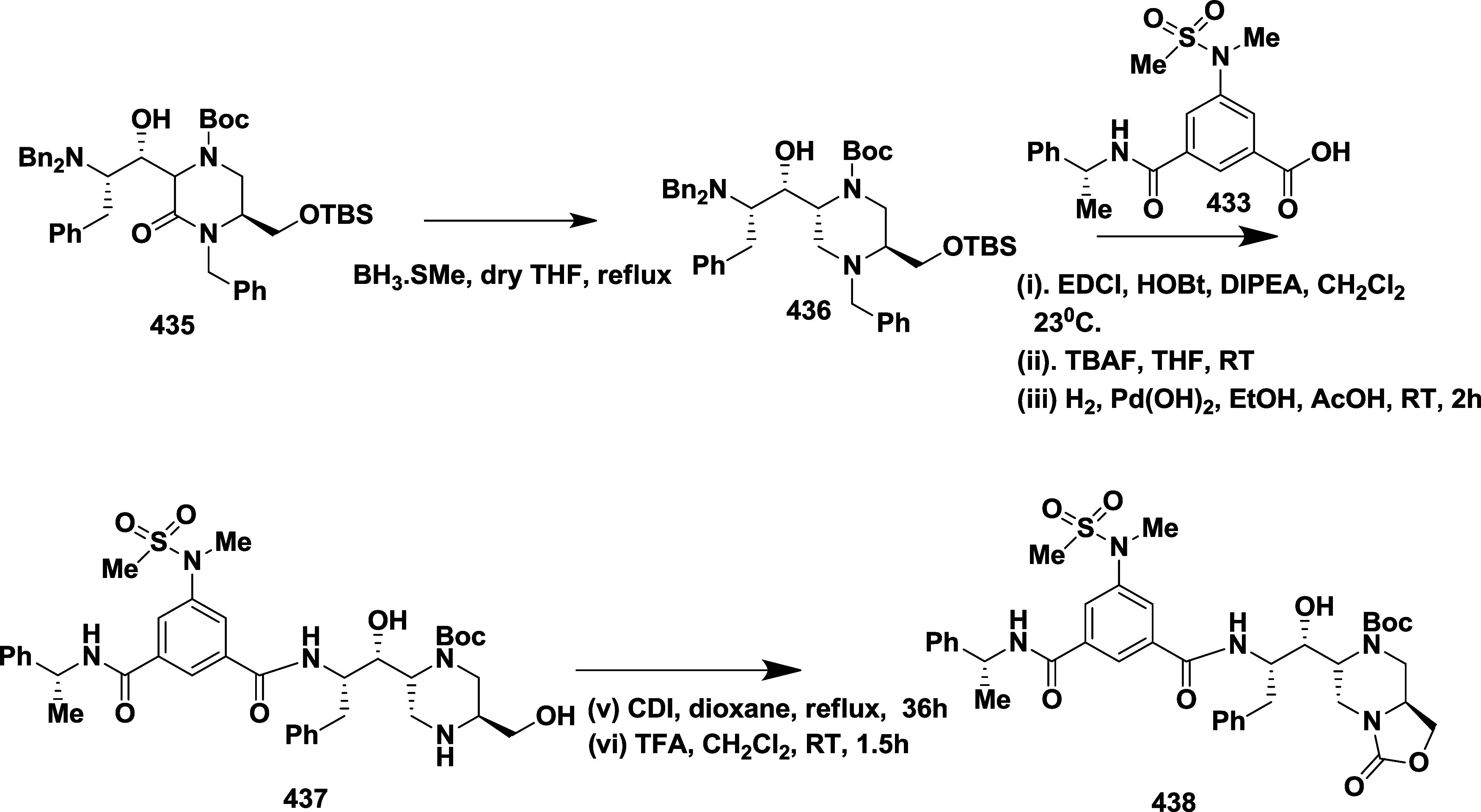
Synthesis of Oxazolidinone Derivatives **438**

Innocenti et al. synthesized new bicyclic acetals as β-secretase inhibitors.^75^ The synthesis involved the reaction of bicyclic lactams **439** with Lawesson’s reagent in dry toluene, obtaining an intermediate **440**. The aminolysis of **440** with secondary amines at 45 °C produced a target compound **441** (Scheme 70). The compounds were obtained in good yields, ranging from 61 to 71%. SAR analysis revealed that a thiocarbonyl (C = S) group within the bicyclic acetal framework was essential for BACE-1 inhibitory activity, as analogues without this feature were largely inactive. Among the thiolactam derivatives, compound **441**, incorporating a morpholine ring and a benzyl side chain, showed the greatest potency with an IC_50_ of 2.6 μM (Table 6). The type of heterocyclic group, such as morpholine or piperazine, significantly influenced activity, indicating its role in interacting with the catalytic dyad and the enzyme’s flap region. Docking studies supported these results, showing that compound **441** established hydrogen bonds with Asp93 and Asp289 and engaged in hydrophobic contacts with residues F169, W176, and Q134, confirming its optimal positioning in the active site. Overall, the data highlight the critical role of the thiocarbonyl group, substituent placement, and scaffold geometry in enhancing BACE-1 inhibition.

**Scheme 70:**
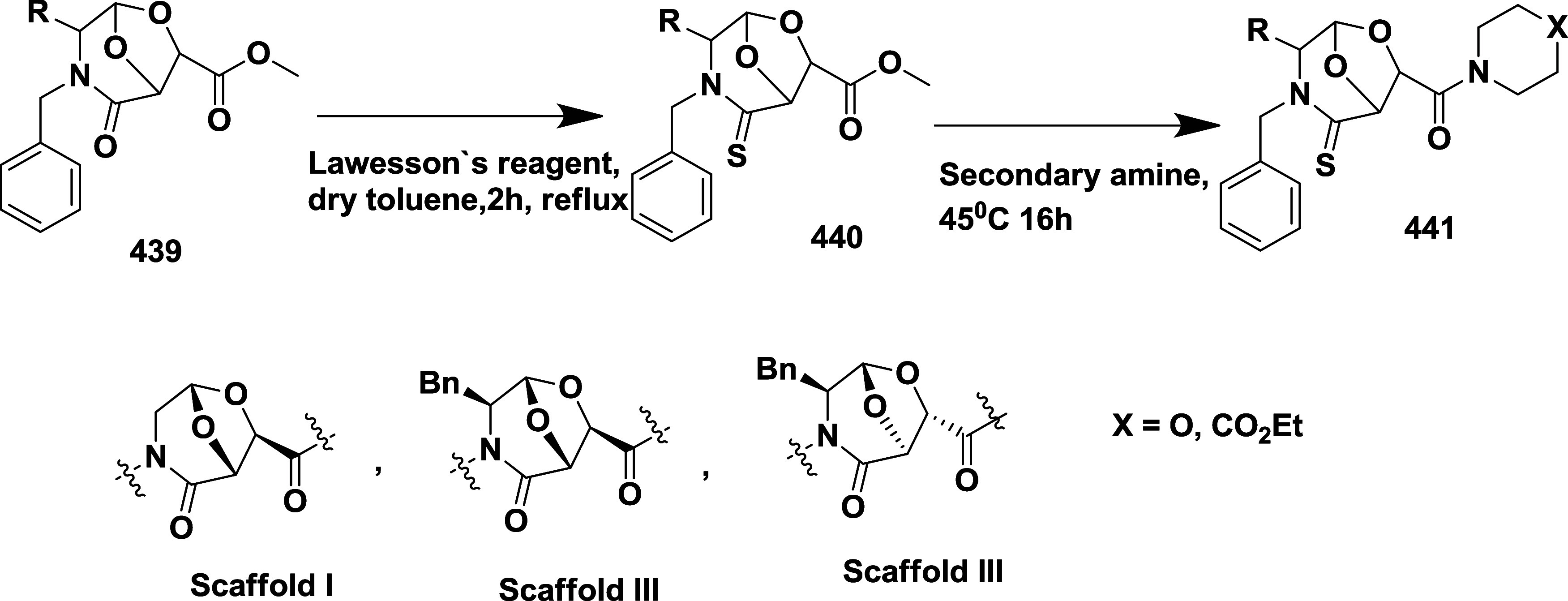
Synthesis of Bicyclic Acetals **441**

Tarazi et al. reported the synthesis of isophthalic acid derivatives armed with imidazole and indolyl groups as potent β-secretase inhibitors.^76^ The synthesis involved the Hell–Volhard–Zelinsky reaction (α-bromination) of methyl-2-benzoate with bromine (Br_2_), yielding bromomethoxy intermediate **443**. The nucleophilic substitution of **443** with 1*H*-indolecarboxaldehyde derivative in the presence of DIPEA, followed by reductive amination and ester hydrolysis, yielded an intermediate **444**. The TBTU-mediated amide coupling of **444** offered target compound **445** (Scheme 71). In Scheme 72, instead of methyl-2-benzoate, the 1-(3-nitrophenol) ethanone is used, and in the third step, catalytic hydrogenation of **449** produces **446**.

**Scheme 71:**
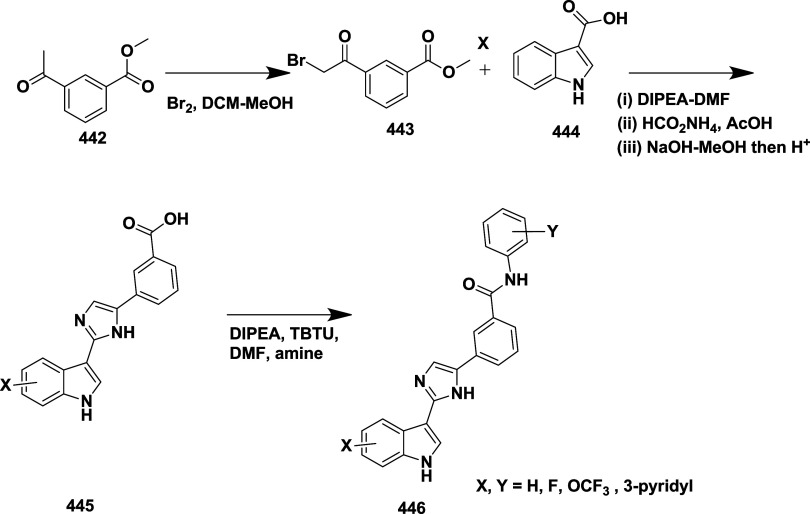
Synthesis of 3-(2-(1*H*-indol-3-yl)-1*H*-Imidazol-5-yl)-*N*-phenyl Benzamides **446**

**Scheme 72:**
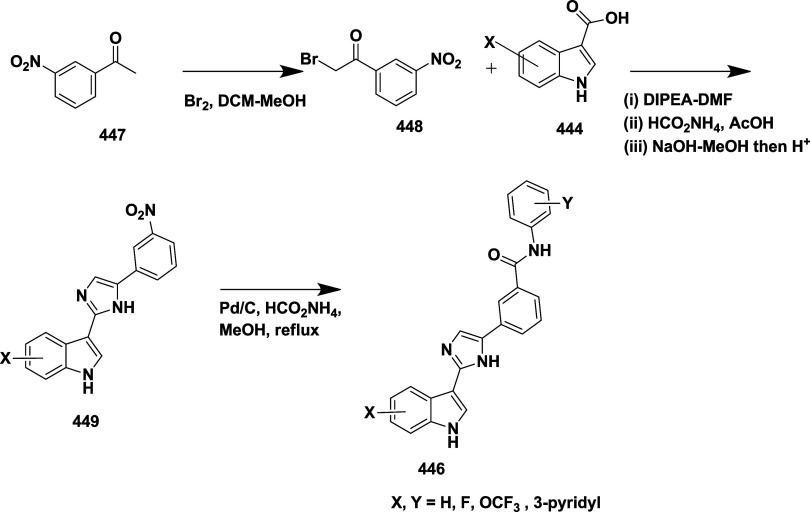
Synthesis of 3-(2-(1*H*-Indol-3-yl)-1*H*-imidazol-5-yl)-*N*-phenyl Benzamides **446**

In Scheme 73,^76^ introducing the phenyl group at the 4-position of the imidazole moiety by reacting with phenylenediamine yielded the final compound **450**. The nucleophilic substitution of **445** with 1*H*-indolecarboxaldehyde derivative in the presence of DIPEA, followed by reductive amination and ester hydrolysis, yielded an intermediate **451**. The yield of synthesized compounds ranges from 45 to 75%. The compound **451**(45% yield) containing X= F group showed increased BACE-1 inhibition compared to **446**. SAR analysis revealed that incorporating indole and imidazole units into isophthalic acid-derived frameworks enhances BACE-1 inhibition. Substituting peptide-like structures with conformationally rigid heterocycles, especially aryl-substituted imidazoles, improved binding interactions within the S1–S3 and S2′ regions and promoted better permeability across the blood–brain barrier. Among the synthesized compounds, **451** (X = F) demonstrated potent enzyme inhibition with an IC_50_ of 75 nM, while its OCF_3_ analogue exhibited superior cellular activity (EC_50_ = 0.29 μM). Docking studies confirmed consistent hydrogen bonding between the indole ring and Asp32, with halogen substitutions playing a key role in optimizing binding orientation and depth. Strategic scaffold rigidification and fine-tuning of physicochemical properties such as PSA and logP contributed to the development of effective and brain-accessible inhibitors with favorable drug-like characteristics.

**Scheme 73:**
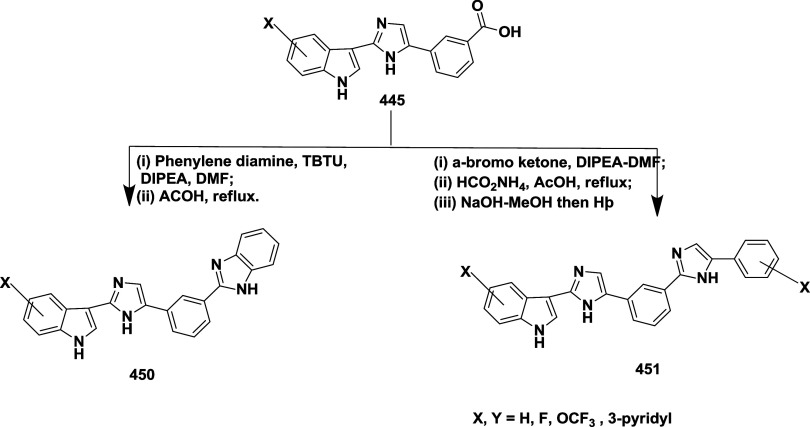
Synthesis of 3-(5-(3-(5-Methyl-1*H*-imidazol-2-yl) Phenyl)-1*H*-imidazol-2-yl)-1*H*-indole Derivatives **450** and **451**

A series of new 2-substituted-thio-*N*-(4-substituted-thiazol/1*H*-imidazol-2-yl) acetamide derivatives were developed by Yan et al. as BACE-1 inhibitors.^77^ The synthesis involved the target compounds **460**, which were prepared in 3–6 steps, starting with thiourea **452**, substituted aldehydes **453**, and ethyl cyanoacetate **454**, which produced compounds **455** under basic conditions. An alternative pathway began with 1-(4-amino-3,5-dichlorophenyl) ethanone **456**, subsequently forming an intermediate bromoethanone through a reaction with bromine. The reaction between the substituted phenyl bromoethanone intermediate and thiourea to form 4-substituted-2-aminothiazole **457**. Subsequently, it reacts with chloroacetyl chloride in the presence of base to offer 2-chloro-*N*-(thiazol-2-yl) acetamide **459**. The nucleophilically substituting compounds 458, **460**, **461**, and **462** were produced, and the desired target compounds **463**, **464**, **and 465**. (Scheme 74). Substituted bromo ethanone **457** was converted into *N*-(1*H*-imidazol-2-yl) acetamide **466** by heating with *N*-acetyl guanidine. Compound **466** was hydrolyzed in an acidic environment to yield the intermediate 2-chloro-*N*-(1*H*-imidazole–2-yl) acetamide **467**, which was then reacted with chloroacetyl chloride. After that, compound **467** was treated with **468** to form the final targeted acetamide derivative **468** (Scheme 75). The yield of synthesized compounds ranges from 35 to 91%. The SAR evaluation indicated that meta-substituents on the phenyl rings at both R1 and R2 positions significantly improved BACE-1 inhibition, with compound **468** (R_1_ = 3,5-dichloro-4-aminophenyl; R_2_ = meta-methoxyphenyl) showing the highest activity (IC_50_ = 4.6 μM, Table 6), along with intense BBB penetration and low toxicity. In contrast, para- and ortho-substituted analogues were less effective, likely due to unfavorable steric or electronic effects in the binding pocket. Substituting the thiazole sulfur with a nitrogen atom strengthened binding interactions, forming a stable bidentate hydrogen bond with Asp32 and Asp228. Furthermore, introducing bulky meta-substituents such as bromine or additional aromatic rings improved interaction with the S3 subsite, as supported by molecular docking studies.

**Scheme 74:**
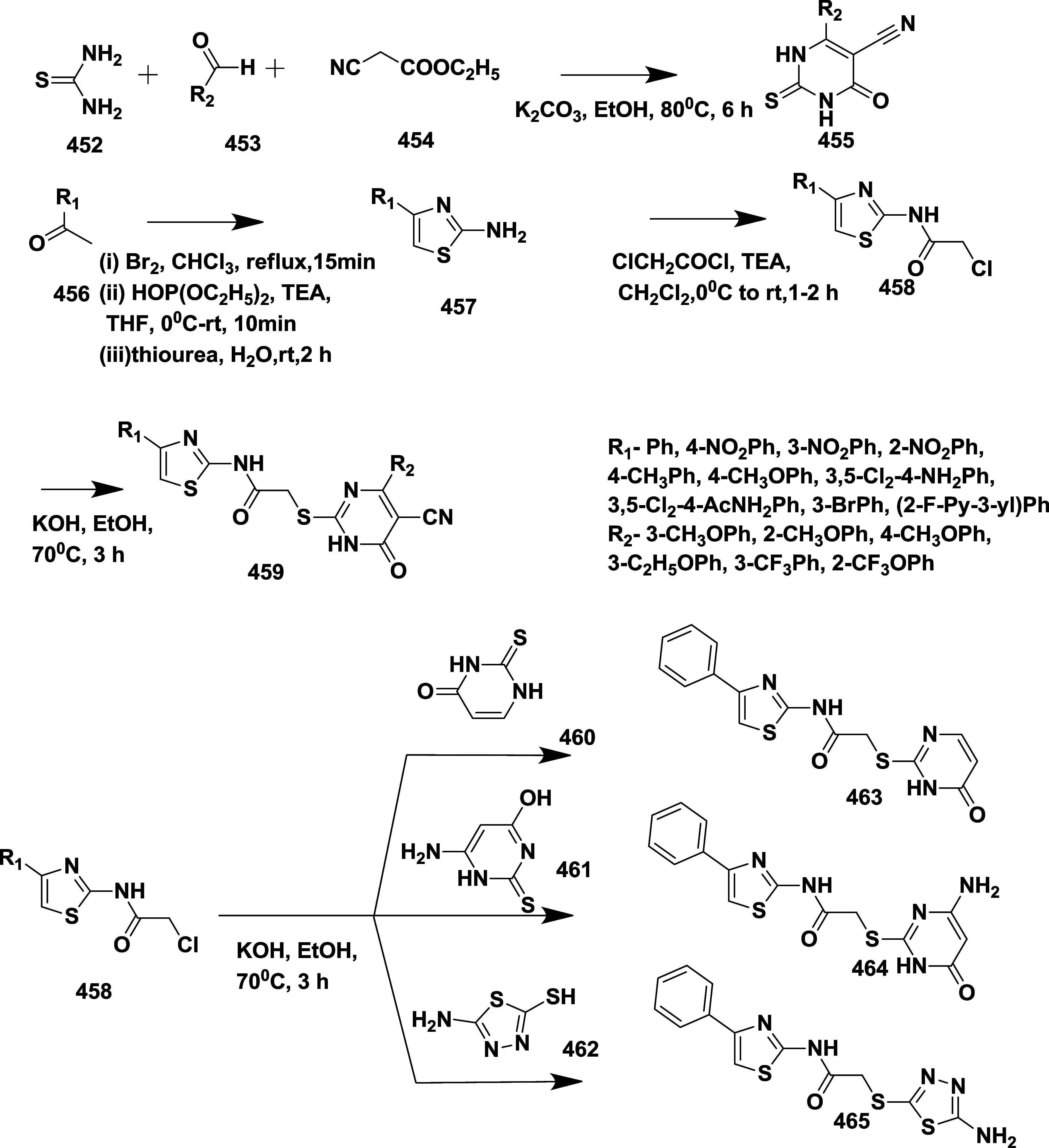
Synthesis of 2-Thio-*N*-(thiazol-2-yl) Acetamide Derivatives **463**–**465**

**Scheme 75:**
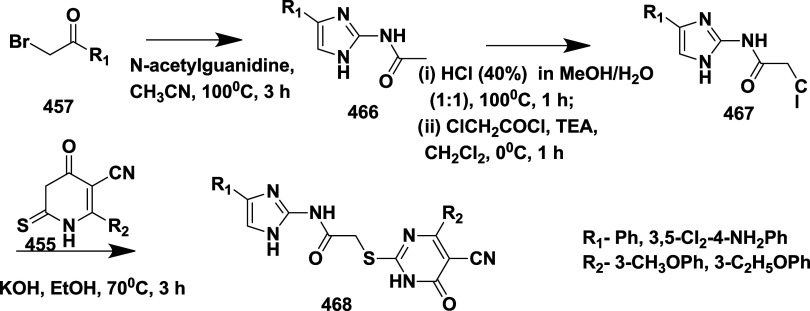
Synthesis of 2-Thio-*N*-(1*H*-imidazol-2-yl) Acetamide Derivatives **468**

Low et al. reported the enantiomer synthesis of fused cyclopropyl-2,4-oxazines as BACE-1 inhibitors.^78^ The synthesis starting from imidoyl chloride forms a corresponding nitrile oxide, followed by 1,3-dipolar cycloadditions with allyl chloride, yielding racemic 5-chloromethyl isoxazolines. The cyclization of **470** in the presence of KOtBu yielded **471**. The **471** was activated and treated with 4-bromo-1-fluoro-2-lithiobenzene, yielding 473, followed by DCC-catalyzed cyclization, which obtained *N*-benzoyl fused isoxazoline **474**. The copper-catalyzed coupling of **474** with sodium azide and the intermediate obtained by undergoing reduction using sodium borohydride afforded **474**. The amide coupling of **475** with 5-methoxypyrazine-2-carboxylic acid, followed by acid cleavage and chiral separation, yielded the desired compound **476** with its enantiomer **477** (Scheme 76). The yield of synthesized compounds ranges from 50 to 60%. The SAR study highlighted the influence of stereochemistry and electronic effects in fused cyclopropyl-3-amino-2,4-oxazine derivatives targeting BACE-1. The *(S*)-trans isomer (compound **476**) exhibited the most potent inhibitory activity (IC_50_ = 81 nM, Table 6), surpassing both the cis-isomer and the original oxazine scaffold, attributed to its optimal fit within the active site and a reduced p*K*_a_. Computational modeling supported this finding by showing that **476** adopts a favorable low-energy conformation, whereas the cis-isomer suffers from higher energy penalties, accounting for its lower potency. The fused cyclopropyl ring also contributed to decreased amidine basicity and enhanced CNS-relevant characteristics such as better brain permeability and reduced efflux via P-glycoprotein.

**Scheme 76:**
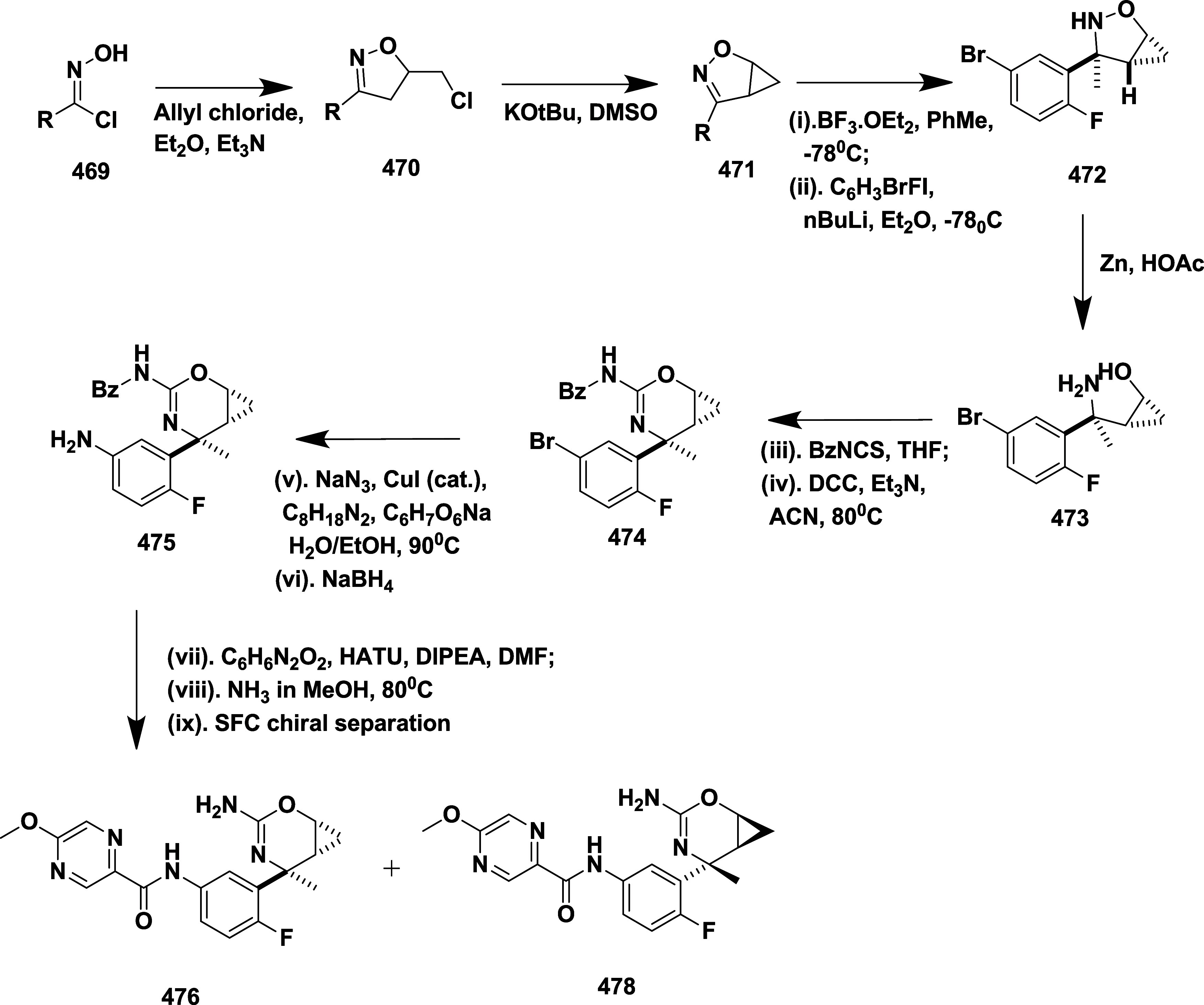
Synthesis of Fused Cyclopropyl-3-amino-2,4-oxazines **476**

**Scheme 77:**
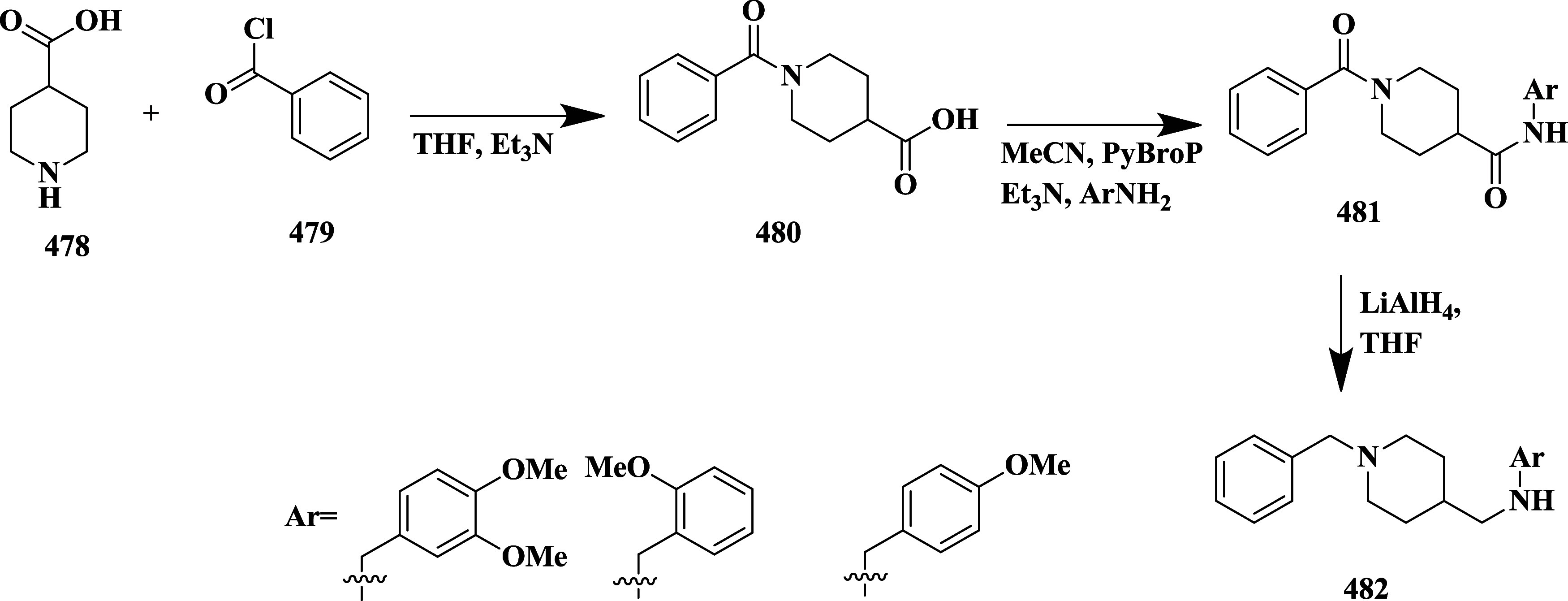
Synthesis of Donepezil Derivatives **482**

Gabr et al. reported the synthesis of novel donepezil analogues as inhibitors of AD.^79^ The synthesis involved the microwave imidation of isonipectotic acid **478** with benzoyl chloride **479** in the presence of triethylamine as an intermediate 1-benzoylpiperidine-4-carboxylic acid **480**. The coupling of **480** with benzylamine under microwave irradiation, followed by lithium aluminum hydride reduction, yielded donepezil analogs **482** with a higher yield (Scheme 77). The yield of synthesized compounds ranges from 76 to 94%. The SAR investigation involved structural modification of the donepezil core by incorporating amide backbone linkers. This facilitated additional hydrogen bonding with BACE-1’s catalytic residues Asp32 and Asp228, enhancing dual AChE and BACE-1 inhibitory activity. Among the series, compound **481** (Table 6) showed the most effective dual inhibition, with IC_50_ values of 4.11 nM for AChE and 18.3 nM for BACE-1, surpassing the activity of donepezil. Analogues lacking the amide linkage demonstrated significantly weaker BACE-1 inhibition, underscoring the importance of this structural feature. Docking studies confirmed that compound **481** interacted with both the CAS and PAS regions of AChE and established multiple hydrogen bonds and π–π stacking within the BACE-1 active site. This compound exhibited excellent BBB penetration, low cytotoxicity, and metal-binding properties, positioning it as a strong multitarget candidate for Alzheimer’s treatment.

Bhaskar et al. reported the synthesis of tetrahydrobenzo [b]­pyran derivative through a green approach using a 1-Butyl-3-methylimidazolium chloride ([bmim]­Cl) as a reusable ionic liquid.^80^ In the presence of ionic liquid [bmim]­Cl-, one pot reaction of various aldehydes, cyclohexadiene, and malononitrile, stirred at room temperature, obtained the new tetrahydrobenzopyran **486** with a yield of 85–95% (Scheme 78). The yield of synthesized compounds ranges from 74 to 96%. The SAR analysis revealed that variations in electron-donating and heteroaryl substituents on the aryl aldehyde moiety substantially impacted the BACE-1 inhibitory potency of tetrahydrobenzo­[*b*]­pyran derivatives. Compound **486**, featuring a 2-furyl substituent, showed the highest potency with an IC_50_ of 0.91 μM (Table 6). Functional groups such as hydroxy, methoxy, and nitro on the aromatic ring also influenced activity, likely by affecting hydrogen bonding potential and overall lipophilicity within the enzyme’s binding site. Molecular docking confirmed interactions with key residues Asp228 and Thr232, validating the predicted binding orientation. These findings underscore the significance of substituent positioning and heterocyclic incorporation in enhancing BACE-1 inhibition.

**Scheme 78:**
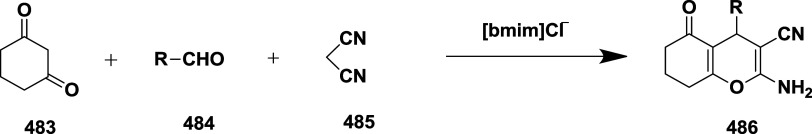
Synthesis of Tetrahydrobenzo [B] Pyran Derivatives **486**

Jagtap et al. reported the synthesis of novel 4-substituted 2-amino-3,4-dihydroquinazolines as β-secretase inhibitors.^81^ The synthesis starts by converting the alcohol **487** to benzyl bromide using phosphorus tribromide. These bromo derivatives and mesityl esters were treated with amines using potassium carbonate as a base to yield the corresponding compounds **449**s. Nitro compounds **489** were reduced with iron in acetic acid to offer the corresponding anilines. The 2-aminodihydroquinazoline derivatives **490** were obtained by reacting these anilines with cyanogen bromide. (Scheme 79). The yield of synthesized compounds ranges from 16 to 90%. Compound **490** (if R_1_ = Me, R_2_ = 1,4-dimethyl-1*H*-pyrazole) showed β-secretase inhibition with an IC_50_ value of 0.38 μM (Table 6). The SAR of 4-substituted 2-amino-3,4-dihydroquinazolines demonstrated that alterations at the C4 position play a crucial role in modulating BACE-1 inhibition. Small, nonpolar substituents such as methyl and cyclohexylmethyl at this site led to enhanced activity by favorably occupying the S1′ subpocket. Incorporation of an *N*-((1-methyl-1*H*-pyrazol-4-yl)­methyl) moiety into the amide side chain improved interactions with the S2 region, thereby boosting inhibitory potential. In contrast, larger or rigid groups like benzyl were less effective, likely due to steric hindrance or poor accommodation within the binding site. Overall, the most promising compounds displayed inhibitory profiles comparable to the known reference (*S*)-2 in both enzymatic and cellular assays, supporting their viability as nonpeptidic BACE-1 inhibitors.

**Scheme 79:**
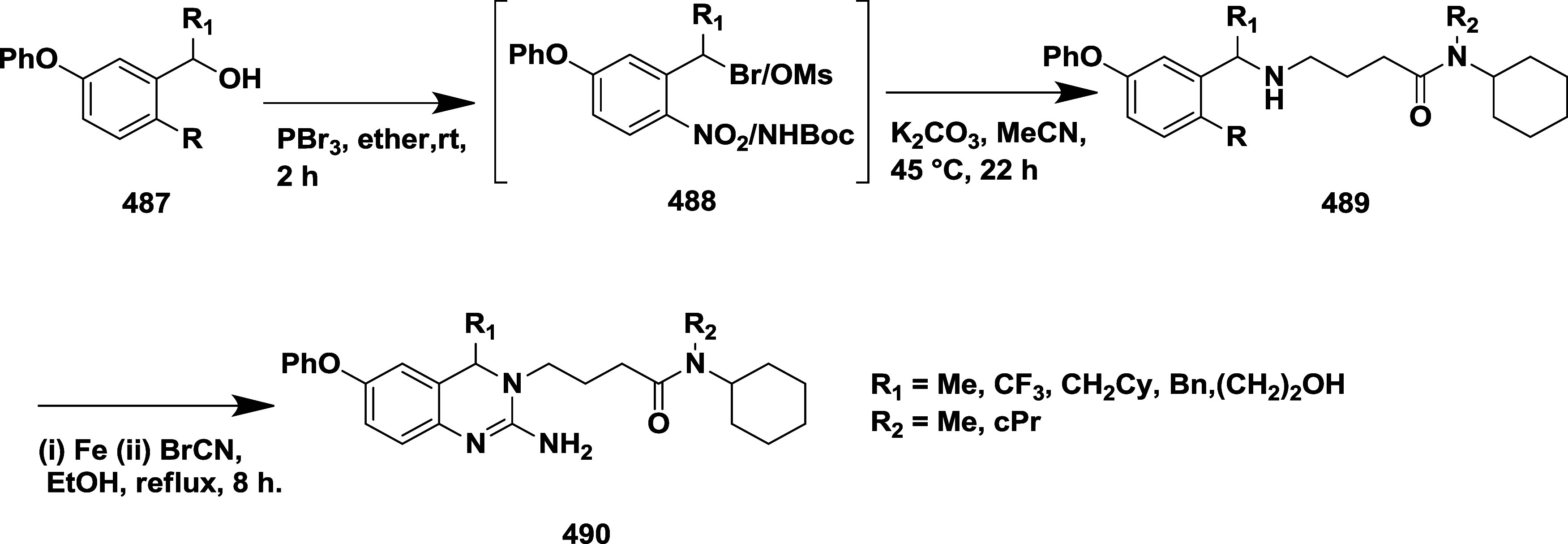
Synthesis of 2-Aminodihydroquinazoline Derivatives **490**

Fang et al. reported the synthesis of tetrahydroisoquinoline-benzimidazole as a multitarget inhibitor of AD.^82^ The synthesis involved the Aube-Schmidt rearrangement reaction of compound **491** in the presence of sodium azide, followed by lithium aluminum hydride catalyzed reduction, which produced an intermediate **493**. Copper­(I) iodide catalyzed the Ullmann coupling reaction of amines **493** with ethyl 6-bromopicolinate to afford **495**. The intermediates **496**, obtained by hydrolysis of the ester 495, were then coupled with the diaminobenzene derivatives, and their rings were formed by an acid catalyst to offer the benzimidazole derivatives **498** (Scheme 80). Similarly, tetrahydroisoquinoline derivatives **504** were synthesized as mentioned in Scheme 80 (Scheme 81). The yield of synthesized compounds ranges from 20 to 50%. The structure–activity relationship study of tetrahydroisoquinoline-benzimidazole hybrids highlighted the critical influence of substitutions on the benzimidazole ring in modulating BACE-1 inhibition and antineuroinflammatory effects. Derivatives bearing electron-withdrawing groups such as 6-OCF_3_, 6-Cl, and 6-CF_3_ exhibited notably stronger BACE-1 inhibition, with IC_50_ values between 1.1 and 3.6 μM, whereas analogs containing electron-donating groups like 6-OH and 6-OCH_3_ were comparatively less potent. In assays for nitric oxide suppression in LPS-activated BV2 microglial cells, many compounds demonstrated favorable anti-inflammatory activity, although bulky substituents such as *tert*-butyl were found to diminish this effect. Among the series, compound **498** (Table 6) stood out for its well-balanced pharmacological profile, showing moderate BACE-1 inhibition, strong neuroprotective action through GSH elevation and ROS reduction, and satisfactory blood-brain barrier permeability. Specifically, compound **498** (R_1_ = H, R_2_ = OCF_3_) achieved 98.7% inhibition at 20 μM, emphasizing the importance of strategic electron-withdrawing substitutions on the benzimidazole scaffold for developing multifunctional agents targeting Alzheimer’s pathology.

**Scheme 80:**
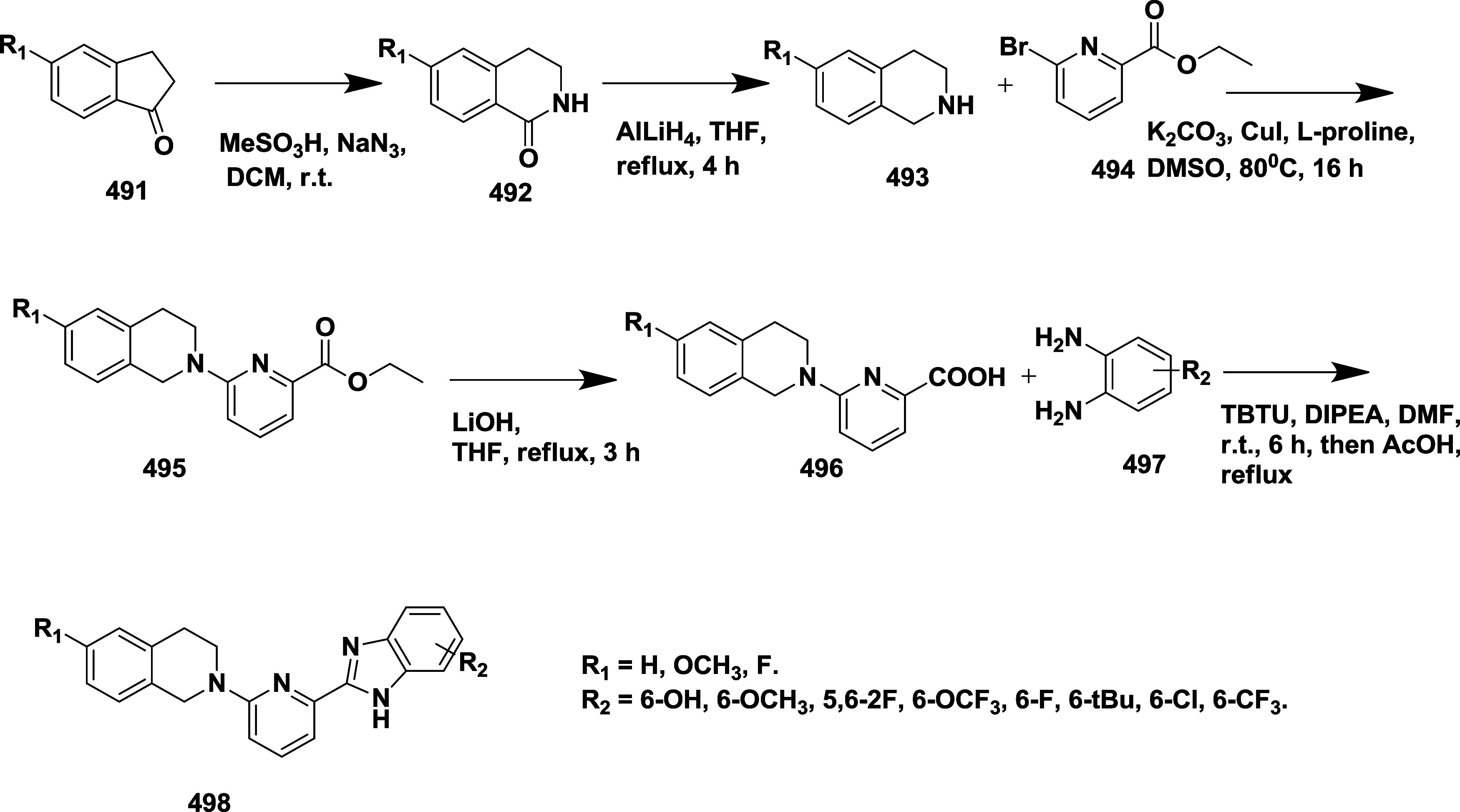
Synthesis of Tetrahydro Naphthyl-phenyl-benzimidazole Derivatives **505**

**Scheme 81:**
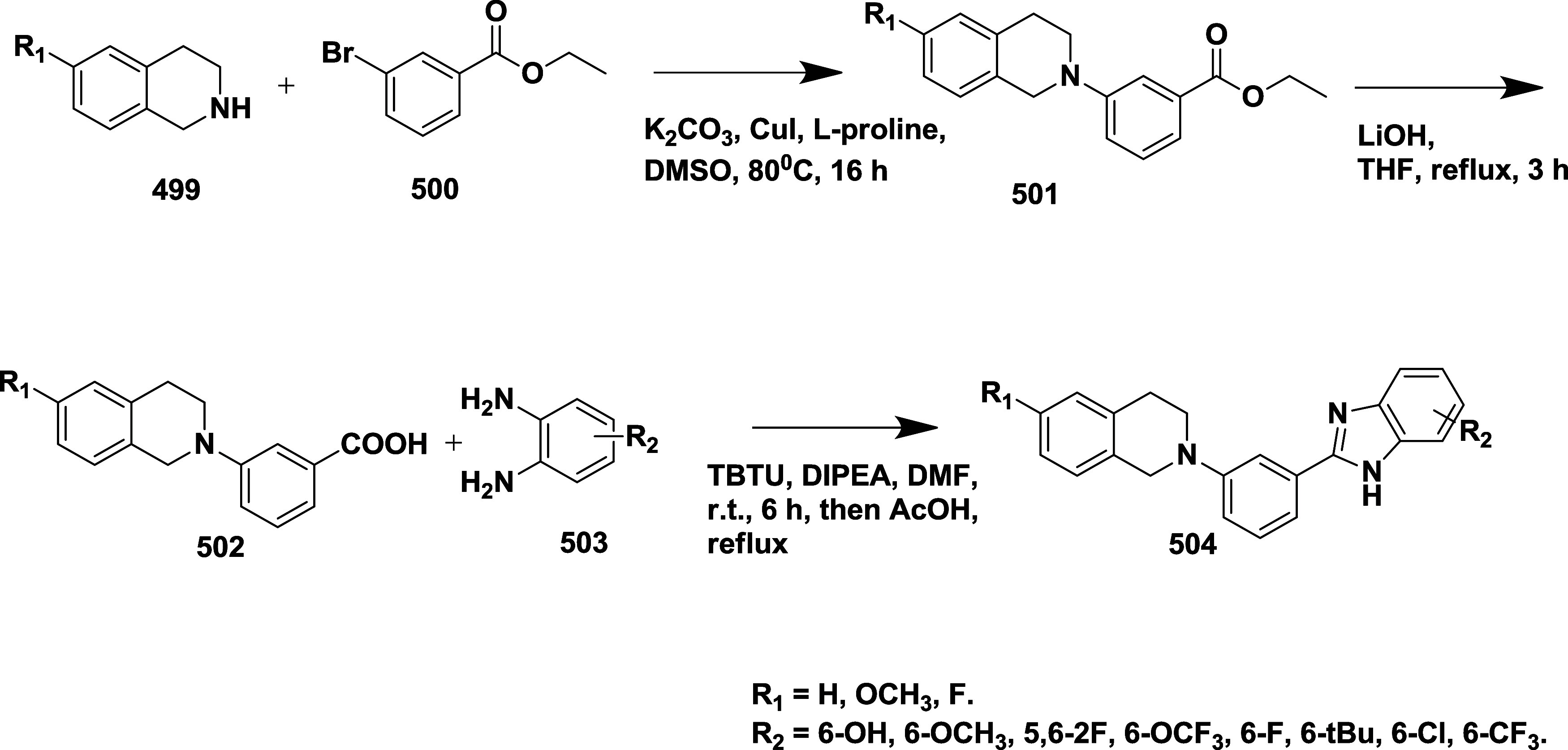
Synthesis of 2-(3-(1*H*-Benzo­[*d*]­imidazol-2-yl)­phenyl)-6-methyl-1,2,3,4-tetrahydroisoquinoline Derivatives **504**

Tran et al. reported the synthesis of a new series of chalcones^83^ through a Claisen-Schmidt reaction between benzaldehyde derivative **505** with N-substituted-4-acetophenothiazine **506** in the presence of sodium ethanolate as a catalyst, producing chalcone **507** with a yield ranging from 39–81% (Scheme 82). The structure–activity relationship of N-substituted-4-phenothiazine-chalcones revealed that both electronic characteristics and the ability to form key hydrogen bonds played a significant role in modulating AChE and BACE-1 inhibition. Among the series, compound **507** (with R_1_ and R_5_ as hydrogen, and R_2_, R_3_, R_5_ as methoxy groups, Table 6) exhibited the highest potency, achieving an IC_50_ of 0.16 μM. This strong inhibitory effect was attributed to favorable interactions with critical amino acid residues, namely Asp72 and Glu199 in AChE, and Asp93, Phe169, and Thr293 in BACE-1. Interactions such as hydrogen bonding and arene–cation or arene–arene stacking with residues like Trp84 and Glu199 were key contributors to AChE inhibition, while robust hydrogen bonding with Asp93 played a central role in BACE-1 targeting.

**Scheme 82:**
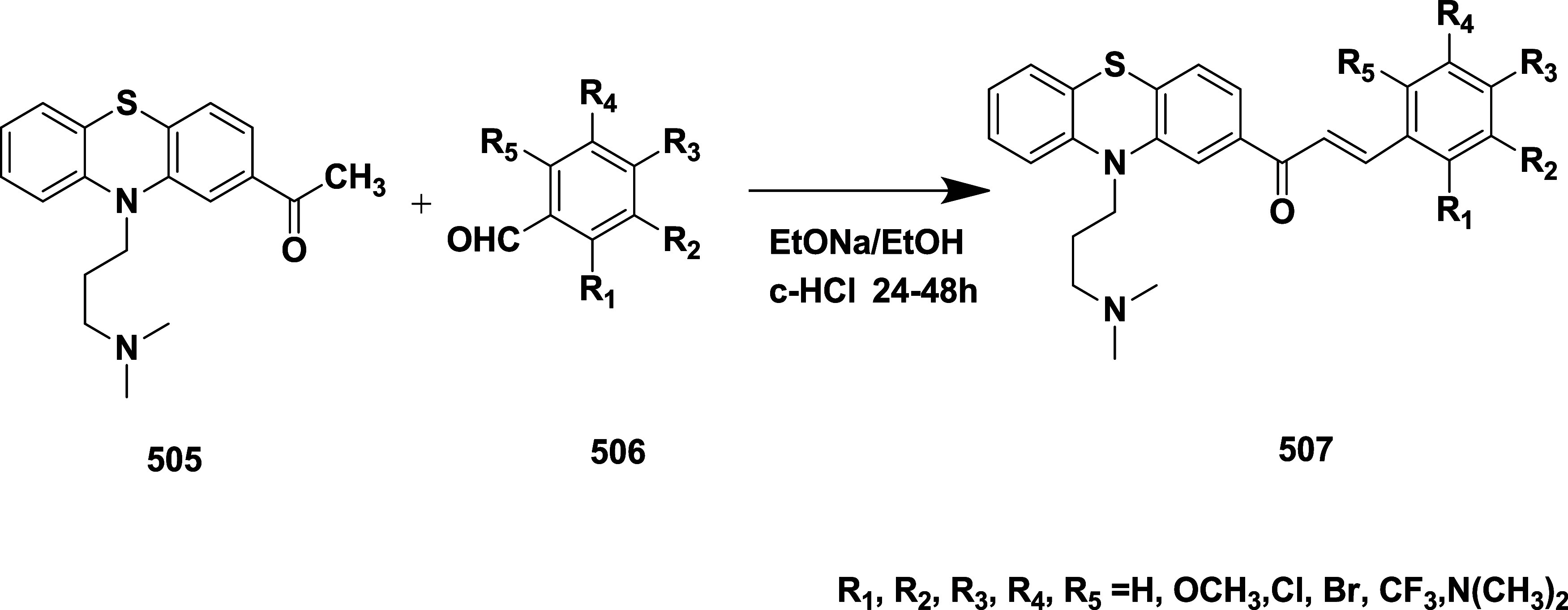
Synthesis of Thiazine Derivatives **506**

Choubey et al. reported the synthesis of new *N*-benzyl pyrrolidine derivatives as an inhibitor of β-secretase of Alzheimer’s disease,^84^ which involved the nucleophilic addition reaction of 1-benzyl-3-aminopyrrolidine **508** with substituted aromatic aldehydes **509** under reflux, formed the respective Schiff bases (methenamine derivatives, **510**) in ethanol. Further, Sodium borohydride reduction of Schiff bases **510** into methanamine derivatives **510** under cold conditions in methanol media (Scheme 83). The yield of synthesized compounds ranges from 61 to 93%. The SAR of *N*-benzylpyrrolidine derivatives showed that both the linker type and phenyl ring substituents greatly influenced inhibition of AChE, BChE, and BACE-1. Methanamine-linked compounds (**511**) were more potent than Schiff base analogs (**510**), especially when electron-withdrawing groups like 4-OCF_3_ and 2,4-diF were present. These substitutions led to strong multitarget inhibition in the nanomolar range, along with good BBB permeability and Aβ antiaggregation activity. Compounds with these features, particularly **511** derivatives, demonstrated potent BACE-1 inhibition (IC_50_ = 0.115 and 0.097 μM, Table 6) and promising neuroprotective properties. Docking studies confirmed strong binding at AChE’s PAS and BACE-1’s catalytic dyad.

**Scheme 83:**
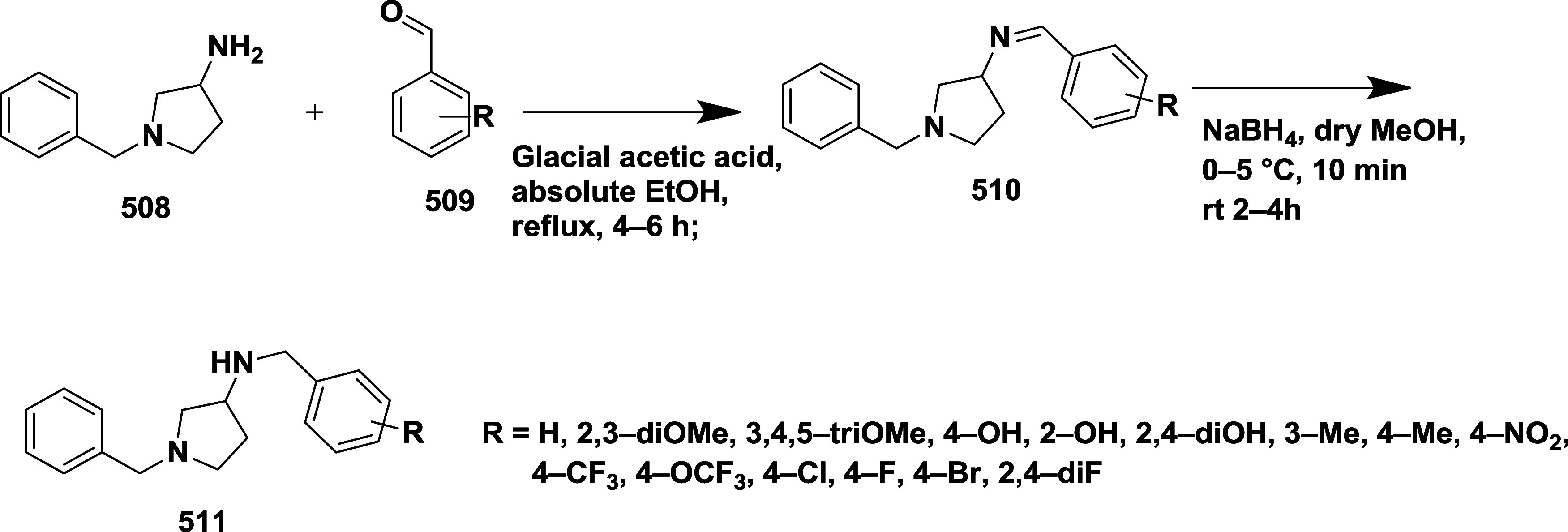
Synthesis of *N*,1-Dibenzylpyrrolidin-3-amine Derivatives **511**

Sakata et al. reported the synthesis of 2′-aminochalcone as a multitarget inhibitor for β-secretase inhibitors^85^ At room temperature, the Claisen–Schmidt condensed various aldehydes **512** with 2-aminoacetophenone **513** in sodium hydroxide in methanol, producing amino chalcones **514**. The (*E*)-2-((2-(3-(4-chlorophenyl) acryloyl)­phenyl)­amino) acetic **516** acid was produced from (*E*)-1-(2-aminophenyl)-3-(4-chlorophenyl) prop-2-en-1-one **515** reacting with α-bromomethyl acetate in dry tetrahydrofuran, under nitrogen atmosphere, at room temperature (Scheme 84). The yield of synthesized compounds ranges from 27 to 88%. The structure–activity relationship of 2′-aminochalcones revealed that the amino group at the 2′-position is vital for BACE-1 inhibition, as analogs lacking this functionality showed no activity. In this series, the 2,3-dichlorophenyl-substituted compound **514** exhibited the strongest dual inhibitory effect on AChE and BACE-1, with IC_50_ values of 0.08 and 2.71 μM, respectively, supported by molecular docking that demonstrated key interactions with BACE-1’s catalytic dyad. Another variant of compound **514** bearing a 4-chlorophenyl group showed the highest inhibition of Aβ fibril formation (52%) along with moderate enzyme inhibition. A glycinate-conjugated derivative of this compound further enhanced enzyme inhibition while retaining good antiaggregation and low cytotoxicity, emphasizing the role of electron-withdrawing groups and the 2′-amino moieties in achieving effective multitarget activity for Alzheimer’s treatment.

**Scheme 84:**
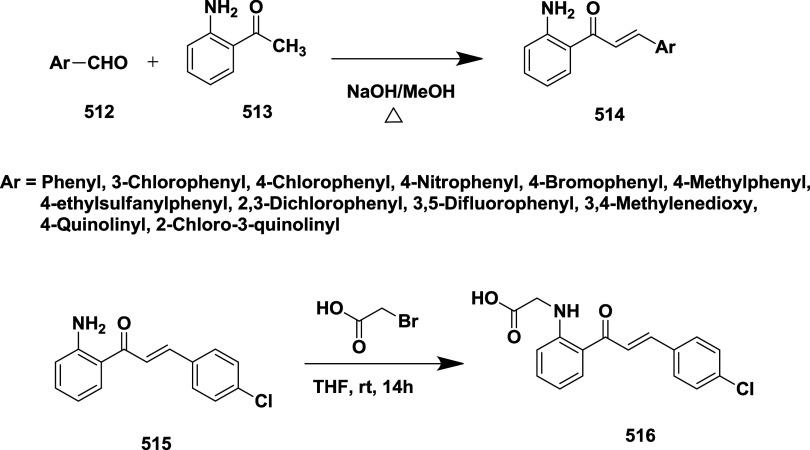
Synthesis of Aminochachones **514** and (*E*)-2-((2-(3-(4-Chlorophenyl)­acryloyl)­phenyl)­amino) Acetic Acid **516**

Agbo et al. reported the synthesis of furocoumarin–stilbene hybrids as multitarget inhibitors for AD.^86^ Synthesis involves the Arbuzov reaction of compound **517** with triethyl phosphite under reflux, resulting in diethyl (4*H*-chromen-4-yl) methyl ((7-hydroxy-8-iodo-2-oxo) phosphonate) **518**. By Horner-Emmons olefination of **517** with 3,5-dimethoxybenzaldehyde in tetrahydrofuran (THF) at room temperature, produced the (*E*)-4-(3,5-dimethoxy styryl)-7-hydroxy-8-iodo-2H-chromen-2-one **519**. The corresponding novel furocoumarin derivatives **520** were made by Cacchi-type cycloisomerization of **519** with terminal acetylenes following Palladium-catalyzed Sonogashira cross-coupling (Scheme 85). The yield of synthesized compounds ranges from 72 to 87%. The structure–activity relationship of furocoumarin–stilbene hybrids demonstrated that modifications at the 8-position of the furocoumarin ring play a key role in modulating inhibition of BACE-1, AChE, and BChE. Derivatives **520** with para-halogenated phenyl groups, such as 4-fluoro and 4-chloro substituents, showed the strongest dual inhibition of AChE and BChE, attributed to enhanced π-electron delocalization and efficient active site binding. Incorporation of a 3,5-dimethoxyphenyl group at the same position enhanced AChE inhibition but slightly compromised BChE activity, possibly due to steric limitations in the BChE active site. One of the compounds in this series also showed notable inhibitory effects on BACE-1 (IC_50_ = 3.2 μM, Table 6), COX-2, and LOX-5, and displayed strong antioxidant and ROS-scavenging properties. These findings suggest that para-substituted phenyl rings and a balanced combination of hydrophobic and electronic features at the 8-position contribute to optimal multitarget activity for Alzheimer’s therapy.

**Scheme 85:**
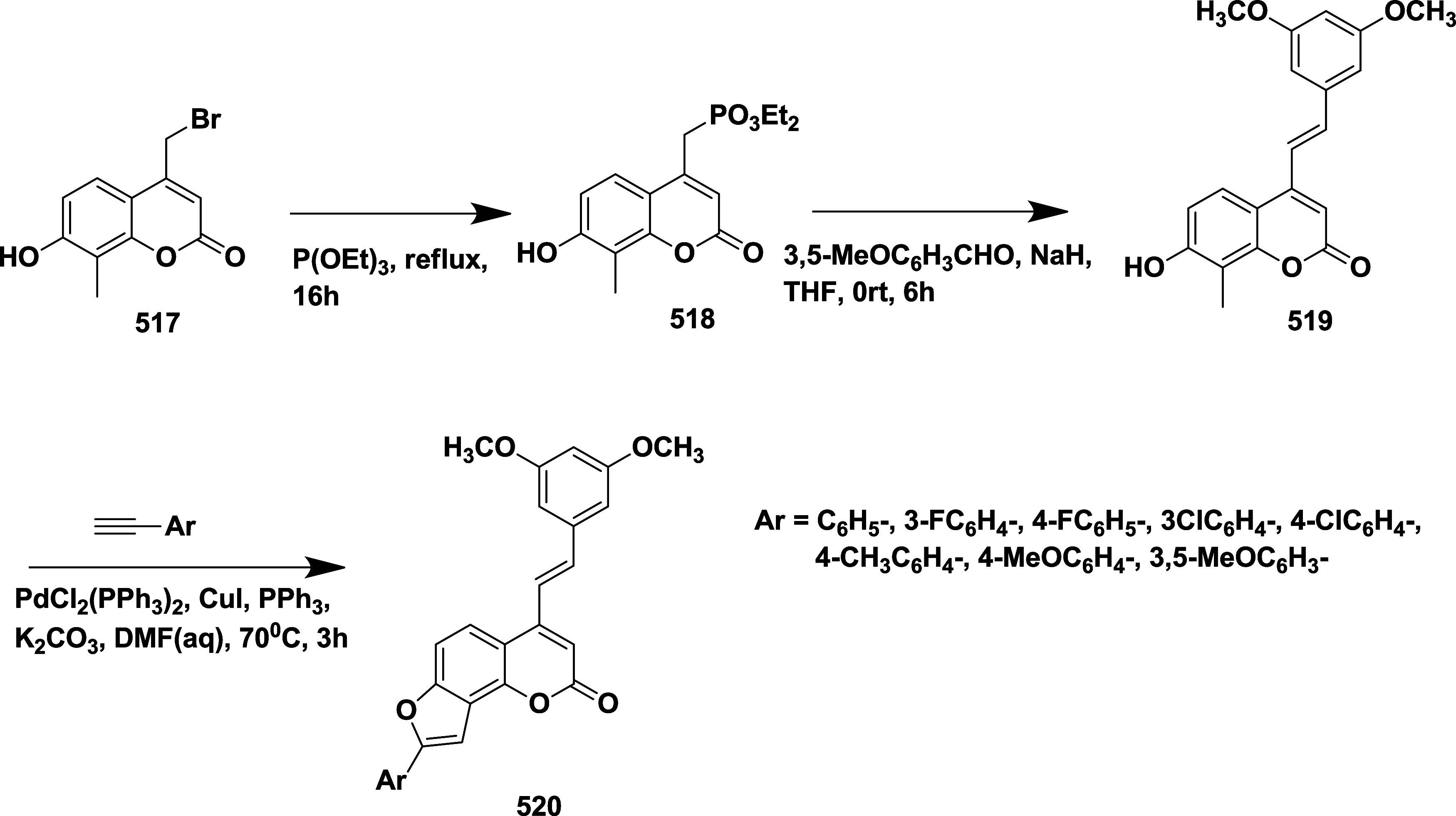
Synthesis of (*E*)-8-Argio-4-(3,5-dimethoxystyryl)-2*H*-furo­[2,3-*h*] Chromen-2-one Derivatives **520**

Calugi et al. reported the synthesis of new morpholine derivatives as BACE1 inhibitors,^87^ which involves the synthesis of substituted morpholinone compounds **523** via Castagnoli Cushman reaction between di glycolic anhydride **521** and various imines **522** (Scheme 86). In Scheme 87, synthesizing the target compounds 3-amino-1,4-oxazines 534 began with morpholinones **524**. Refluxing it with trifluoroacetic acid eliminated the *t*-butyl group, yielding an intermediate **525**. Lawesson’s reagent effectively converted the resultant amides **525** into the corresponding thioamides **526** (55–80%), and aminolysis of **526** with 7 N ammonia solution in methanol produced the 3-amino-1,4-oxazines **527**, offering a moderate yield ranging from 50–70% (Scheme 87). The SAR of morpholine-based derivatives prepared via the Castagnoli–Cushman reaction emphasized the crucial role of the thioamide functionality in BACE-1 inhibition. Thioamide compounds (**526**s) exhibited significantly greater activity than their amidine counterparts (**527**s), with the most potent compound bearing methoxy and hydrogen substituents (R_1_ = OMe, R_2_ = H), achieving an IC_50_ of 5.7 μM (Table 6). Para-substituted phenyl derivatives were generally more effective than those with meta substitution, deviating from previously observed trends. Molecular docking revealed that key hydrogen bonds and π–π stacking with residues like Asp228 and Arg235 contributed to stable binding within the BACE-1 active site. These findings support cyclic thioamides as promising lead structures for developing brain-penetrant BACE-1 inhibitors.

**Scheme 86:**
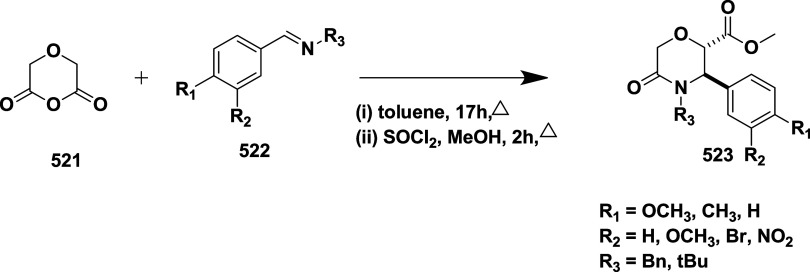
Synthesis of (2*S*,3*R*)-Methyl 5-Oxo-3-phenylmorpholine-2-carboxylate Derivatives **523**

**Scheme 87:**
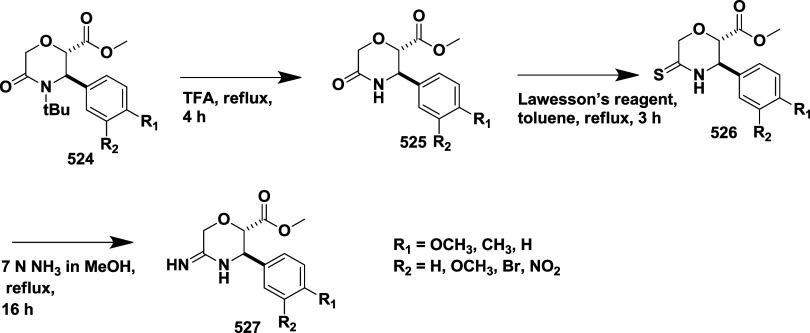
Synthesis of Morpholine-3-thiones **533** and 3-Amino-1,4-oxazine Derivatives **527**

**Scheme 88:**
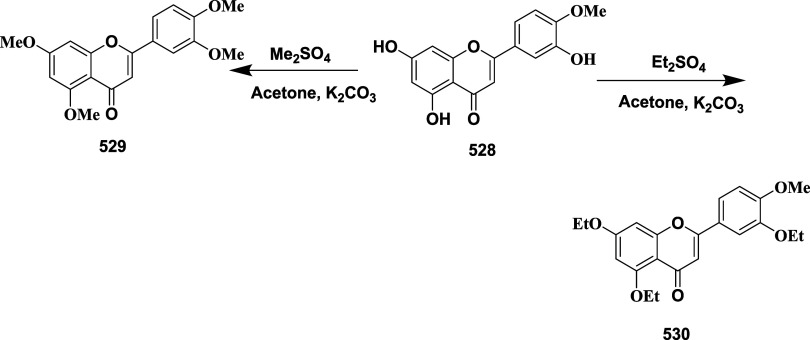
Synthesis of 5,6,7-Trihydroxy Phenyl Substituted Chromen-4-one **529** and **530**

Tran et al. synthesized new flavones^88^ as dual inhibitors of AChE and BACE-1. Etherification of 5,7-dihydroxy-2-(3-hydroxy-4-methoxyphenyl)-4*H*-chromen-4-one **528** with dimethyl sulfate and diethyl sulfate in the presence of potassium carbonate obtained product 5,7-diethoxy-2-(3-ethoxy-4-methoxyphenyl)-4*H*-chromen-4-one **529** and 2-(3,4-dimethoxyphenyl)-5,7-dimethoxy-4*H*-chromen-4-one **530** with a yield of 42 and 35% respectively (Scheme 88). Similarly, 14 flavone derivatives were synthesized with a yield of 35 to 85%. The SAR analysis of flavone derivatives revealed that substitutions at the 5,6,7 and 3′,4′ positions play a key role in modulating dual inhibition of AChE and BACE-1. Compounds **529** and **530** emerged as the most effective BACE-1 inhibitors, with pIC_50_ values reaching 5.80, attributed to favorable hydrogen bonding and van der Waals interactions within the enzyme’s active site. Diosmetin-based derivatives demonstrated greater BACE-1 activity compared to baicalein analogs, particularly when substitutions allowed interactions with residues such as Thr232. Molecular docking results closely aligned with experimental data (*R* ^2^ ≈ 0.8), emphasizing the importance of interactions near the Asp32 and Asp228 catalytic dyad. These findings highlight the potential of appropriately substituted flavones as multitarget agents for Alzheimer’s therapy.

Ramrao et al. reported the synthesis of novel biphenyl imidazole derivatives as β-secretase inhibitors.^89^ Compound **531** was the key starting material for synthesizing the target compounds **(534**, **535**, **536)**, which reacted with bromo-chloro substituted alkanes, dry potassium carbonate in acetonitrile to yield intermediates **532**. Key intermediates **533** were made by refluxing 533 with benzoin in the presence of ammonium acetate and ceric ammonium nitrate in ethanol and water. The desired compounds (**534**, **535**, **536**) were obtained by refluxing the intermediates **533** with various substituted piperazines in the presence of potassium iodide and anhydrous potassium carbonate in acetonitrile, with yields in the range of 62–73% (Scheme 89). The structure–activity relationship of biphenyl imidazole–piperazine hybrids demonstrated that both the length of the connecting chain and the nature of terminal substitutions significantly influenced their inhibitory effects on AChE, BChE, and BACE-1. Derivatives containing a two-carbon linker were generally more effective than those with a three-carbon bridge. Notably, compound **535** (*n* = 2), which includes a benzyl-substituted piperazine and a two-carbon spacer, exhibited the highest potency, with IC_50_ values of 0.416 μM for AChE, 0.474 μM for BChE, and 0.392 μM for BACE-1. The introduction of methoxy and fluoro groups further improved activity, likely due to enhanced binding within enzyme active sites. Computational studies supported these findings, showing that compound **535** (*n* = 2) binds stably within both the peripheral and catalytic sites of AChE and interacts moderately with BACE-1, highlighting its promise as a multitarget agent for Alzheimer’s disease.

**Scheme 89:**
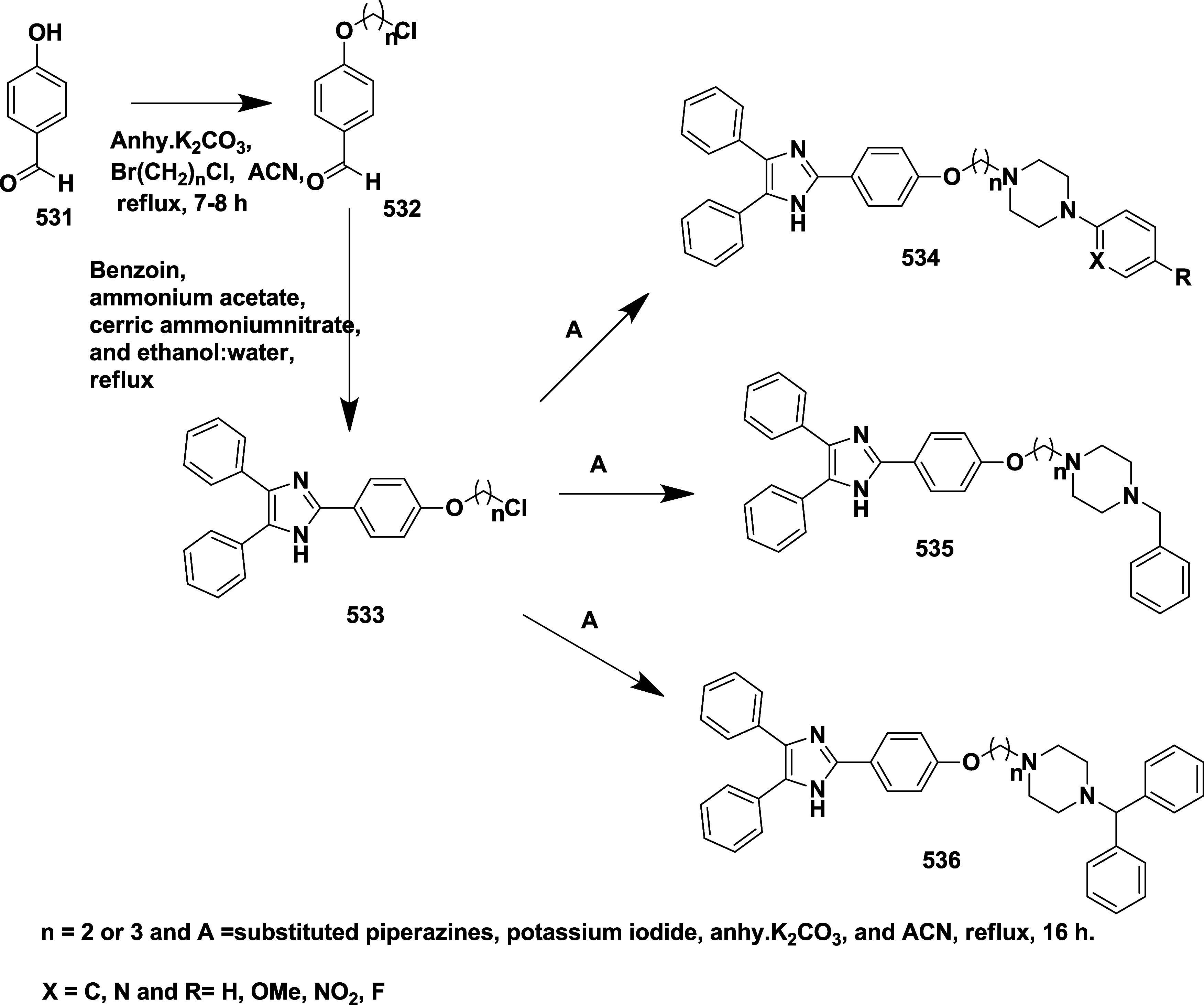
Synthesis of Biphenyl Imidazole Derivatives **534**, **535**, and **536**

Choubey et al. synthesized new *N*-benzyl pyrrolidine and 1,3,4-oxadiazole as a multitarget inhibitor for AD.^90^ The synthesis involved the pyridine-catalyzed reaction of 1-benzylpyrrolidin-3-amine **537** with phenyl chloroformate **538** in dry DCM to produce carbamate intermediate **539**. Compound **539** was refluxed with hydrazine hydrate to give intermediate **540**, followed by condensation of **540** with various aromatic aldehydes **541** in absolute ethanol in the presence of a catalytic amount of glacial acetic acid to produce semicarbazones **542** (71–78%). Further, using dry sodium borohydride, **542** were reduced into corresponding semicarbazone derivatives **543** (51–58% yield) under cold conditions in methanol media. Finally, the desired target molecules **544** (73–81% yield) were synthesized by oxidative cyclization **542** in the presence of diacetoxyiodobenzene (Scheme 90).

**Scheme 90:**
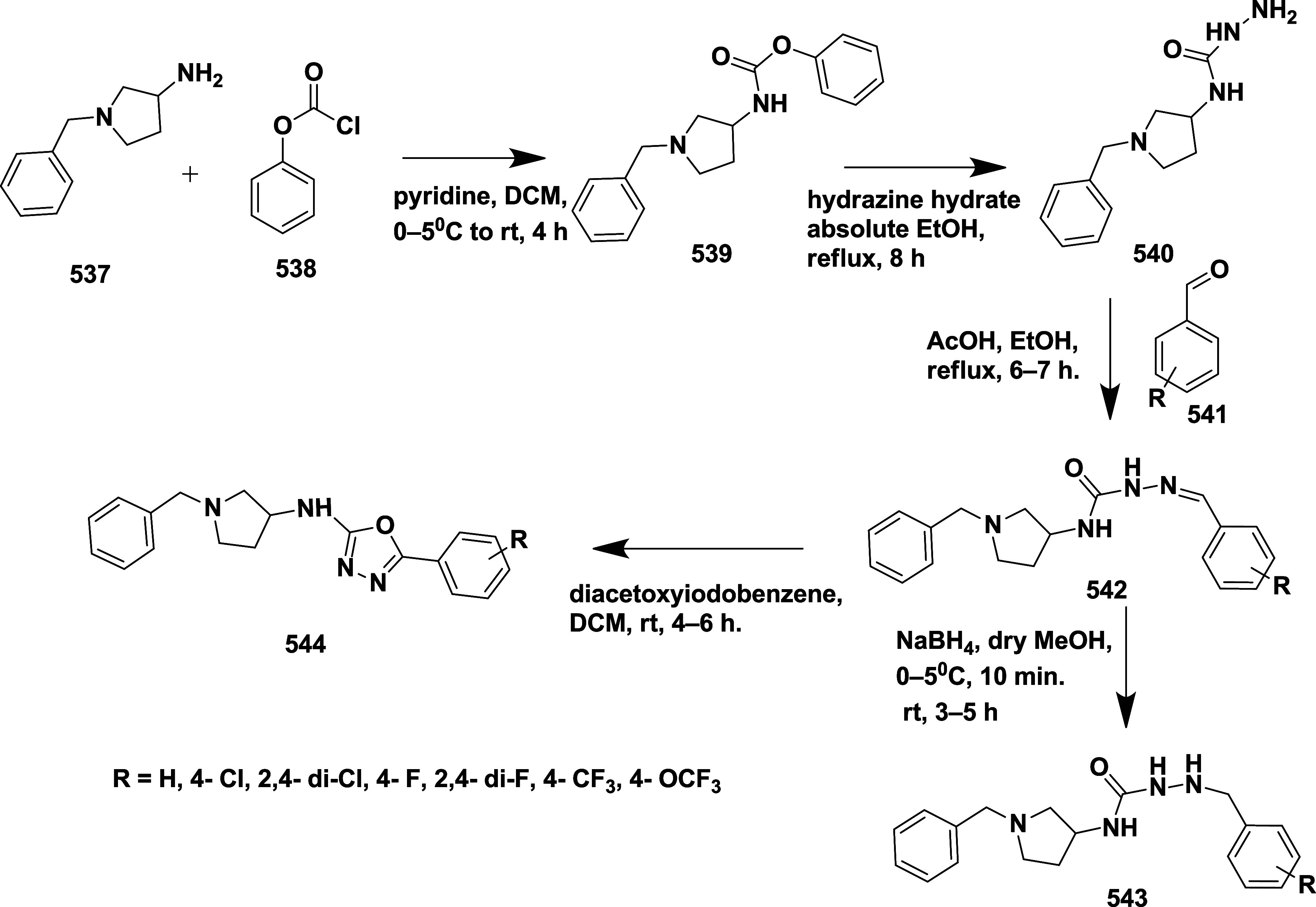
Synthesis of (*Z*)-2-Benzylidene-*N*-(1-benzylpyrrolidin-3-yl) Hydrazine Carboxamide Derivatives **542**, **543**, and **544**

Initially, *N*-hydroxybenzotriazole (HOBT) and 1-ethyl-3-(3-(dimethylamino) propyl)-carbodiimide (EDC) were reacted in acetonitrile to esterify the substituted benzoic acid derivatives **545**. The resulting benzoic acid esters were treated with hydrazine hydrate under cold conditions to yield intermediate **546**. Intermediates **547** are obtained by cyclizing intermediate **546** by heating chloroacetic acid at reflux in POCl_3_. Finally, the targeted series of compounds **548** (47–63% yield) was synthesized via a reaction of **547** with 1-benzylpyrrolidin-3-amine in the presence of potassium iodide in dimethyl fluoride (Scheme 91). The structure–activity relationship of *N*-benzylpyrrolidine–1,3,4-oxadiazole hybrids showed that both the nature of the linker (−NH versus −NHCH_2_) and the presence of electron-withdrawing groups on the aromatic ring played a crucial role in modulating inhibition of AChE, BChE, and BACE-1. Cyclic oxadiazole compounds displayed superior potency compared to their open-chain counterparts, with compound **548**, bearing a CF_3_ group, achieving potent submicromolar inhibition across all three targets. Electron-withdrawing substituents such as CF_3_ and OCF_3_ were found to enhance not only enzyme inhibition but also Aβ antiaggregation, displacement from AChE-PAS, blood–brain barrier penetration, and neuroprotection. Compound **548** bearing a CF_3_ group showed excellent pharmacokinetic behavior and led to measurable cognitive improvement in animal studies. These results underscore the importance of careful linker selection and substituent optimization in the design of effective multitarget agents for Alzheimer’s disease .

**Scheme 91:**
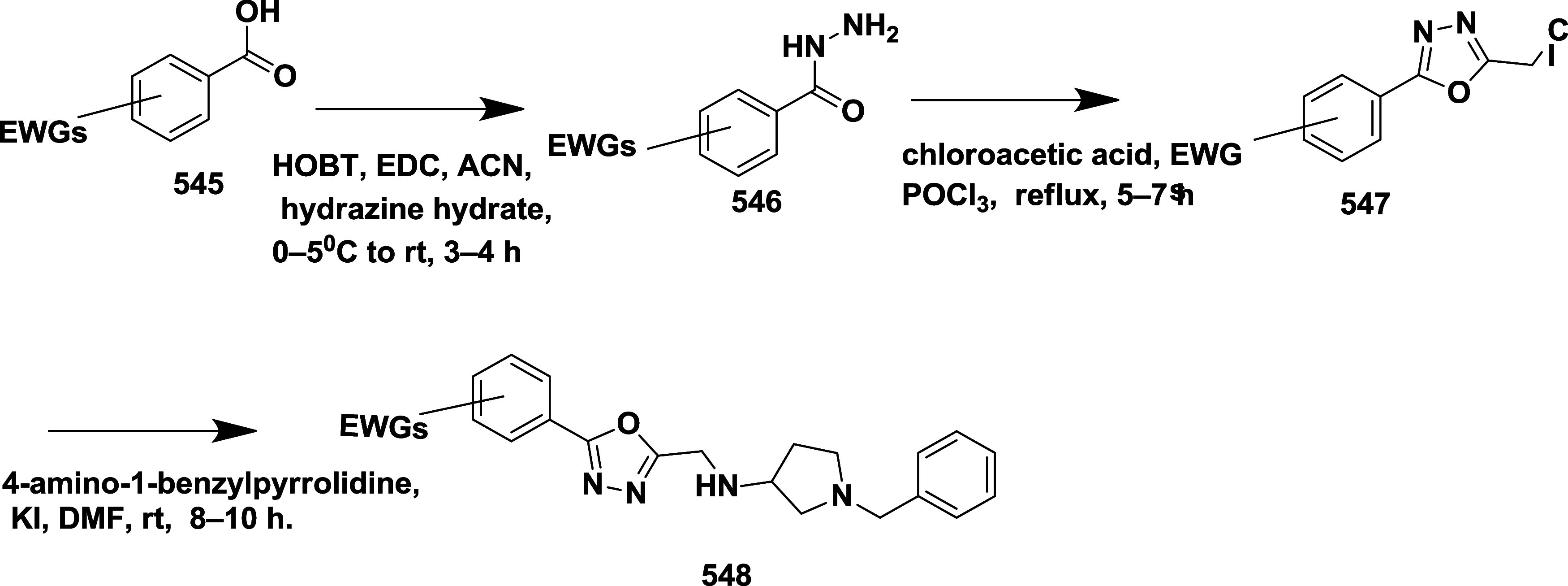
Synthesis of 1-Benzyl-*N*-((5-phenyl-1,3,4-oxadiazol-2-yl) methyl) Pyrrolidin-3-amine Derivatives **548**

Mathew et al. synthesized a new series of piperazine-substituted chalcones as BACE-1 inhibitors.^91^ The Claisen-Schmidt condensation reaction between 4-piperzinoacetophenone **549** and various aromatic aldehydes **550** yields the desired compounds **551** (Scheme 92). Structure–activity relationship (SAR) analysis of piperazine-based chalcone derivatives indicated that substitutions on the B-ring play a crucial role in modulating biological activity. The entire series exhibited greater selectivity toward monoamine oxidase B (MAO-B) over MAO-A, with compounds bearing para-fluoro and para-trifluoromethyl groups, specifically compound **551**, showing the highest MAO-B inhibitory potency (IC_50_ = 0.65 and 0.71 μM, respectively). These findings emphasize the beneficial effect of electron-withdrawing groups at the para position of the aryl B-ring. Compounds incorporating electron-donating substituents such as methyl, hydroxyl, and methoxy displayed moderate inhibitory activity against acetylcholinesterase (AChE), with the methoxy-substituted analogue yielding the strongest AChE inhibition (IC_50_ = 8.77 μM). Furthermore, compounds substituted with para-methoxy, para-fluoro, and para-trifluoromethyl groups also demonstrated modest inhibition of β-secretase (BACE-1), with IC_50_ values of 6.72, 14.9, and 15.3 μM, respectively. The SAR data also suggest that fluorinated derivatives exhibit stronger MAO-B activity compared to chloro or bromo-substituted analogues, likely due to enhanced hydrophobic interactions within the enzyme’s active site. These results underscore the significance of combining a piperazine scaffold with strategically substituted aryl moieties to develop multitarget-directed ligands (MTDLs), particularly for the inhibition of MAO-B and BACE-1, offering potential in the treatment of neurodegenerative conditions such as Alzheimer’s disease. The structure of a potent BACE-1 inhibitor is listed in Table 6.

**Scheme 92:**
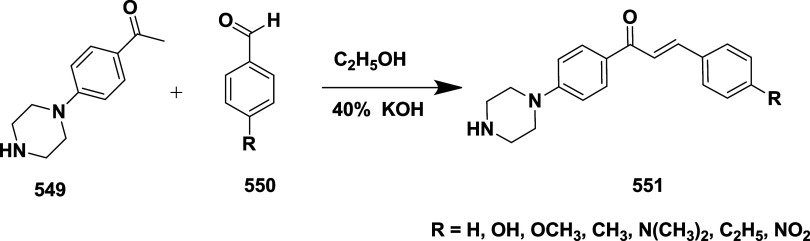
Synthesis of Piperazine-Substituted Chalcones **551**

The synthesis of 2-aminotetrahydropyridine-based β-secretase, reported by Rombouts et al., targets the S3 pocket of the enzyme.^92^ The synthesis begins with the oxidation of the primary alcohol of key starting material 552 using trichloisocyanuric acid and TEMPO, yielding aldehyde 553, followed by the condensation of the aldehyde with ethyl sulfonyl acetonitrile in the presence of 1,8-diazbicyclo[5.4.0]­undec-7-ene, and subsequent reduction by using sodium borohydride, yielding compound **555**. Methylation of **555** and removal of Boc using hydrochloric acid afforded the desired stereomer, which was selectively recrystallized from isopropanol to produce **557**. The cyclization of **557** using trifluoroacetic acid, followed by nitration and Pd/C hydrogen-catalyzed reduction of the nitro group, produced compound **560**. Finally, the carboxylic tail **561** was coupled with **560** in the presence of EDC·HCl/hydrochloric acid gave the final compound JNJ67569762 (**562**) in 75% yield (Scheme 93). SAR studies of 2-aminotetrahydropyridine-based BACE-1 inhibitors focused on enhancing potency, brain penetration, metabolic stability, and selectivity over BACE-2. Structural modifications targeted the S3 pocket by leveraging differences in the 10S loop between BACE-1 and BACE-2, leading to improved binding profiles. A fused pyrido-1,4-dioxane tail significantly boosted activity but raised concerns related to liver toxicity. Further refinement of the core structure, particularly through the addition of an ethylsulfonyl group, produced JNJ-67569762 (compound **562**, Table 6), which showed strong BACE-1 inhibition (IC_50_ = 2.7 nM), high selectivity, and favorable CNS and metabolic properties. These outcomes highlight the importance of fine-tuning electronic and structural features to achieve safe and effective BACE-1 inhibitors.

**Scheme 93:**
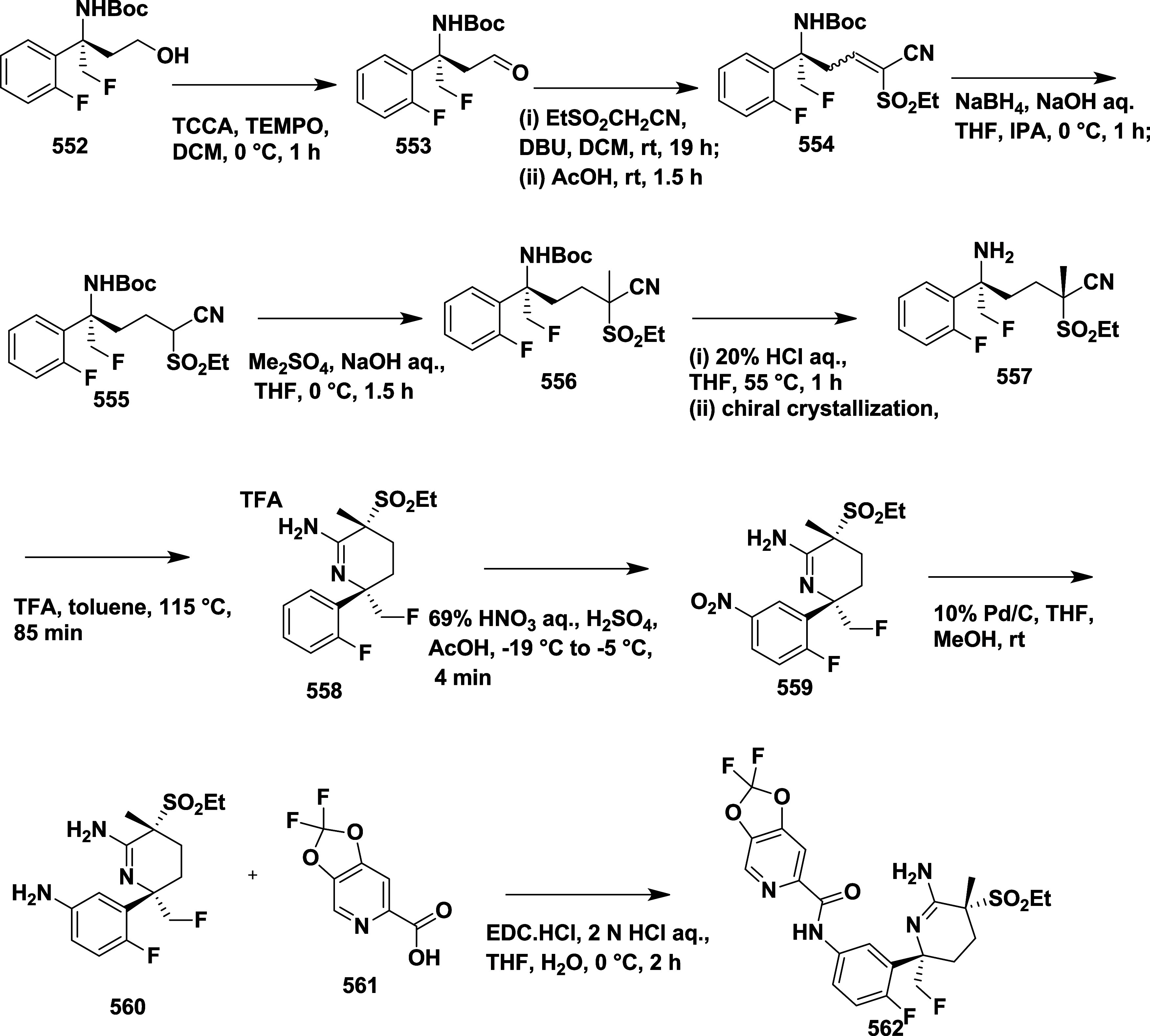
Synthesis of Compound **562** (JNJ-67569762)

Pan et al. reported synthesizing and evaluating new potent β-site APP cleaving enzyme inhibitors.^93^ First, synthesizing carbamate **564** from a reaction of **563** with Meldrum acid and DAMP at room temperature, followed by refluxing with toluene, which then reacted with HOBT and EDCl, yielded an intermediate **566**. The reaction of **566** with trifluoracetic acid offered **567**. The 5-(3-methoxyphenyl)-2-nitrobenzaldehyde **569**, obtained from 5-fluoro-2-nitrobenzaldehyde, reacts with 3-methoxyphenyl in potassium carbonate. The reaction of **567** with **569** in sodium cyanoborohydride, along with acetic acid, offered (*S*)-*N*,4-dicyclohexyl-4-((5-(3-methoxyphenyl)-2-nitrobenzyl) amino)-*N*-methyl butanamide **570**. The Pd/C catalyzed reduction of **570**, subsequently by reacting with boron tribromide, yielded an intermediate (*S*)-4-(2-amino-6-(3-hydroxyphenyl) quinazoline-3 (4*H*)-yl)-*N*,4-dicyclohexyl-*N*-methylbutanamide hydrobromide **572**. The reaction of **572** with 3-fluoropropyl 4-methylbenzenesulfonate and propane-1,3-diyl bis­(4-methylbenzenesulfonate) in the presence of TBAOH at room temperature and potassium carbonate at 90 °C offered (*S*)-4-(2-amino-6-(3-(3-fluoropropoxy)­phenoxy)­quinazolin-3­(4*H*)-yl)-*N*,4-dicyclohexyl-*N*-methylbutanamide **573** and (*S*)-3-(3-((2-amino-3-(1-cyclohexyl-4-(cyclohexyl­(methyl)­amino)-4-oxobutyl)-3,4-dihydroquinazolin-6-yl)­oxy)­phenoxy)­propyl 4-methylbenzenesulfonate **574** yielded 60.9 and 66% respectively (Scheme 94). Structure–activity relationship of a 2-amino-3,4-dihydroquinazoline-based inhibitor, which demonstrated high affinity for BACE-1 (*K_i_* = 11 nM). To develop a PET-compatible probe, researchers modified the scaffold by introducing a meta-fluoropropyl group on the phenyl ring, allowing radiolabeling with fluorine-18 and yielding compound **573** (Table 6 represents the structure). Despite retaining strong inhibitory activity (IC_50_ = 13.2 nM), the compound exhibited limited brain uptake in rodent and primate imaging studies. The limited CNS penetration was attributed to unfavorable physicochemical properties, including elevated molecular weight, high lipophilicity, and an increased topological polar surface area. These findings underscore the critical need to optimize such parameters when designing radioligands for central nervous system imaging. Refining these characteristics may lead to the development of more effective PET tracers capable of accurately targeting and monitoring BACE-1 activity in vivo.

**Scheme 94:**
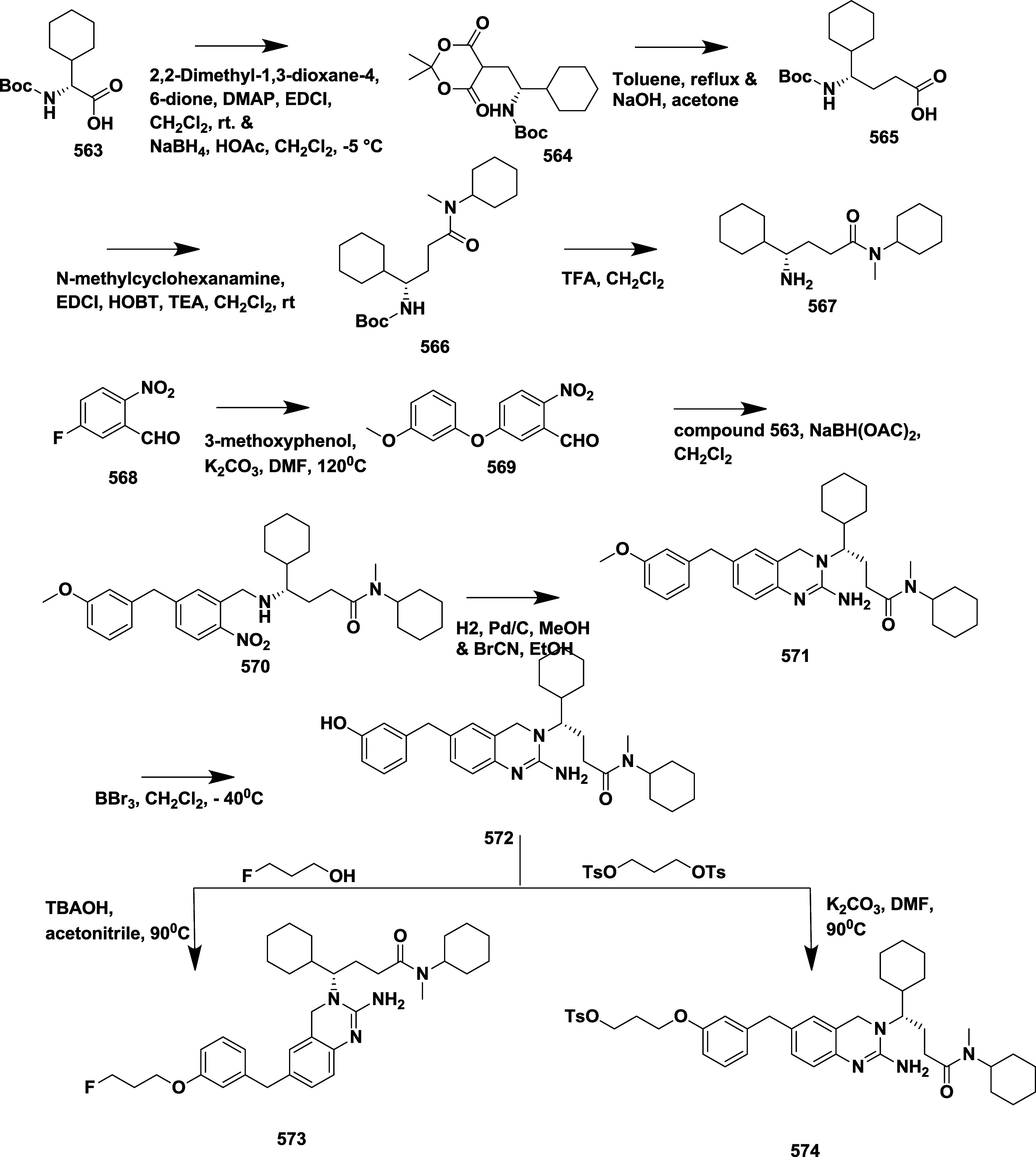
Synthesis of (*S*)-4-(2-Amino-6-(3-(3-fluoropropoxy)­benzyl)­quinazolin-3­(4*H*)-yl)-*N*,4-dicyclohexyl-*N*-methylbutanamide **573** and (*S*)-3-(3-((2-Amino-3-(1-cyclohexyl-4-(cyclohexyl­(methyl)­amino)-4-oxobutyl)-3,4-dihydroquinazolin-6-yl)­methyl)­phenoxy)­propyl 4-Methylbenzenesulfonate **574**

Tok et al. reported the synthesis of new nicotinohydrazide derivatives as multitarget inhibitors of AD.^94^ First, the reaction of methyl 6 6-chloropyridine-3-carboxylate **571** and hydrazine in ethanolic media produced an intermediate 6-chloronicotinohydrazide **576**. This hydrazide intermediate condensed with various aromatic benzaldehydes by refluxing to get target compound **577**, yielding 80% (Scheme 95). Among these compounds, the inhibitor **577** (if R = 4-NO_2_) exhibits notable β-secretase inhibition activity with an IC_50_ value found to be 0.205 μM (Table 6). The SAR study of nicotinohydrazide derivatives revealed that both the type and position of substituents on the benzylidene ring significantly impacted their inhibitory activity against AChE, BChE, and BACE-1. Compounds bearing electron-withdrawing groups, particularly the para-nitro substituent, exhibited the most potent dual inhibition, with activity comparable to known drugs like donepezil and verubecestat. Derivatives containing hydroxyl, methoxy, or ethoxy groups also showed strong activity, likely due to their ability to form hydrogen bonds with enzyme active sites. Molecular docking supported these findings, showing favorable interactions at both catalytic and peripheral sites of AChE and key binding interactions within the BACE-1 pocket. Overall, the results highlight the potential of para-substituted electron-withdrawing and hydrogen-bonding groups in enhancing target affinity, supporting their use in designing multifunctional agents for Alzheimer’s therapy.

**Scheme 95:**
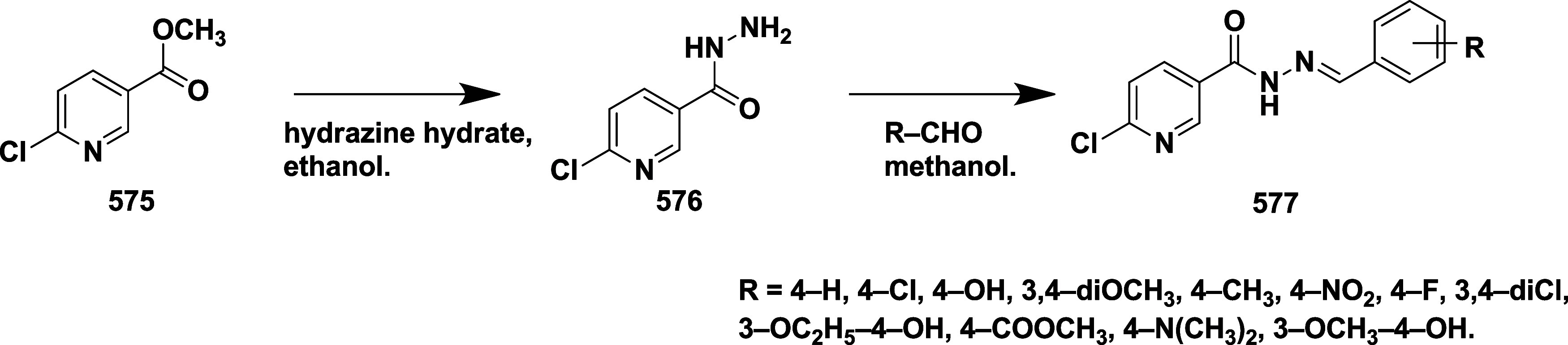
Synthesis of Hydrazone Derivatives **577**

Matošević et al. reported the synthesis of new 4-aminoquinolines as β-secretase inhibitors.^95^ The synthesis begins with the reaction between 4-chlorquinoline derivatives **578** and neat 1,8-diamino octane at 130 °C under argon atmosphere, produced an intermediate **579**, followed by a reaction of **579** with various aryl-aldehydes and reductive amination under argon atmosphere, to obtain desired target compounds **580** with yields in the range of 39–90% (Scheme 96). These 4-aminoquinolines containing fluorine on the terminal benzyl group showed 18% inhibition of BACE-1 activity and highly potent and nonselective inhibition of BChE and AChE. The compound **580** (if R = Cl, R_1_ = H, and R_2_ = F) showed good inhibition among this series of 4-aminoquinolines (Table 6). The SAR analysis of aminoquinoline derivatives highlighted how specific substitutions influence BACE-1 activity and selectivity. Halogen or CF_3_ substitutions at the C(7) position or terminal *N*-benzyl group reduced AChE inhibition, indicating lower off-target cholinergic effects. Incorporating heterocyclic groups like pyridine or imidazole at the terminal end further decreased BChE activity, enhancing selectivity. While halogenation was associated with reduced cytotoxicity, CF_3_ groups increased toxicity, especially in neuronal and kidney cells. However, both halogens and bulky electron-withdrawing groups were found to negatively affect BACE-1 inhibition, suggesting that careful optimization of substituents is essential to balance potency, selectivity, and safety.

**Scheme 96:**
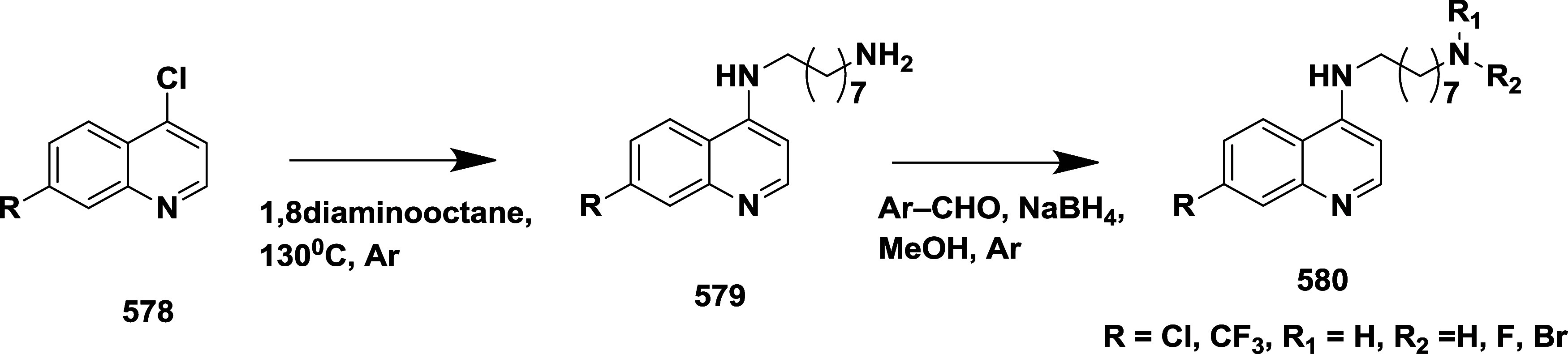
Synthesis of 4-Aminoquinoline Derivatives **580**

## Conclusions

In recent years, notable advancements have been achieved in designing and synthesizing β-secretase (BACE-1) inhibitors aimed at treating Alzheimer’s disease (AD). Diverse synthetic methodologies have facilitated the development of novel scaffolds such as aminohydantoins, iminopyrimidinones, dihydropyridines, and various fused heterocycles. These efforts have produced compounds exhibiting enhanced potency, selectivity, central nervous system (CNS) penetration, and favorable pharmacokinetic characteristics. Structure–activity relationship (SAR) analyses have been instrumental in fine-tuning interactions within the BACE-1 active site, addressing key challenges including limited brain exposure, off-target effects, particularly cathepsin D inhibition, and metabolic instability. In addition, using rigidified molecular frameworks and optimized physicochemical profiles has contributed to identifying promising clinical candidates, such as LY3202626 and MBi-4, which have shown both efficacy and safety in in vivo models. Although no BACE-1 inhibitors have gained regulatory approval, the accumulated synthetic knowledge and mechanistic understanding offer a robust platform for ongoing and future drug discovery. Progress in this field will continue to rely on integrated approaches that unite synthetic chemistry, structural insights, and pharmacological evaluation to unlock the therapeutic potential of BACE-1 inhibition in Alzheimer’s disease.

## Data Availability

The data from the literature have been used and discussed within the manuscript.
